# Cooperation, competition and enforcement in transposon evolution

**DOI:** 10.1038/s41467-026-75312-1

**Published:** 2026-08-12

**Authors:** Nicholas G. Davies, J. Arvid Ågren, Kevin R. Foster

**Affiliations:** 1 Department of Infectious Disease Epidemiology, London School of Hygiene and Tropical Medicine, London, UK; 2 Department of Biology, University of Oxford, Oxford, UK; 3 Department of Evolutionary Biology, Uppsala University, Uppsala, Sweden; 4 Cleveland Clinic Lerner College of Medicine, Case Western Reserve University, Cleveland, OH USA; 5 Department of Genomic Sciences and Systems Biology, Cleveland Clinic Research, Cleveland, OH USA; 6 Department of Biochemistry, University of Oxford, Oxford, UK; 7 Sir William Dunn School of Pathology, University of Oxford, Oxford, UK

**Keywords:** Social evolution, Population genetics, Coevolution

## Abstract

Transposons are powerful drivers of genome evolution, but we lack a clear understanding of how these selfish genetic elements evolve and co-evolve with their hosts. Here, we develop a new general model of transposon–host co-evolution that incorporates key details of transposon and host biology. Our model reveals that the way that transposons replicate is critical for their evolutionary prognosis. Publicly-replicating transposons (such as DNA transposons), which cooperatively share their replication machinery, are predicted to be self-limiting. However, privately-replicating transposons (such as long interspersed nuclear elements, or LINEs), which do not replicate cooperatively, are under continual selection to increase their duplication rate even to the point of host extinction, a so-called tragedy of the commons. Neither selection against transposons’ deleterious effects nor exploitation by parasitic elements is sufficient to prevent host extinction. Instead, our analysis shows that only active suppression by hosts avoids population collapse. In particular, suppression must act post-transcriptionally in order to prevent continuous escalation of the transposon–host genetic conflict. We argue that only with host enforcement of transposons can complex life exist.

## Abstract (web summary)

Transposons are genetic parasites that have had far-reaching effects on eukaryotic evolution. This modelling study shows that host defences against transposon activity are necessary to prevent species extinction.

## Introduction

Transposons—selfish genetic elements that duplicate themselves within host genomes—have had wide-ranging effects on eukaryotic evolution^1^. They originate vast tracts of DNA^2,3^, including the majority of the human genome; they are major sources of genetic variation, including half of all visible mutations in *Drosophila*^4^; they alter gene expression patterns, rewiring regulatory networks^5–7^; and they contribute to population divergence and hybrid incompatibility^8–10^. In human health, transposon activity has been implicated in oncogenesis^11^, neurological disorders^12^, hereditary diseases^13,14^, and ageing^15^. In response to transposons’ deleterious effects, eukaryotes have evolved complex counter-adaptations to suppress transposon proliferation^16–22^. Yet transposons remain enormously impactful, comprising the most abundant form of nucleic acid across sequenced genomes^23^.

Despite the importance of transposons for eukaryotic biology, there are major gaps in our understanding of their evolution. Seminal population-genetics models have demonstrated that transposon duplication, which increases the number of transposons within individuals, can be counteracted by natural selection against individuals carrying more transposons, which decreases transposon number across the population^24^. At first, this appears to offer an explanation for the long-term stability in transposon number that we observe empirically in any given population^25^. However, this balance requires that the transposon duplication rate itself remains stable over evolutionary time, which is inconsistent with transposon biology and cannot be explained by current theory^26^. Since the classic models were developed, there have been great leaps in our understanding of the molecular biology underlying the replication, suppression, and fitness effects of transposons^16–19,27–29^. While some of these mechanisms have been modelled^30–32^, others have not, and we lack a systematic understanding of how these crucial details influence the evolution of transposons.

Here, we develop new models of host–transposon co-evolution based on our current understanding of transposon biology. Our analyses reveal the importance of whether transposon families share their replication machinery, which we characterise as ‘public’ versus ‘private’ replication to reflect a key distinction made in the field of social evolution^33–36^. Under public replication—as exemplified by DNA transposons—transposons in the same family must pool their reproductive effort. This makes transposons self-limiting, because there is less incentive for individual transposons to invest in a shared resource. By contrast, under private replication—as exemplified by certain RNA transposons—our model predicts that natural selection pushes transposons to increase their duplication rate until their host population is driven to extinction, playing out a molecular ‘tragedy of the commons’^37,38^. We consider various processes that may prevent the tragedy, and conclude that the integrity of the eukaryotic genome—and ultimately complex life—rests on host suppression of transposon-mediated conflict, an example of enforced cooperation^39^.

## Results

**Fig. 1:**
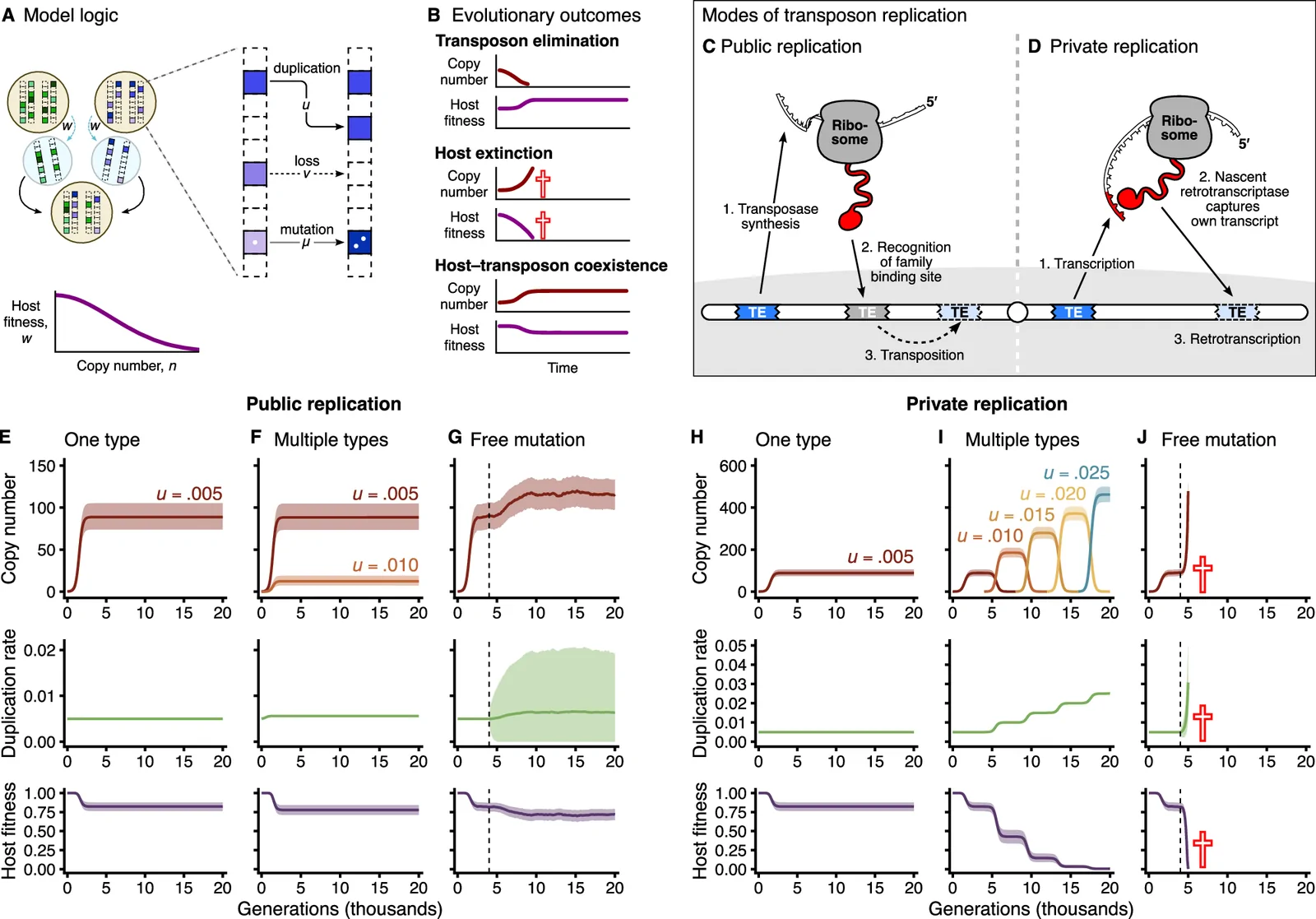
The tragedy of the transposons. Public versus private replication is a key feature of transposon biology that determines whether transposons will coexist with their hosts or drive their hosts extinct. **A**, **B** Model logic and potential evolutionary outcomes of the simulations; see main text for details. Red crosses indicate host extinction. **C**, **D** Public versus private replication. Under public replication, transposons cooperatively pool their resources so that each transposon’s investment into replication benefits all transpositionally competent transposons in the same family. Under private replication, each transposon’s investment into replication benefits that transposon only. **E**–**G** Under public replication, when the transposon duplication rate can freely evolve, there is selection for self-regulation of the duplication rate, such that transposons and hosts coexist. **H**–**J** Under private replication, when the transposon duplication rate can freely evolve, transposons are selected to be increasingly prolific. Selection for ever-increasing duplication rate among transposons drives the host population extinct in a tragedy of the commons. Panels **E**, **F**, **H** and **I** use the deterministic model; panels **G** and **J** use the stochastic model. Lines and ribbons in panels **E**–**J** show the mean and the 2.5% and 97.5% quantiles for copy number (across hosts), duplication rate (across transposons), and host fitness (across hosts). Dashed lines in panels **G** and **J** show the start of free duplication-rate mutation. Red crosses indicate host extinction. See “Methods—The tragedy of the transposons” for simulation details.

Our model builds upon the classic analysis of Charlesworth and Charlesworth^24^, and simulates how the distribution of transposons across the genomes of a diploid host population evolves from one generation to the next. Transposons duplicate at a rate *u* and are lost with probability *v* < *u* each generation, and a host’s reproductive success *w* is a decreasing function of its transposon number *n* (Fig. 1A). We use two implementations of this model for simulations. A deterministic, discrete population-genetics model is used for simulations involving up to two competing transposon types (i.e., wild-type and mutant), and allows us to neglect genetic drift by assuming an infinite host population. A stochastic individual-based model is used for simulations in which we allow transposon mutation rates to evolve freely, and allows us to consider an arbitrary number of competing transposon types by simulating a finite host population. Details of the two model implementations are given in “Methods—‘Deterministic population-genetics model’ and ‘Stochastic individual-based model’”.

In our analysis, there are three possible long-term outcomes: (i) selection eliminates all transposons from the host population; (ii) transposons proliferate until they drive the host population extinct; or (iii) transposon proliferation is balanced by selection against their deleterious effects, resulting in a stable mean copy number and coexistence between transposons and the host population (Fig. 1B).

### The tragedy of the transposons

An important assumption of classic transposon models is that the duplication rate does not freely evolve. These models either assume the duplication rate is fixed^24^; allow the duplication rate to evolve, but only downward^26,40–42^; or acknowledge that the duplication rate can increase, without analysing the ramifications^26,43^. Here, we allow the duplication rate to evolve freely, which we will show can be critical for evolutionary outcomes. Transposons harbour internal promoter sites^29,44,45^, the affinity of which to host transcription factors affects their expression level, and sequence variation between members of a transposon family correlates with their activity^46,47^. Thus, the duplication rate of a given transposon is affected by mutations to its sequence^48^. We first ask, therefore, how allowing for transposon evolution affects the predictions of the classic model. The answer depends upon the biological details of how replication happens for a particular transposon family.

We focus on two key modes of replication for transposons: what we call public versus private replication, based upon concepts in social evolution^33–36^. Under public replication, transposons pool their replicative effort with other transposons of the same family. This is an example of cooperation in social evolution, because individual transposons are each contributing to a trait that benefits all other transposons in the family^49^. Public replication is typified by DNA transposons, in which the encoded transposase protein moves back to the nucleus after translation, attaches to one of potentially many transposase binding sites in the genome, and transposes the associated sequence (Fig. 1C). By contrast, private replication mechanisms benefit an individual transposon only, with little or no sharing of replicative effort with other elements. This mode is typified by long interspersed nuclear elements (LINEs), a type of RNA transposon. LINEs encode a reverse transcriptase that, during protein synthesis, attaches to its own mRNA transcript to use it as a template for reverse transcription into the host DNA (Fig. 1D). This self-duplication property, known as *cis*-preference^50^, is mediated both by proximity between the nascent polypeptide and the RNA message while both are still attached to the ribosome^51^, and by recognition of particular sequences at the 3’ end of the transcript^52^. The other major group of RNA transposons, long terminal repeat (LTR) retrotransposons, does not exhibit this proximity-based mechanism and has been described as lacking *cis*-preference^27^. However, emerging lines of evidence suggest that several LTR lineages do exhibit *cis*-preference, such as *Ty1* in plants^53^, *Ty3* and *BEL*/*Pao* in vertebrates^54^, and *IAP* in mammals^55^, although others, such as *Ty1* in yeast^56^, do not. LTRs encode capsid proteins that associate with LTR mRNA in the cytoplasm and assemble into virus-like particles, within which the mRNA is reverse transcribed into cDNA to be transported into the nucleus and integrated into the host genome^57^. The mechanism for LTR *cis*-preference may be that only one LTR copy tends to be expressed in a cell at any given time, so that a given LTR does not usually have the opportunity to bind to another element’s RNA sequence^54^. Later in the paper, we consider a third mode of replication—parasitic replication—as typified by short interspersed nuclear elements (SINEs).

Our model studies the behaviour of a single family of transposons, in which each individual transposon has its own duplication rate *u*. Under public replication, *u* is the rate at which a given transposon replicates a randomly-selected transposon in its genome, such that the rate at which any specific transposon gets duplicated is equal to the average *u* across all transposons in the genome. Thus, each element receives an equal share of the total duplication effort of its family. When we run the deterministic model with all transposons exhibiting the same duplication rate *u*, which is not permitted to evolve, the result is a stable equilibrium in transposon abundance, as predicted by Charlesworth and Charlesworth^24^ (Fig. 1E; “Methods—The tragedy of the transposons”). Next, we introduce a mutant transposon into the deterministic model, which shows that transposons exhibiting different duplication rates can stably coexist under public replication (Fig. 1F). Finally, we allow duplication rates to evolve freely in our stochastic individual-based model, which leads to a similar pattern: transposons adopt a wide range of strategies, eventually settling on a stable distribution of duplication rates, and equilibrium is reached (Fig. 1G). In this scenario, a substantial fraction of elements contribute nothing to the family’s success. For example, in the simulation shown in Fig. 1G, 9% of the elements are completely inactive (*u* = 0) at equilibrium.

Private replication leads to a fundamentally different outcome. For these models, we assume that each transposon only replicates itself. Again, when we run the deterministic model with no duplication rate evolution, the same stable equilibrium is reached under private replication as was reached under public replication (Fig. 1H). However, when we introduce mutant transposons with a higher duplication rate, they competitively exclude transposons with a lower duplication rate, increasing the average transposon number and driving host fitness lower and lower (Fig. 1I). And when we allow transposons to freely evolve in our individual-based model, we find that they are selected to continually increase their duplication rate—with more-prolific transposons repeatedly outcompeting less-prolific transposons—until the host population goes extinct (Fig. 1J). Analogously to the classic tragedy of the commons^37,38,49^, the transposons prosper in exploiting a common resource—in this case, the host genome—even though this eventually results in its collapse and their own demise.

### Evolutionary logic

To determine why the mode of transposon replication (public versus private) leads to such fundamentally divergent outcomes, we conducted an invasion analysis of our deterministic model. This method allows us to derive a mathematical condition to explain when an increase in the transposon duplication rate is favoured by natural selection (see “Methods—Evolutionary logic”). A simplified version of the argument is provided here, while the full (and more rigorous) analysis is presented in Supplementary Methods 2.

Consider a population of hosts in which the average number of transposons per host genome is $\bar{n}$, and all transposons duplicate at rate *u* (for simplicity, we assume the loss probability *v* is 0). A transposon mutates to have a marginally higher duplication rate. Focus on an instance where, by virtue of its higher duplication rate, the mutant transposon (the “parent”) generates one new transposon (the “offspring”) that it would not have generated otherwise. If creating the offspring gives a net benefit to the parent, then we conclude that natural selection favours a small increase in the duplication rate. This argument can be recast in a social evolution framework using Hamilton’s rule^58^, which allows us to interpret the consequences of an increased duplication rate in terms of a transposon’s inclusive fitness. In this framing, natural selection favours an increase in the duplication rate when

1 $${rB}\, > C\,,$$

where *r* is the genetic relatedness between parent and offspring transposons, *B* is the number of new offspring that result from the higher duplication rate, and *C* is the cost to the parent of the higher duplication rate.

Under public replication, we have $r=1/\bar{n}\,$ because that is the probability that the parent duplicates itself and not a wild-type transposon sharing the same genome; under private replication, we have *r* = 1 because the parent necessarily duplicates itself. The benefit *B* is 1 under both public and private replication, because we are focusing on an instance where the parent creates one new transposon owing to its marginally higher duplication rate. And the cost *C* is the detriment to the parent transposon’s fitness that it causes by polluting its host’s genome with one additional transposon. If the host fitness function is *w*(*n*), then the host’s relative fitness can be written $W\left(n\right)=w(n)/\bar{w}$, where $\bar{w}$ is the average host fitness in the population. This relationship means that a host pays a fitness penalty d*W*/d*n* for every increase in its transposon number by one. At equilibrium, d*W*/d*n* = –*u*, because selection against the deleterious effects of transposons must counterbalance their proliferation rate^24^. This penalty is paid by the parent transposon for every generation that it stays associated with the offspring, which is two generations on average in a sexually-reproducing host species, because meiosis has a 50% probability of dissociating parent and offspring each generation. Accordingly, C=2u under both public and private replication.

Substituting these values for *r*, *B*, and *C* into condition (1) shows that natural selection favours an increase in the duplication rate *u* under public replication when

2 $$u < \frac{1}{2\bar{n}},$$

and favours a small increase to the duplication rate under private replication when

3 $$u < \frac{1}{2}.$$

**Fig. 2:**
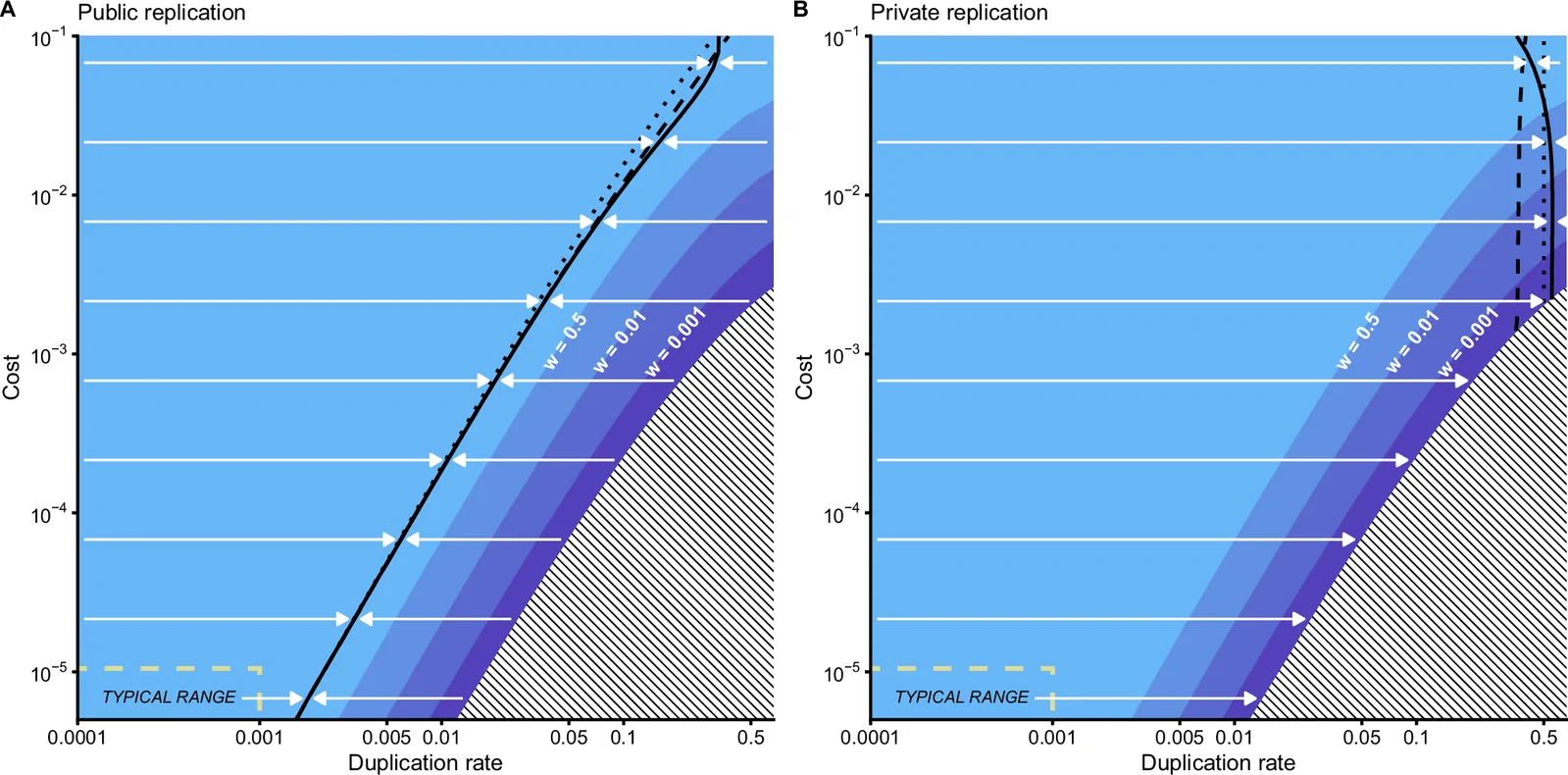
Evolution of the duplication rate under public versus private replication. These plots show the direction of evolution (arrows) of the duplication rate *u* (x-axis) for a given transposon fitness cost *c* (y-axis) and the fitness function $w\left(n\right)={\left(1-c\right)}^{{n}^{2}/2}$. **A** Under public replication, an intermediate duplication rate (solid black line) is selected for. **B** Under private replication, selection increases the duplication rate until the population is driven to extinction (cross-hatched area), except when fitness costs are extremely high, when an equilibrium for the duplication rate can be reached. The solid black line shows the evolutionarily stable strategy for *u* found by numerical solution of the deterministic model, the dashed line shows the analytical solution found using the invasion analysis (Supplementary Methods 2), and the dotted line shows the approximations of inequalities 2 and 3. See “Methods—Evolutionary logic” for details.

Under public replication, each transposon personally benefits from a fraction $1/\bar{n}$ of its reproductive effort, with the remainder of its effort being lost (from a fitness perspective) on making copies of unrelated transposons. Therefore, public transposons are subject to a “copy-number brake” (condition (2)) that makes increases to the duplication rate less beneficial as transposons become more abundant (Fig. 2A). Under private replication, however, each transposon enjoys the full benefit of its reproductive effort, there is no “copy-number brake” (condition (3)), and selection for faster duplication is much stronger (Fig. 2B).

The prediction for public transposons mirrors a classic result from social evolution that outlines how competition among replicating agents undermines the stability of a cooperative system, which in this case is the collective reproduction of transposons. When individuals are investing in a public good (here, transposons investing in shared reproduction), the costs are met by the individual, but the benefits are shared by all. This structure can mean that the most competitive strategies are ones that receive the benefits from the public good but invest little. In particular, as groups become larger, there is less and less incentive to contribute to a public good (i.e., the replication machinery) because the benefits are shared among more individuals^49^. As a publicly-replicating transposon family expands, therefore, the result is the evolution of a stable duplication rate that leads to a stable equilibrium for transposon copy number.

For private replication, it is also clear that sexual reproduction is the key to the tragedy of the transposons, because sexual recombination limits the scope for selection against more active transposons^26,59^. Specifically, a mutant transposon suffers the selective consequences of producing more descendant transposons only to the extent that it stays associated with them. From a transposon’s perspective, sex takes away half its genetic context each generation and replaces it with genetic material from its host’s mate (Supplementary Methods 2.6). This new genetic material may itself include a number of transposons, but this contribution is uncorrelated with the behaviour of the focal transposon. As a result, there is almost no scope for relatedness (in the social evolution sense of shared common ancestry^58^) between transposons to build up on the timescale relevant for duplications, with typical duplication rates being on the order of 10^–5^ to 10^–3^ per generation^27^. A more permanent association between a transposon and its relatives might provide them with an incentive to limit their replication, but regular recombination allows mutant prolific transposons to act with relative impunity, leveraging their transmissive advantage to displace wild-type transposons. Even if such an invasion lowers host fitness, this cost rapidly diffuses through the host population, such that the prolific transposons ultimately bear little penalty.

### How is tragedy avoided for private elements?

Privately-replicating transposons such as LINEs are extremely common, but clearly do not always drive their host populations extinct. How then do natural populations avoid transposon-driven extinction? We consider three potential mechanisms that each incorporate key additional features of transposon biology^27^: (i) stronger selection against transposition; (ii) parasitic elements exploiting transposon populations; and (iii) host suppression.

#### Costs of transposition

A tragedy of the commons arises for privately-replicating transposons because the benefits of a higher duplication rate within the genome exceed the costs arising at the between-genome level due to this greater activity. We therefore set out to determine whether this result was robust to different assumptions about the fitness cost of transposons. We considered two possibilities. First, whether we may have underestimated transposon-related fitness costs, and whether more stringent costs might discourage transposition. Second, whether there were fitness costs to the host associated with transposon activity itself, and that these might select more strongly against transposons increasing their duplication rate.

**Fig. 3:**
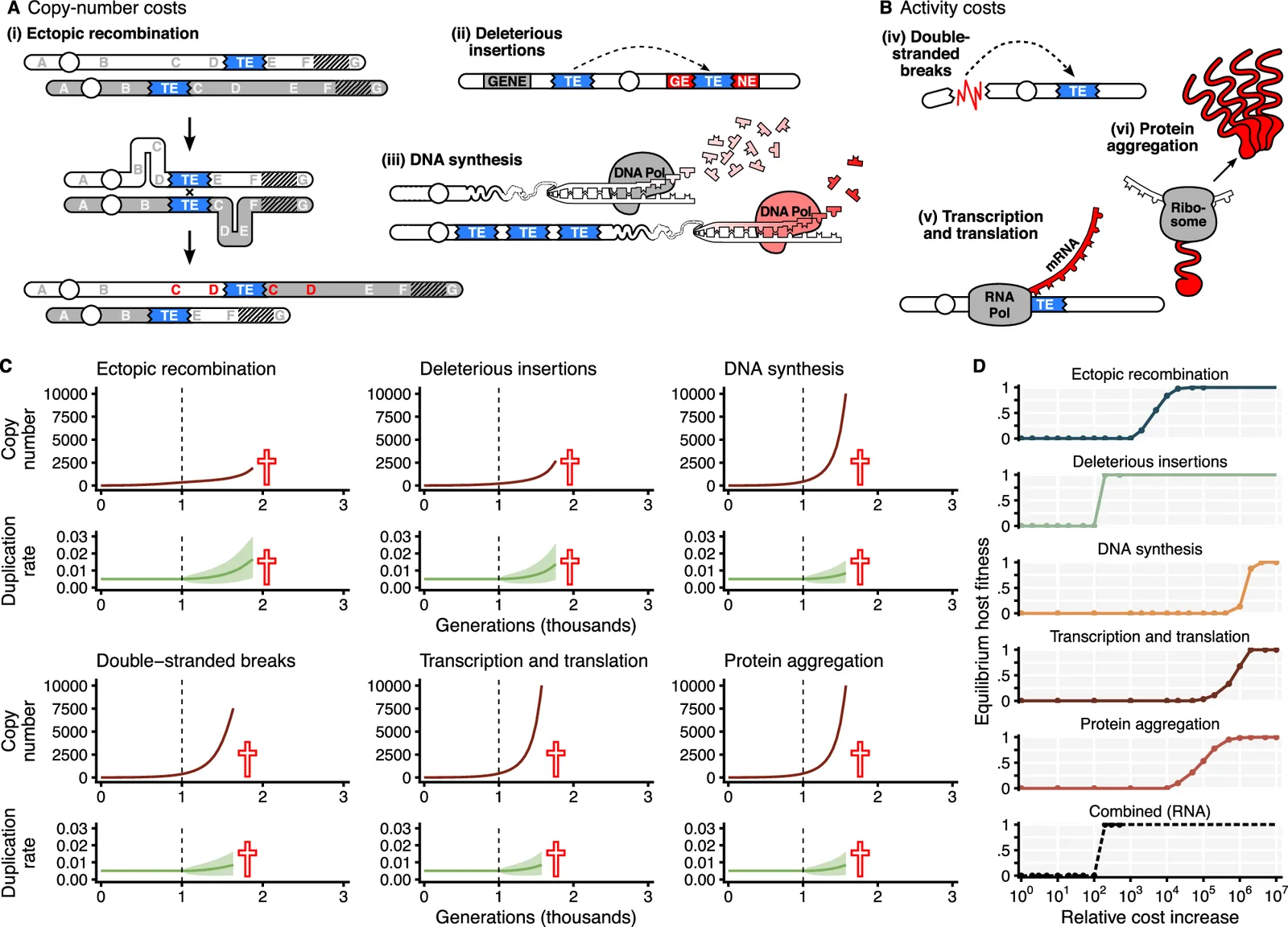
Fitness costs of transposons are not sufficient to prevent host extinction by private elements. **A**, **B** We consider six copy number and activity costs shown here; see main text for further details. **C** Regardless of which fitness cost we adopt, selection is insufficient to avoid host extinction. In each case, the individual-based model is run with empirically estimated costs; lines and ribbons show means and central 95% intervals. Red crosses indicate host extinction. **D** Fitness costs need to be strengthened by several orders of magnitude to prevent host extinction. The “relative cost increase” on the x-axis is the factor by which each cost is inflated relative to our best estimate based upon the literature. The “double-stranded breaks” cost is omitted here because it only applies to DNA transposons, which do not employ private replication. The final panel introduces a “combined” cost with all five fitness costs applicable to RNA transposons. Panel **D** shows the mean fitness across three runs of the stochastic model for each point; results were consistent across runs. See “Methods—Host fitness costs” for simulation details.

We identified six potential costs from the literature (Fig. 3A, B): (i) dispersed transposon sequences cause ectopic recombination^60,61^; (ii) insertion into coding sequences disrupts genes^24^; (iii) the proliferation of transposon sequences incurs a DNA synthesis penalty for the host^62,63^; (iv) unrepaired double-strand breaks are caused by “cut-and-paste” transposition of DNA transposons^41^; (v) the expression of transposon genes incurs an energetic cost for the host^64^; and (vi) massive overproduction of transposon gene products leads to protein aggregation, misfolding, and ribosomal stalling^65^. Costs i–iii are copy number costs, increasing with the number of insertions, but not with the duplication rate per se. Costs iv–vi are activity costs, increasing with the product of transposon number and average transposon duplication rate.

For each of the six fitness costs, we constructed a dedicated host fitness function parameterised with data collected from the literature (“Methods—Host fitness costs”). We also constructed a “combined” fitness function incorporating all five costs relevant for RNA transposons (i.e., excluding the cost of double-stranded breaks), since only RNA transposons use private replication. For each function, we assumed that the relative burden to the host of each additional transposon increases as more transposons accumulate (i.e., each function is log-concave with respect to transposon number). This relationship was established by Charlesworth and Charlesworth^24^ as a necessary condition for the transposon number to reach equilibrium, assuming a fixed duplication rate. We integrated these host fitness functions into our individual-based model, allowing the transposon duplication rate to evolve freely.

We find that none of the empirically supported cost functions we identified is strong enough to prevent population collapse (Fig. 3C). This outcome is partly because many of these costs are inherently weak. Indeed, we find that four of the six costs proposed in the literature (costs iii–vi) are too weak to meaningfully inhibit transposon proliferation, at least when acting on their own (Fig. S8). This result has likely gone unnoticed because previous studies only identified these as potential costs, but did not estimate the strength of selection that would be associated with them. The two remaining costs—ectopic recombination (cost i) and deleterious insertions (cost ii)—could potentially keep transposon number in check if the duplication rate were fixed. But none of the six costs is strong enough to prevent host extinction when the duplication rate is permitted to evolve, even if we inflate our cost estimates by several orders of magnitude (Fig. 3D). Allowing costs to vary by site also did not change this finding (Fig. S9). Under the most stringent cost model—i.e., the “combined” model incorporating the five fitness costs relevant for RNA transposons—we have to increase costs 200-fold relative to the most plausible estimates to prevent a tragedy of the commons in privately-replicating transposons. This contrasts strongly with publicly-replicating transposons, in which a realistic cost model for DNA transposons easily allows the survival of the host population (Fig. S9).

We therefore conclude that natural selection against the costs associated with transposition is not, in itself, sufficient to prevent the likely extinction of host populations.

#### Parasitic elements

**Fig. 4:**
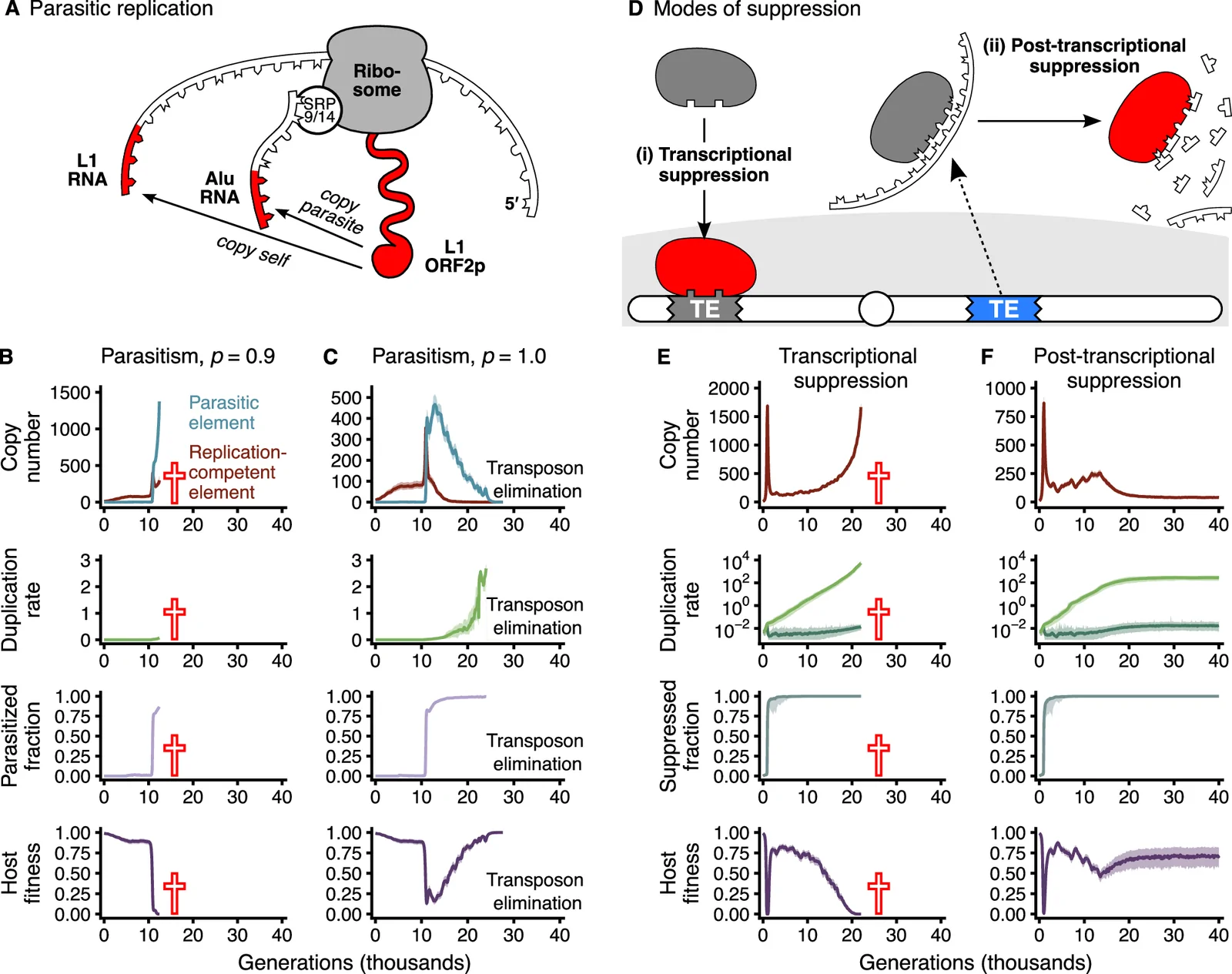
Host suppression, but not parasitism, can prevent host extinction via private elements. **A** Parasitic replication: LINE elements, such as *L1* in mammals, are vulnerable to parasitism by non-autonomous SINE elements, such as *Alu* in primates. **B** Introducing a parasitic element which cannot completely exploit its target element (*p* = 0.9) does not avert the tragedy of the transposons. Red crosses indicate host extinction. **C** When the parasite is permitted to completely exploit its target element (*p* = 1.0), it eliminates that element completely, and then dies off itself. **D** Mechanisms of host suppression, contrasting transcriptional and post-transcriptional suppression; see main text for details. **E** With transcriptional suppression, an arms race is instituted whereby transposons compensate for suppression by increasing their activity. Eventually, the cost of maintaining suppression overwhelms the host, leading to host extinction. In the duplication rate panel, the lighter line shows what the duplication rate would be in the absence of host suppression, while the darker line shows the duplication rate accounting for suppression. Red crosses indicate host extinction. **F** Post-transcriptional suppression, which targets gene products such as RNA, has the effect of increasing a transposon’s cost of replication. Increasing costs can lead to a stable equilibrium for the transposon duplication rate. All results are from the individual-based model; lines and ribbons show means and central 95% intervals. See “Methods—‘Parasitic replication’ and ‘Host suppression’” for simulation details.

Privately-replicating transposons can themselves be parasitized by sequences that interfere with *cis*-preference^27^. This interaction is observed between LINEs and SINEs. For example, the mammalian *L1* retrotransposon (a LINE) is parasitized by the *Alu* element (a SINE). *Alu*s encode no replication machinery of their own. Instead, the *Alu* mRNA binds to the host signal recognition particle protein SRP9/14, attaches to the ribosome, and competes with the *L1* mRNA for binding with the nascent *L1* ORF2p retrotranscriptase^66,67^ (Fig. 4A). We would not expect parasitism to be a general solution for preventing host extinction, because most privately-replicating transposon families lack parasitic elements. Nonetheless, we wondered whether parasitic elements could be responsible, in some cases, for creating a stable transposition rate.

We therefore incorporated parasitism into our individual-based model (“Methods—Parasitic replication”). Using the “combined” RNA transposon fitness model from the previous section, we ran simulations with one privately-replicating transposon family and one parasitic family. We assume that when both the autonomous and the parasitic element are associated with the ribosome, the retrotranscriptase binds to the parasite with probability *p* (and to the autonomous element otherwise), such that *p* captures the efficiency of parasitism.

We found that only highly effective parasites can collapse the transposon population and prevent host extinction. Specifically, extinction is only prevented if the elongating reverse transcriptase binds to the parasitic RNA rather than its own RNA more than 95% of the time when presented with both (Figs. 4B, C, S10). This seems unlikely to be satisfied. While *Alu* elements are common in the human genome, only about 50% of new inserts by *L1* machinery are *Alu* elements^27^, which suggests that *Alu* elements are unable to monopolise the *L1* machinery to the extent that is needed to suppress transposition in our model. More generally, parasitized elements would be expected to evolve an increased self-specificity in response to parasitism. Therefore, parasitism of private transposons seems insufficient to avert host extinction.

#### Host suppression

Finally, we investigated the potential for the evolution of host suppression that puts a limit on the activity of transposons. We considered two potential modes of host suppression, transcriptional versus post-transcriptional suppression (Fig. 4D). With transcriptional suppression, transcription is “turned off” at the source, and suppression dampens both the transposon’s duplication rate and its expenditure of cellular resources (i.e., due to transcription and translation of the transposon). With post-transcriptional suppression, transcripts are degraded after they are produced, and hence suppression dampens the transposon’s duplication rate, but the transposon still spends as much host energy transcribing itself; post-transcriptional suppression acting on transposon proteins would have a similar effect.

Both modes of suppression are observed empirically. For example, the Piwi-piRNA pathway in animals^16–19^ performs sequence-specific silencing of transposons through both transcriptional suppression (e.g., by DNA methylation or histone modification) and post-transcriptional suppression (by degradation of mRNA). Other RNA interference pathways in plants and fungi similarly perform both transcriptional and post-transcriptional transposon suppression^68^. Further lineage-specific mechanisms for controlling particular transposon families include KRAB zinc-finger genes in primates that transcriptionally suppress transposon DNA^20^; or TEX19.1 in mammals, which post-transcriptionally suppresses *L1* elements by degrading their ORF1p proteins in pluripotent stem cells during early development^21^.

To model transcriptional and post-transcriptional suppression, we introduce into our individual-based model a host suppression locus encoding a continuous trait *S* ≥ 0, where each unitary increase in *S* causes a decrease in transposon activity and an increase in the cost of host suppression (“Methods—Host suppression”). The evolutionary outcome depends upon the mode of host suppression. With transcriptional suppression, the result is an ever-escalating evolutionary arms race. In response to increased suppression, transposons increase their activity even more, until the cost of maintaining suppression itself grows too severe and populations are driven extinct (Figs. 4E, S11).

In contrast, with post-transcriptional suppression, a stable equilibrium is eventually reached—that is, the arms race reaches a point at which neither party is selected to further escalate the conflict (Figs. 4F, S11). This outcome occurs because, unlike suppression that prevents transcription, suppression that degrades transposon gene products makes replication increasingly energetically costly, as transposons need to generate more and more mRNA and/or protein for each transposition event. As this direct cost on the transposons increases, it eventually makes further investment in replication by the transposons net costly, setting the upper limit to their duplication rate.

We showed earlier that the fitness costs associated with transcription, translation, and protein aggregation (costs v and vi, Fig. 3B) were too weak to meaningfully restrict transposon proliferation when acting on their own (Fig. S8). When there is a high level of post-transcriptional suppression, these transposon activity costs become substantially stronger, because suppression vastly increases how much mRNA and protein transposons need to produce per successful duplication. As a result, in the presence of suppression, natural selection can favour self-restraint in the sense that further increases in transposon replication are selected against.

While post-transcriptional suppression is required for stable host-transposon coexistence, either form of suppression improves host fitness, and so we would predict both forms of suppression to be present in natural populations. The presence of an RNA interference pathway in the most recent common ancestor of all eukaryotes^68,69^ supports our prediction that post-transcriptional suppression is critically important to the long-term survival of sexually reproducing populations.

## Discussion

Transposons are central to the evolution of life but come in a wide variety of forms. To better understand their evolutionary dynamics, we have argued that it is useful to divide transposons into three types—public, private, and parasitic—each of which adopts different replication strategies within the genome. Public transposons can be self-limiting, but private transposons are selected to continually increase their duplication rate. In the absence of enforcement by the host, this is predicted to lead to a tragedy of the commons that results in the death of the hosts and the transposons with them.

Is there evidence that our distinction between public and private replication is important for how transposons evolve? Social evolution theory predicts that genes encoding private traits should be subject to stronger purifying selection than genes encoding public traits^70^. This prediction arises because the fitness costs associated with loss-of-function mutations can be partially masked by other group members for public traits. Moreover, a dedicated test comparing private and public traits in bacteria found evidence for stronger purifying selection in private traits^36^. Similar patterns are seen in transposon evolution. An analysis of the molecular evolution of transposons across 307 vertebrate genomes found that RNA transposons with *cis*-preference were under much stronger purifying selection than DNA transposons^54^. It was further suggested that this purifying selection allowed RNA transposons to persist better. This supports our prediction that privately-replicating transposons are a greater threat to hosts than publicly-replicating transposons, as the latter allow poorly-functioning transposons to persist and are therefore subject to weaker purifying selection. Since sexual recombination facilitates increases to the duplication rate in our model, further empirical and theoretical study of public versus private transposon replication in hosts with regular selfing^8^ would be illuminating.

We have treated public versus private replication as discrete strategies for transposon replication, which is motivated by the molecular biology of replication for DNA versus LINE transposons in particular. However, evidence of private replication in LTR transposons is less clear-cut^27,53–56^. It is possible that LTR transposons may exhibit intermediate levels of “privacy” in replication, and future work could explore the resulting evolutionary dynamics. A prediction of our model is that hosts are selected to suppress privately-replicating transposons more highly than publicly-replicating transposons (Figs. S11, S12). In this case, hosts may apply an intermediate level of suppression to LTR transposons.

In the face of the threat from transposons, our models predict that hosts will evolve to suppress transposons both pre- and post-transcriptionally. However, we find that post-transcriptional suppression is required for a stable equilibrium to be reached. Our models also predict co-evolutionary escalation. This prediction is clearest for privately-replicating RNA transposons, which threaten their hosts with extinction. However, there is also the potential for some escalation with publicly-replicating DNA transposons whenever they evolve to be sufficiently costly for a host so as to favour suppression mechanisms (Fig. S12). Empirical evidence of such a transposon–host arms race is observed in the ZNF93 transposon suppressor gene in primates. Here, privately-replicating *L1* transposons are suppressed by the ZNF93 protein binding to *L1* DNA, which prevents transcription. Some *L1* elements have escaped suppression via an internal deletion of the ZNF93 binding sequence, though this comes at the cost of a reduced expression level for the transposon^20^.

An associated prediction of our model is that conflict between hosts and transposons will lead to a high level of transposon expression and a high level of suppression, resulting overall in low duplication rates. Here, a potential criticism of the model is that it assumes that both host and parasite can continually escalate their arms race. Clearly, biochemical constraints will eventually prevent transposons from adopting arbitrarily high duplication rates, and hosts from suppressing transposition with complete precision. Nonetheless, empirical findings support that transposons can be very highly transcribed. Even with suppression in place, a significant proportion of transcribed RNA in mammalian germlines—e.g., 3% in mice, and 15% in humans—originates from transposons^71^. These observations are consistent with our modelling assumption that the host cannot completely suppress transposon activity. Furthermore, the strength of suppression appears to be substantial, especially through the primary eukaryotic line of defence against transposons, the Piwi-piRNA pathway. When this pathway is knocked out in mice, for example, spermatogenesis becomes impossible owing to extreme proliferation of transposons in germline cells^72^. Further, when PIWI activity is knocked down in *Drosophila*, measured abundances of transposon RNA in the ovary increase up to 30-fold^73^. Perhaps the most famous transposon–host conflict is in *Drosophila* offspring inheriting *P* elements from their father, but no *P*-suppressing piRNAs from their mother, causing *P-M* hybrid dysgenesis^32^.

Our work suggests that the ways that transposons interact during replication, which can be both cooperative and competitive, have been fundamental to their evolution. More generally, our study demonstrates the importance of viewing transposon evolutionary dynamics not merely as a balance between opposing, static forces of proliferation and selection, but also as a fundamentally adaptive process. Transposons evolve as selfish genetic elements adapting to their hosts, and hosts in turn adapt to the presence of their selfish elements^27,74^. Mirroring many other examples of social evolution^39,75^, our work also suggests that it is only through mechanisms of enforcement—in this case suppression of transposon activity—that this genetic conflict can be kept in check, making the evolution of complex life possible.

## Methods

### Basic models

#### Deterministic population-genetics model

The deterministic model with two transposon types assumes an infinitely large, diploid, sexually-reproducing, hermaphroditic host population with non-overlapping generations. The host population carries transposons of two types, type 1 and type 2. The two transposon types differ in duplication rate, *u*_1_ and *u*_2_, but have the same loss (or excision) probability *v*. The frequency of hosts with *n* type-1 and *m* type-2 transposons is ${f}_{n,m}$, such that ${\sum }_{n,m}{f}_{n,m}=1$.

Each generation, the population goes through three phases. In the proliferation phase, genomes are updated with respect to transposon duplication and loss. The probability of *k* duplications among *n* transposons with duplication rate *u* is ${\mathbb{P}}({k|nu})$, where ${\mathbb{P}}\left(k|\lambda \right)={\lambda }^{k}{e}^{-\lambda }/k!$ is the Poisson probability mass function, and the probability of *k* excisions among *n* transposons with excision probability *v* is ${\mathbb{B}}({k|n},v)$, where ${\mathbb{B}}\left({k|n},p\right)=\left({{n}\atop{k}}\right) {p}^{k}{\left(1-p\right)}^{n-k}$ is the binomial probability mass function. The duplication rate *u* is equal to *u*_1_ for privately-replicating transposons of type 1, *u*_2_ for privately-replicating transposons of type 2, and (*nu*_1_ + m*u*_2_) / (*n* + *m*) for publicly-replicating transposons in a genome with *n* type-1 transposons and *m* type-2 transposons. The frequency of hosts with *n* type-1 and *m* type-2 transposons after the proliferation phase is therefore

4 $$	{F}_{n,m}={\sum }_{i=0}^{n}{\sum }_{j=0}^{m}{\sum }_{k=n-i}^{n}{\sum }_{{\ell}=m-j}^{m}{f}_{i,j}{\mathbb{P}}\left(k|i{U}_{1}\right){\mathbb{B}}\left(i-n+k|i,v\right) \\ 	{\mathbb{P}}\left({\ell}|j{U}_{2}\right){\mathbb{B}}\left(j-m\,+\,{\ell}|j,v\right).$$

In the selection phase, each host survives to reproductive age with probability proportional to its fitness *w*(*n*, *m*) and otherwise dies. The frequency of hosts after the selection phase is therefore

5 $${\phi }_{n,m}=\frac{{F}_{n,m}w\left(n,m\right)}{{\sum }_{i,j}{F}_{i,j}w\left(i,j\right)}.$$

Finally, in the recombination phase, each host produces a large number of gametes. No linkage is assumed between transposons for the deterministic model. The frequency of gametes with *k* type-1 transposons and ${\ell}$ type-2 transposons is therefore ${{g}}_{k,{\ell}}={\sum }_{n,m}{\phi }_{n,m}{\mathbb{B}}({k|n},\frac{1}{2}){\mathbb{B}}\left({\ell}|m,\frac{1}{2}\right)$, where ${\phi }_{n,m}$ is the frequency of genotype *n,m* hosts after the selection phase. Finally, gametes fuse to form the next offspring generation,

6 $${f}_{n,m}^{{\prime} }={\sum }_{k=0}^{n}{\sum }_{{\ell}=0}^{m}{g}_{k,{\ell}}{g}_{n-k,m\,-\,{\ell}},$$

replacing the parental generation and returning the population to the beginning of the life cycle. There is also a one-type deterministic model which can be recovered from the two-type model in Eqs. (4–6) by setting *m* = 0 for all individuals. Across all deterministic simulations, we set *u*_1_ = 0.005 and *v* = 0.0005 for similarity with the model of Charlesworth and Charlesworth^24^. Full details on the deterministic models can be found in Supplementary Methods 1.1–1.2.

#### Stochastic individual-based model

There are *K* hosts, each with 2*H* linear chromosomes, where each chromosome comprises *L* sites. Sites can be empty or occupied by a transposon. Indexing an individual *i*’s *n*_*i*_ transposons by *j* = $\left\{1,\,2,\ldots,{n}_{i}\right\}$, we denote the duplication rate of the *i*th individual’s *j*th transposon by *u*_*i,j*_. At simulation start, all transposons have a duplication rate *u*_0_. Every generation, each genome is updated by ${k}_{i}^{{{{\rm{d}}}}}$ duplications, ${k}_{i}^{{{{\rm{e}}}}}$ excisions, and ${k}_{i}^{{{{\rm{m}}}}}$ mutations, where ${k}_{i}^{{{{\rm{d}}}}}$ is drawn from a Poisson distribution with mean *A*_*i*_ = ∑_*j*_ *u*_*i,j*_, ${k}_{i}^{{{{\rm{e}}}}}$ is drawn from a binomial distribution with *n*_*i*_ trials each with success probability *v*, and ${k}_{i}^{{{{\rm{m}}}}}$ is drawn from a binomial distribution with *n*_*i*_ trials each with success probability µ_u_. Then: ${k}_{i}^{{{{\rm{d}}}}}$ elements are randomly drawn with replacement, where the probability of element *j* being drawn is *u*_*i,j*_ / *A*_*i*_ for private elements and 1/*n*_*i*_ for public elements, and properties of these elements are saved for later insertion; ${k}_{i}^{{{{\rm{m}}}}}$ elements are selected, with replacement, and their duplication rate *u*_*i,j*_ is changed to *u*’_*i,j*_ = max(0, *u*_*i,j*_ + δ*u*), where δ*u* is drawn from a normal distribution with mean 0 and standard deviation *σ*_u_ + *τ*_u_ *u*_*i,j*_ (that is, there is both an absolute component *σ*_u_ and a relative component *τ*_u_ of transposon mutation); ${k}_{i}^{{{{\rm{e}}}}}$ elements are selected, without replacement, and replaced by empty sites; and then the elements previously selected for duplication are inserted into randomly-selected empty sites.

Individuals reproduce with probability proportional to their fitness *w,* and gametes are generated via meiosis of parental chromosomes, where the number of chiasmata per chromosome is drawn from a Poisson distribution with mean *χ*. Gametes fuse to form *K* new individuals, returning the population to the beginning of the life cycle. The population is assumed extinct when the mean fitness drops below 10^–6^. Across all stochastic simulations, we set *u*_0_ = 0.005, *v* = 0.0005, *H* = 2, and *χ* = 0.9 for similarity with the model of Charlesworth and Charlesworth^24^; for mutation rates, we set *μ*_u_ = 0.01, *σ*_u_ = 0.001, and *τ*_u_ = 0.05. Full details on the stochastic models can be found in Supplementary Methods 1.3.

### Simulation details

#### The tragedy of the transposons

For the simulations demonstrating the tragedy of the transposons (Fig. 1), we used the deterministic model for simulations with up to two transposon types competing at a time (Fig. 1E, F, H, I) and the stochastic model with population size *K* = 50,000 for the simulations with free mutation of the duplication rate (Fig. 1G, J). For the host fitness function, we used the ectopic recombination fitness model (see section 2.3) with $\lambda \varepsilon \rho /100=5\times {10}^{-5}.$ Note that in Fig. 1I, we show competition among five transposon types by successively replacing the outcompeted transposon type with a new, more competitive type.

#### Evolutionary logic

We conducted an invasion analysis of our deterministic model to derive a mathematical condition that governs whether an increase to the transposon duplication rate is favoured or disfavoured by natural selection. (While some authors restrict the term “natural selection” to forces acting at the level of the individual organism, we consider natural selection more broadly as operating on any entities that display heritable variation in fitness^76^). The informal derivation of conditions (2) and (3) in the Results makes three key simplifying assumptions, namely that (i) variation in the transposon number *n* among genomes is negligible; (ii) the transposon duplication rate *u* is small; and (iii) natural selection at the host level acts slowly compared to the effect of meiosis in breaking up associations between transposons. We relax these assumptions in Supplementary Methods 2, where the full invasion analysis is presented. This more robust analysis recovers conditions (2) and (3) of the Results in the limit of large $\bar{n}$ (which decreases the relative variability between hosts in transposon number) and small *u* (which in turn implies relatively weak selection at the host level).

For the numerical simulations illustrating evolutionarily stable strategies for the transposon duplication rate (Fig. 2), we first used the deterministic model with one transposon type to map out a “fitness surface” by running the model to equilibrium across a grid of different values for the fitness cost *c* and duplication rate *u*; the equilibrium host fitness $\bar{w}$ furnishes the shaded background areas for Fig. 2. Then, across a grid of values for the fitness cost *c*, we repeatedly chose values of *u* and ran the deterministic model with two transposon types to determine whether a mutant transposon with duplication rate *u* + 0.0001 could invade. We used a binary search to find, for each value of *c*, the value of *u* at which a faster-duplicating mutant was no longer favoured to invade. This is the equilibrium level of *u* found through numerical simulation, which is plotted in Fig. 2 as a solid line. The dashed line is the equilibrium *u* identified by the full invasion analysis, while the dotted line is the equilibrium *u* identified by the simplified analysis in the main text.

#### Host fitness costs

For the simulations exploring different host fitness functions (Figs. 3, S8), we used the stochastic model with population size *K* = 5000. We derived six alternative host fitness functions based on potential fitness costs associated with transposons and their activity. These are presented in Eqs. (7–12) below; more extensive details and derivations may be found in Supplementary Methods 3.

The ectopic recombination fitness function is

7 $$w={\left(1-\frac{\lambda \varepsilon \rho }{100}\right)}^{\frac{1}{2}n\left(n-1\right)-{n}_{g}}$$

where λ=0.005 is the length of the transposon in Mb, ε=0.15 is the efficiency of ectopic recombination relative to allelic recombination, ρ=1 is the allelic rate of recombination in cM/Mb, *n* is transposon copy number, and ${n}_{g}$ is the number of transposon copies that are homozygous (${n}_{g}=0$ in the infinite-population model).

The deleterious insertions fitness function is

8 $$w={\left(1-s\right)}^{\frac{{n}_{g}}{2}}{\left(1-{Hs}\right)}^{{n}_{h}}{\left(1-{ts}\right)}^{\frac{1}{8}{n}_{g}\left({n}_{g}-2\right)}{\left(1-{Hts}\right)}^{\frac{1}{2}{n}_{g}{n}_{h}}{\left(1-{H}^{2}{ts}\right)}^{\frac{1}{2}{n}_{h}\left({n}_{h}-1\right)}$$

where ${n}_{h}$ is the number of heterozygous insertions, *s* = 0.01 is the average effect of a homozygous insertion, *H* = 0.1 is the relative effect of a heterozygous insertion compared to a homozygous insertion; and *t* = 0.025 is the relative epistatic effect for each pair of homozygous insertions.

The DNA synthesis fitness function is

9 w=1−Yλn

where *Y* = 1 × 10^–5^ is the cost of synthesising 1 Mb of DNA.

The double-stranded breaks fitness function is

10 $$w=\exp (-{Bj}\bar{u}n-C{\left(j\bar{u}n\right)}^{2})$$

where *j* = 10 is the number of “cut-and-paste” transpositions per successful copy, $\bar{u}n$ is the total duplication rate of transposons in the genome, and *B* = 2.32 × 10^−3^ and *C* = 3.12 × 10^−5^ are fitted constants. Here, $j\bar{u}n$ represents the rate of formation of double-stranded breaks.

The transcription and translation fitness function is

11 $$w=1-{kc}\bar{u}n$$

where *k* = 10 is the number of assembled particles required for one successful transposition in the absence of host suppression and *c* = 6 × 10^–8^ is the energetic cost per assembled transposon particle.

The protein aggregation fitness function is

12 $$w=1-M{\left(\omega k\bar{u}n\right)}^{2}$$

where *M* = 7.19 is a fitted constant and ω = 1.4 × 10^–7^ is the mass of a single transposon particle relative to the total mass of protein produced by the cell.

For the “combined” fitness function for DNA transposons, we multiplied together the right-hand sides of all fitness functions above. For the “combined” function for RNA transposons, we did the same but omitted the double-stranded breaks function.

#### Parasitic replication

For simulations of parasitic elements (Figs. 4A–C, S10), we use the stochastic individual-based model detailed above with some modifications. We assume that the binding of parasites to ribosomes follows Michaelis-Menten dynamics, and hence the proportion of all duplication events in a host that are parasitized is given by $p{A}^{{\prime} }/({h}_{{{{\rm{P}}}}}+{A}^{{\prime} })$, where ${A}^{{\prime} }=\sum {u}^{{\prime} }$ is the total duplication rate of the parasitic elements, *h*_P_ is a half-saturation constant, and *p* is the saturating probability of parasitism. Here, ${A}^{{\prime} }/({h}_{{{{\rm{P}}}}}+{A}^{{\prime} })$ can be interpreted as the probability that a parasitic element is bound to the ribosome during elongation of a retrotranscriptase, and accordingly *p* can be interpreted as the probability that the parasitic element, and not the autonomous privately-replicating element, is bound by the reverse transcriptase when it is presented with both the parasitic mRNA and its own mRNA. Accordingly, the effective duplication rate of each privately-replicating element is $U=u\left(1-p{A}^{{\prime} }/({h}_{{{{\rm{P}}}}}+{A}^{{\prime} })\right)$. Similarly, the effective duplication rate of each parasitic element is ${U}^{{\prime} }=({u}^{{\prime} }/{A}^{{\prime} })\left({Ap}{A}^{{\prime} }/({h}_{{{{\rm{P}}}}}+{A}^{{\prime} })\right)={Ap}{u}^{{\prime} }/({h}_{{{{\rm{P}}}}}+{A}^{{\prime} })$, where *A* is the sum of all privately-replicating elements’ duplication rates *u*. Across all simulations with parasitism, we set *K* = 5000 and *h*_P_ = 100.

#### Host suppression

For the simulations exploring host suppression (Figs. 4D–F, S11, S12), each individual carries two suppressor alleles, *s*_1_ and *s*_2_, each of which is randomly mutated with probability *µ*_s_ to a new value *s*’ = max(0, *s* + δ*s*), where δ*s* is drawn from a normal distribution with mean 0 and standard deviation *σ*_s_ + *τ*_s_ *s*. Suppressor loci are unlinked to transposon loci and to each other, and one of each pair of suppressor alleles is passed on randomly to each gamete. We assume that suppressor dynamics follow Michaelis-Menten dynamics, such that when the amount of suppression expressed by a host is *S* = *s*_1_ + *s*_2_, the effective duplication rate for a given transposon is multiplied by a factor *h*_S_ / (*h*_S_ + *S*), where *h*_S_ is a half-saturation constant. Across all simulations with host suppression, we set *K* = 10,000, *μ*_s_ = 0.025, *σ*_s_ = 0.5, *τ*_s_ = 0.25, and *h*_S_ = 10.

The cost of host suppression is difficult to determine. We assign a fitness cost of 10^–7^ for each unit of suppression, which is enough to select against needless suppression without preventing hosts from adequately suppressing transposons. This assumes that transcriptional and post-transcriptional suppression are equally costly, which may not be the case. However, we conduct a sensitivity analysis of the cost of transcriptional and post-transcriptional suppression in Fig. S11, showing that results are similar across orders of magnitude for the fitness cost.

## Supplementary information

Supplementary Information

Transparent Peer Review file

## Data Availability

All code and data available at https://www.github.com/nicholasdavies/transposons^77^.

## Code availability

All code and data available at https://www.github.com/nicholasdavies/transposons^77^.
