# Supplementary material for: Cooperation, competition and enforcement in transposon evolution
Source: Nat Commun. 2026 Aug 12;17:9689. doi: 10.1038/s41467-026-75312-1 (PMC13558583; doi:10.1038/s41467-026-75312-1)
Supplement: Supplementary file 1 [file 41467_2026_75312_MOESM1_ESM.pdf]

Host suppression prevents a molecular tragedy of the commons in transposon evolution *Supplementary Methods*

N. G. Davies, J. A. Ågren and K. R. Foster

Contents

1 Models of transposon-host coevolution **2**
1.1 Deterministic one-type model . . . 2
1.1.1 Equilibrium copy number . . . 5
1.2 Deterministic two-type model . . . 7
1.3 Stochastic individual-based model . . . 8
1.3.1 Host suppression . . . 9
1.3.2 Parasitic transposon replication . . . 9
1.4 Parameter assumptions . . . 9

2 Invasion analysis **11**
2.1 Details of the approach . . . 11
2.2 Distribution of wild-type and mutant transposons at equilibrium . . . 14
2.3 Experienced relative fitness and transposon number at equilibrium . . . 15
2.4 Benefit: Initial difference ΔN_1 for the offspring transposon . . . 18
2.5 Cost: Initial difference ΔN_1 for the parent transposon . . . 22
2.6 Calculating subsequent differences . . . 23
2.7 Accuracy of approximations . . . 25
2.8 Putting it all together . . . 28

3 Host fitness models **31**
3.1 Ectopic recombination . . . 31
3.2 Deleterious insertions . . . 32
3.3 DNA synthesis . . . 34
3.4 Double-stranded breaks . . . 34
3.5 Transcription and translation . . . 35
3.6 Protein aggregation . . . 37

4 Additional figures **39**

1 Models of transposon-host coevolution

Here, we describe in detail the mathematical models we use to simulate transposon-host coevolution.

All models analyse a population of diploid, sexually-reproducing, hermaphroditic hosts with non-overlapping generations. Each host carries some number of transposons which we denote by n, the transposon copy number. Each transposon has a per-generation duplication rate u and a per-generation loss (or excision) probability v. In the absence of host suppression, the duplication rate of a transposon is equal to the mean number of copies that transposon makes per generation—either copies of itself or copies of other transposons. All transposons are assumed to belong to the same transposon 'family', but depending on the model, there may be multiple transposon types which differ in duplication rate u. Host fitness w is a function of the host's transposon number n and/or the total transposon activity $A = \sum u$, where the sum is taken over all transposons carried by the host. See Table 1 for a glossary of mathematical notation used throughout this document.

There are three models described here. Two are deterministic models, which assume an infinite population size and no linkage disequilibrium: the **deterministic one-type model** is used to model dynamics when there is one type of transposon, and the **deterministic two-type model** is used to model competition between two types of transposons. The third model is the **stochastic individual-based model**, which assumes a finite population with linkage disequilibrium and is used to model competition between arbitrarily many transposon types as well as the evolution of host suppression.

The predictions of the different models line up for test cases with overlapping assumptions, as shown in Fig. 1 of the main text. We use the deterministic models as a starting point in the main text for describing and analysing transposon–host coevolution. The simpler mathematics of the deterministic models also allows us to derive an 'invasion analysis' to explain when natural selection favours the evolution of an increased transposon duplication rate (see section 2). We use the more complex stochastic individual-based model to verify the results of the invasion analysis and to simulate transposon–host coevolution without having to make the simplifying assumptions necessitated by the deterministic models.

1.1 Deterministic one-type model

In the deterministic one-type model, there is an infinite population of diploid, sexually-reproducing, hermaphroditic hosts with non-overlapping generations. Each host is born carrying some number of transposons in its genome. The frequency of hosts carrying $n \in \{0, 1, 2, ...\}$ transposons at birth is f_n, where the frequencies are normalised such that $\sum_n f_n = 1$. This generation of hosts passes through three phases (Fig. S1) to produce the subsequent generation of hosts with frequencies f'_n.

First, in the **proliferation** phase (Fig. S1A), transposons undergo both duplication and excision. Specifically, each transposon generates $I \sim \text{Poisson}(\lambda = u)$ copies and has a probability v of being excised, where u is the transposon's duplication rate and v is the excision probability. (We assume that newly-generated transposon copies are not at risk of excision, or that this risk of excision for copies is incorporated into the duplication rate.)

Table 1: Table of notation

Symbol	Meaning
a_n	Distribution of wild-type transposons
A	Total activity of transposons
A'	Total activity of parasitic transposons
b_m	Distribution of mutant transposons
B	Benefit of duplication event
$\mathbb{B}(x\|n,p)$	Binomial probability mass function
β	Short form for $\beta_{wn}/\bar{w}$
β_{yx}	Least-squares regression slope of y on x
C	Cost of duplication event
C_{S}	Cost of host suppression
$\mathrm{Cov}(x,y)$	Covariance of x and y
$\mathrm{E}(x)$	Expectation of x
f_n or $f_{n,m}$	Frequency of hosts carrying n type-1 and m type-2 transposons at birth
F_n or $F_{n,m}$	Frequency of hosts carrying n type-1 and m type-2 transposons after proliferation
ϕ_n or $\phi_{n,m}$	Frequency of hosts carrying n type-1 and m type-2 transposons after selection
f'_n or $f'_{n,m}$	Frequency of hosts carrying n type-1 and m type-2 transposons at birth, in the next generation
g_k or $g_{k,\ell}$	Frequency of gametes carrying k type-1 and ℓ type-2 transposons
H	Haploid number of chromosomes in individual-based model
h_{S}	Half-saturation constant for suppression
h_{P}	Half-saturation constant for parasitism
i, j, k, ℓ	General purpose indices
K	Number of hosts in individual-based model
L	Number of occupable sites per chromosome in individual-based model
m	Number of mutant (type-2) transposons
μ_s	Mutation rate of host suppression in individual-based model
μ_u	Mutation rate of duplication rate in individual-based model
n	Number of wild-type (type-1) transposons
$n_{\max}$	Upper limit on transposon number in numerical simulations
$\bar{n}$	Population average number of wild-type transposons
N_0	Average experienced number of transposons prior to the duplication event
$N^{\mathrm{Off}}_{\mathrm{Pr},t}$	Average experienced number of transposons of an offspring transposon at generation t, private replication
$N^{\mathrm{Off}}_{\mathrm{Pu},t}$	Average experienced number of transposons of an offspring transposon at generation t, public replication
N^{Par}_t	Average experienced number of transposons of a parent transposon at generation t
p	Saturating probability of parasitism
$P(A')$	Probability of parasitism given total parasite activity A'
$\mathbb{P}(x\|\lambda)$	Poisson probability mass function
$\mathbb{Q}(x\|u)$	Quasibinomial probability mass function
r, R	Relatedness
s	Host suppression allelic value
S	Host suppression total value
σ_s, τ_s	Absolute and relative mutation step size, respectively, for host suppression in individual-based model
σ_u, τ_u	Absolute and relative mutation step size, respectively, for duplication rate in individual-based model
θ	Frequency of hosts carrying at least one mutant transposon
u	Duplication rate of transposons
U	"Effective duplication rate" of transposons
v	Excision (loss) rate of transposons
$\mathrm{Var}(x)$	Variance of x
$w(n)$	Host fitness function
$\bar{w}$	Population average fitness
W_0	Average experienced host relative fitness prior to the duplication event
$W^{\mathrm{Off}}_{\mathrm{Pr},t}$	Average experienced host relative fitness of an offspring transposon at generation t, private replication
$W^{\mathrm{Off}}_{\mathrm{Pu},t}$	Average experienced host relative fitness of an offspring transposon at generation t, public replication
W^{Par}_t	Average experienced host relative fitness of a parent transposon at generation t
χ	Mean number of chiasmata in individual-based model

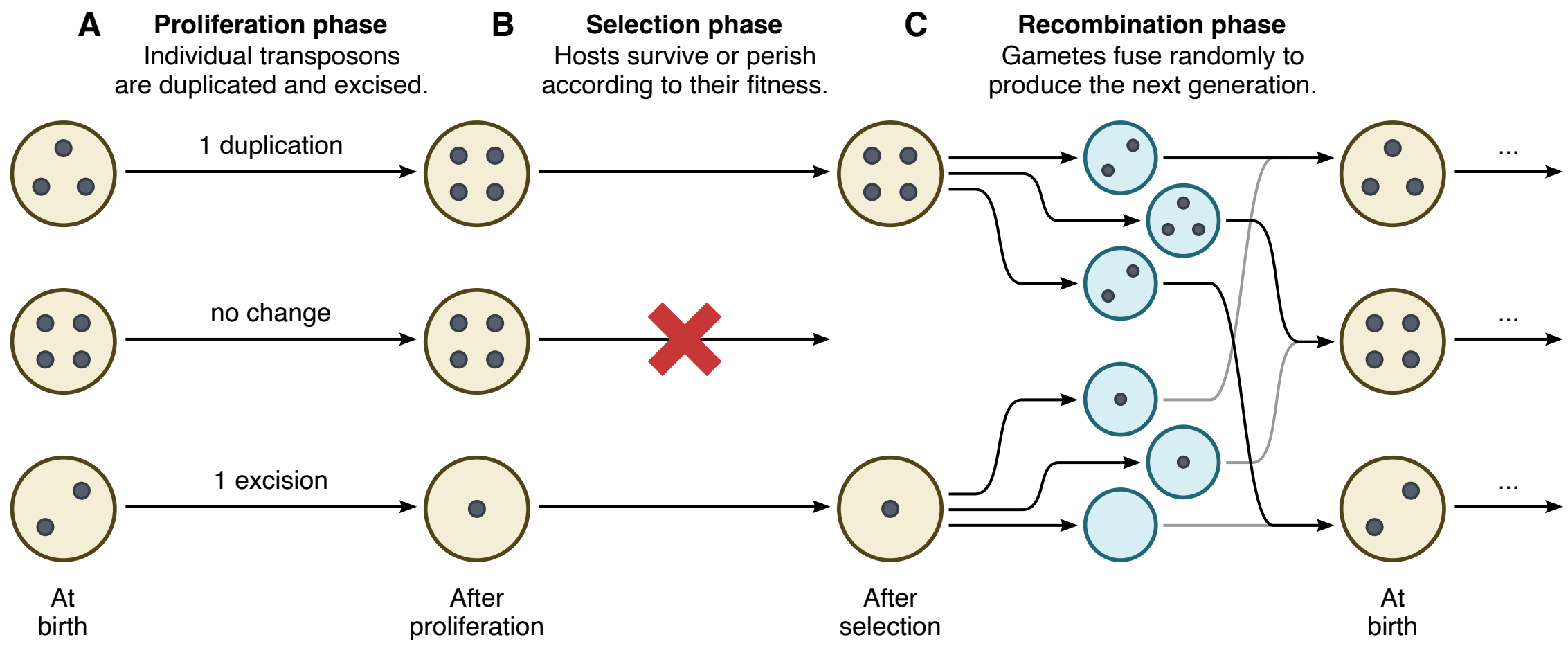

Figure S1: Diagram of the deterministic one-type model. (A) At birth, the host population exhibits a certain distribution of transposon copy numbers across each individual. In the proliferation phase, each transposon may duplicate zero or more times (depending upon the duplication rate, u) and/or may be excised (depending upon the excision probability v). (B) In the selection phase, hosts survive to reproductive age with probability proportional to their fitness $w(n)$, a decreasing function of the number of transposons carried by the host, and otherwise die. (C) In the recombination phase, each surviving host generates a large number of haploid gametes, which then fuse randomly with other gametes to produce the next generation of hosts.

Accordingly, after proliferation, the frequency F_n of hosts carrying n transposons is

$$F_n = \sum_i \sum_k f_i \mathbb{P}(k|iu)\mathbb{B}(i-n+k|i,v)\,, \tag{1}$$

where $\mathbb{P}(x|\lambda) = \lambda^x e^{-\lambda}/x!$ is the Poisson probability mass function and $\mathbb{B}(x|n,p) = \binom{n}{x}p^x(1-p)^{n-x}$ is the binomial probability mass function. The summand is the frequency f_i of hosts initially carrying i transposons, times the probability $\mathbb{P}(k|iu)$ that these hosts undergo k duplications, times the probability $\mathbb{B}(i{-}n{+}k|i,v)$ that in the same hosts, $i{-}n{+}k$ of the original i transposons are lost to excision, such that overall the summand $f_i\mathbb{P}(k|iu)\mathbb{B}(i-n+k|i,v)$ is equal to the frequency of hosts that have transitioned from carrying i to n transposons, having undergone k duplications and the required $i-n+k$ excisions.

Note that, in order for equation 1 to make biological sense, the number of excisions $i-n+k$ should be neither less than zero nor greater than the number of transposons at risk of being excised. Therefore, we rely on the convention that $\mathbb{B}(x|n,p) = 0$ for $x < 0$ or $x > n$, or equivalently, we restrict the innermost sum to positive integers k satisfying $n-i \leq k \leq n$.

Second, in the **selection** phase (Fig. S1B), hosts either survive to reproductive age, with probability proportional to their fitness $w = w(n)$, or they die. That is, after selection, the frequency ϕ_n of hosts with n transposons is

$$\phi_n = \frac{w(n)}{\bar{w}} F_n\,, \tag{2}$$

where

$$\bar{w} = \sum_{i=0}^{\infty} F_i w(i) \tag{3}$$

is mean host fitness after proliferation.

Third, in the **recombination** phase (Fig. S1C), all surviving hosts generate a large number of haploid gametes, which randomly fuse with other haploid gametes to produce the next diploid generation. Accordingly, the frequency of gametes carrying k transposons is

$$g_k = \sum_{n=k}^{\infty} \phi_n \mathbb{B}(k|n, \tfrac{1}{2}) \tag{4}$$

and the frequency of individuals carrying n transposons in the next generation is

$$f'_n = \sum_{k=0}^{n} g_k g_{n-k} \,. \tag{5}$$

The proliferation, selection, and recombination phases are repeatedly iterated numerically to produce each subsequent generation.

Note that while the model is phrased above in terms of tracking genotype frequencies for hosts carrying anywhere from zero to any arbitrarily large number of transposons, in practice we only track genotype frequencies f_n for values of n up to a number $n_{\max}$ that is sufficiently large (typically $n_{\max} \geq 100$) to accurately capture population dynamics.

The deterministic one-type model is akin to the 'Transposition and Selection' model developed by Charlesworth and Charlesworth [1, §4(i)], except that we assume that the number of duplications per transposon per generation is Poisson-distributed with rate u, not Bernoulli-distributed (i.e., either zero or one duplications per transposon) with probability u, and we assume that the number of occupable sites in the genome [T in ref. 1] is effectively infinite.

1.1.1 Equilibrium copy number

In their model, Charlesworth and Charlesworth [1, equations 20a–c] find that, at equilibrium, the mean copy number $\bar{n} = \sum_n f_n n$ satisfies the condition

$$-\frac{\partial \ln w(\bar{n})}{\partial \bar{n}} \approx u - v \,. \tag{6}$$

This condition, which can be used to estimate the mean transposon copy number at equilibrium $\bar{n}$, applies equally to our deterministic one-type model. Here, we show how to derive condition 6 using Price's [2, 3] equation

$$\Delta \bar{z} = \frac{1}{\bar{w}} \left(\mathrm{Cov}(w_i, z_i) + \mathrm{E}(w_i \, \Delta z_i) \right) \,. \tag{7}$$

The Price equation describes how the mean value of some trait z changes due to selection and transmission between an ancestral and a descendant population. Members of the ancestral population are grouped by the index i, which can represent any arbitrary grouping of the ancestral population. Here, we shall take as the trait of interest z the transposon copy number n, and let the index i refer to all individuals in the ancestral population carrying $n = i$ transposons. For the ancestral population we shall take a generation of hosts at birth,

and for the descendant population we shall take the same generation of hosts after selection (Fig. S1). The recombination phase does not affect the mean transposon copy number across a population, so we do not need to account for this phase here. This gives

$$\Delta\bar{n} = \frac{1}{\bar{w}}\left(\mathrm{Cov}(w_i, n_i) + \mathrm{E}(w_i\,\Delta n_i)\right)\,, \tag{8}$$

where $\Delta\bar{n}$ is the change in mean copy number over one generation, $\bar{w}$ is mean host fitness as given in equation 3 (i.e. it is the mean host fitness after proliferation), and Δn_i is the change in mean transposon number after selection among all hosts which initially carried i transposons at birth—in other words, it captures the mean change in transposon number in this group of hosts following both the proliferation phase and the selection phase.

The covariance of two variables $\mathrm{Cov}(x, y)$ is equal to $\beta_{yx}\,\mathrm{Var}(x)$, where β_{yx} is the least-squares linear regression slope of dependent variable y on independent variable x, and the expectation of the product of two variables $\mathrm{E}(xy)$ is equal to $\mathrm{E}(x)\,\mathrm{E}(y) + \mathrm{Cov}(x, y)$. Accordingly, we can rewrite equation 8 as

$$\Delta\bar{n} = \frac{1}{\bar{w}}\left(\beta_{w_i n_i}\,\mathrm{Var}(n_i) + \mathrm{E}(w_i)\,\mathrm{E}(\Delta n_i) + \mathrm{Cov}(w_i, \Delta n_i)\right)\,. \tag{9}$$

The $\beta_{w_i n_i}\,\mathrm{Var}(n_i)$ term in equation 9 captures the effect of selection *between* ancestral groups on copy number. Here, we can approximate $\beta_{w_i n_i}$, the least-squares linear regression slope of host fitness after proliferation on transposon number at birth, with the partial derivative $\partial w/\partial n$ evaluated at $n = \bar{n}(1 + u - v)$. This is only an approximation because of the curvature of the function $w(n)$. We can also approximate $\mathrm{Var}(n_i)$ with $\bar{n}$, since copy number is roughly Poisson-distributed at equilibrium [1, §5(ii)]. Overall, this gives $\beta_{w_i n_i}\,\mathrm{Var}(n_i) \approx \bar{n}\,\partial w/\partial n|_{n=\bar{n}(1+u-v)}$.

The $\mathrm{E}(w_i)\,\mathrm{E}(\Delta n_i) + \mathrm{Cov}(w_i, \Delta n_i)$ term in equation 9 captures two effects through Δn_i: proliferation of transposons, and selection *within* ancestral groups. Selection within ancestral groups arises because, after the proliferation stage, each group of hosts that was originally carrying n transposons at birth will now carry a distribution of different numbers of transposons, because of the varying number of duplication and excision events across individuals within the group. Within this term, we can rewrite $\mathrm{E}(w_i)$ as $\bar{w}$ and approximate $\mathrm{E}(\Delta n_i)$ using $\bar{n}(u - v)$, which captures the effect of proliferation but ignores the relatively insubstantial effect of within-group selection (the much more substantial effect of selection *between* ancestral groups for copy number is still accounted for in the $\beta_{w_i n_i}\,\mathrm{Var}(n_i)$ term of equation 9). Similarly, ignoring the within-group selection effect, we can approximate $\mathrm{Cov}(w_i, \Delta n_i)$ with $\mathrm{Cov}(w_i, (u - v)n_i) = (u - v)\beta_{w_i n_i}\,\mathrm{Var}(n_i)$, which in turn (as above) we can approximate with $(u - v)\bar{n}\,\partial w/\partial n|_{n=\bar{n}(1+u-v)}$.

Making all of these substitutions and setting $\Delta\bar{n} = 0$ in equation 9 allows us to estimate the mean copy number $\bar{n}$ at equilibrium by solving

$$0 \approx \frac{1}{\bar{w}}\left(\frac{\partial w}{\partial n}|_{n=\bar{n}(1+u-v)}\bar{n}(1 + u - v) + \bar{w}\bar{n}(u - v)\right)\,. \tag{10}$$

To obtain equation 6, we approximate $1+u-v$ with 1 (with the justification that $u, v \ll 1$) and approximate $(\partial w/\partial n|_{n=\bar{n}})/\bar{w}$ with $\partial \ln w/\partial n|_{n=\bar{n}}$, which is exact when $\bar{w} = w(\bar{n})$.

1.2 Deterministic two-type model

The deterministic two-type model builds upon the deterministic one-type model by adding a second transposon type. Again, there is an infinite population of diploid, sexually-reproducing hermaphroditic hosts with non-overlapping generations. We call the two types of transposon type 1 and type 2, and we notate the frequency of individuals carrying n type-1 transposons and m type-2 transposons as $f_{n,m}$. The two transposon types differ in duplication rate, u_1 and u_2.

Because this model introduces a second transposon type, the distinction between private versus public replication (see main text) changes the predictions of the model whenever $u_1 \neq u_2$. Here, a transposon's "effective duplication rate" depends upon the mode of replication. Under private replication, the effective duplication rate is equal to u_1 for type-1 transposons and u_2 for type-2 transposons; but under public replication, the effective duplication rate is equal to $(nu_1 + mu_2)/(n + m)$ in a genome with n type-1 transposons and m type-2 transposons, for transposons of either type. Additionally, the distinction between copy-number costs and activity costs (see main text) becomes meaningful in the two-type model, since under activity costs, the fitness of a host depends upon how many of its transposons are of type 1 versus type 2. Otherwise, this model is very similar to the deterministic one-type model, and we use similar terminology and notation to describe it here. As before, the population passes through proliferation, selection, and recombination phases each generation.

After **proliferation**, the frequency of hosts carrying n type-1 and m type-2 transposons is

$$F_{n,m} = \sum_i \sum_j \sum_k \sum_\ell f_{i,j} \mathbb{P}(k|iU_1) \mathbb{B}(i - n + k|i, v) \mathbb{P}(\ell|jU_2) \mathbb{B}(j - m + \ell|j, v)\,, \qquad (11)$$

where U_1 and U_2 are the "effective duplication rates" of transposon types 1 and 2, respectively; under private replication, $U_1 = u_1$ and $U_2 = u_2$, and under public replication, $U_1 = U_2 = (iu_1 + ju_2)/(i + j)$. As before, for equation 11 to make biological sense, we require either that $\mathbb{B}(x|n, p) = 0$ for $x < 0$ or $x > n$, or, equivalently, that the inner sums are restricted to positive integers k, ℓ satisfying $n - i \leq k \leq n$ and $m - j \leq \ell \leq m$.

In the **selection** phase, hosts survive with probability proportional to their fitness $w = w(n, m)$, such that, after selection, the frequency of hosts carrying n type-1 and m type-2 transposons is

$$\phi_{n,m} = \frac{w(n, m)}{\bar{w}} F_{n,m}\,, \qquad (12)$$

where

$$\bar{w} = \sum_i \sum_j F_{i,j} w(i, j)\,. \qquad (13)$$

Note that the formulation of the fitness function $w(n, m)$ allows fitness to depend upon the transposon number $n + m$, upon total transposon activity $A = nu_1 + mu_2$, or both.

Finally, in the **recombination** phase, the frequency of gametes carrying k type-1 and ℓ type-2 transposons is

$$g_{k,\ell} = \sum_{n=k}^{\infty} \sum_{m=\ell}^{\infty} \phi_{n,m} \mathbb{B}(k|n, \tfrac{1}{2}) \mathbb{B}(\ell|m, \tfrac{1}{2}) \qquad (14)$$

and the frequency of individuals carrying n type-1 transposons and m type-2 transposons in the next generation is

$$f'_{n,m} = \sum_{k=0}^{n} \sum_{\ell=0}^{m} g_{k,\ell} g_{n-k,m-\ell} \,. \tag{15}$$

The proliferation, selection, and recombination phases are repeatedly iterated to produce each subsequent generation.

1.3 Stochastic individual-based model

The stochastic individual-based model also analyses a population of diploid, sexually-reproducing hermaphroditic hosts with non-overlapping generations. However, there are several differences between this model and the deterministic models described above. First, the population size is finite. Second, and most importantly, each transposon has its own duplication rate u which can freely mutate over time. Third, we explicitly track the chromosomal position of each transposon in its genome, and allow for linkage disequilibrium between transposons. Finally, in the individual-based model we allow for the evolution of host suppression of transposon activity, and also consider the effect of "parasitic" transposon replication (in addition to public and private replication).

The model specification is as follows. There are K hosts, indexed by $i \in \{1, 2, \ldots, K\}$. Each diploid host has $2H$ linear chromosomes, where each chromosome comprises L occupable sites. Sites can be empty or occupied by a transposon. Individual i has n_i transposons, indexed by $j \in \{1, 2, \ldots, n_i\}$. We denote the duplication rate of the ith's individual's jth transposon by $u_{i,j}$, and its position as being at site $\ell_{i,j} \in \{1, 2, \ldots, L\}$ on chromosome $c_{i,j} \in \{1, 2, \ldots, 2H\}$. Sites cannot be occupied by more than one transposon. At simulation start, all transposons have duplication rate $u_{i,j} = u_0$ and are located at a random chromosome and site within their host.

A given generation begins with an **updating** stage, which corresponds to the proliferation phase of the deterministic models, and proceeds as follows. For each host i, we first generate three random integers: $k_i^{\mathrm{d}} \sim \mathrm{Poisson}(\sum_j u_{i,j})$, the number of duplications, $k_i^{\mathrm{e}} \sim \mathrm{Binomial}(n_i, v)$, the number of excisions, and $k_i^{\mathrm{m}} \sim \mathrm{Binomial}(n_i, \mu_u)$, the number of duplication-rate mutations, where μ_u is the per-generation, per-transposon mutation rate. Then each of these events takes place. First, k_i^{d} of the host's transposons are selected randomly with replacement, where the probability of transposon j being selected is $u_{i,j}/(\sum_j u_{i,j})$ under private replication and $1/n_i$ under public replication, and the duplication rates of these transposons are saved for later use. Then, k_i^{m} transposons are selected randomly with replacement, and for each selected transposon, the duplication rate $u_{i,j}$ is changed to $u'_{i,j} = \max(0, u_{i,j} + \delta u)$, where δu is drawn from a normal distribution with mean 0 and standard deviation $\sigma_u + \tau_u u_{i,j}$ (that is, there is both an absolute component σ_u and a relative component τ_u of transposon mutation). Then, k_i^{e} transposons are selected randomly without replacement and are deleted from the host genome. Finally, copies of the k_i^{d} transposons selected earlier are inserted into randomly-selected empty sites, making use of the previously-saved duplication rates.

After all hosts have been updated, the **reproduction** stage occurs, which corresponds to the selection and recombination phases of the deterministic models. K offspring are to be

generated to replace the current generation. For each offspring to be generated, we select two parents, with replacement, from the current generation, where each parent is selected with probability proportional to its fitness. We generate a gamete from each of the two parents via meiosis, including crossing-over between chromosome pairs at randomly-selected loci for each chromosome. The number of chiasmata (crossing-over sites) per chromosome pair is drawn from a Poisson distribution with mean χ, and then the position of each chiasma is picked randomly, with uniform probability across all chromosomal sites. Gametes fuse to form K new individuals, returning the population to the beginning of the life cycle.

We assume that the population is extinct when mean fitness drops below 1×10^{-6}.

1.3.1 Host suppression

For host suppression, we modify the above steps as follows. Each host i carries two suppressor alleles, with values $s_{i,1}$ and $s_{i,2}$. At simulation start, $s_{i,1} = s_{i,2} = 0$ for all i. The total 'amount' of suppression for a given host is $S_i = s_{i,1} + s_{i,2}$. We assume that host suppression follows Michaelis-Menten dynamics, such that, in a host with suppression S_i, transposon duplication rates are multiplied by a factor $\frac{h_S}{h_S + S_i}$, where h_S is a half-saturation constant. In other words, when $S_i > 0$, the duplication rate of transposons is reduced.

During the updating stage, each suppressor allele is mutated with probability μ_s to a new value $s'_{i,\cdot} = \max(0, s_{i,\cdot} + \delta s)$, where δs is drawn from a normal distribution with mean 0 and standard deviation $\sigma_s + \tau_s s_{i,\cdot}$. Suppressor loci are assumed to be unlinked to transposon loci, and during gametogenesis, one randomly-selected suppressor allele is passed on to each gamete.

We assume that there is a fitness cost c_S associated with host suppression, i.e. each host's fitness w is multiplied by a factor $1 - c_S S_i$.

1.3.2 Parasitic transposon replication

For parasitic transposons, we assume that the binding of parasites to ribosomes follows Michaelis-Menten dynamics. Specifically, in a host where the total activity of the parasitic transposons is A', the proportion of all duplication events that are parasitized is given by $P(A') = p\frac{A'}{h_P + A'}$, where h_P is a half-saturation constant, and p is the saturating probability of parasitism. Accordingly, for each duplication event in a given host, with probability $P(A')$, a randomly-selected parasitic transposon is duplicated, and with probability $1 - P(A')$, a randomly-selected non-parasitic transposon is duplicated. In each case, the random selection of the parasitic or non-parasitic transposon occurs with probability proportional to that parasitic or non-parasitic transposon's duplication rate u. Note that we only consider parasitic transposons in the context where non-parasitic transposons replicate privately.

In the main text, we show model runs with parasitic transposons for $p = 0.9$ and $p = 1.0$. Fig. S10 explores a wider range of values for p.

1.4 Parameter assumptions

We assume that $c_S = 1 \times 10^{-7}$. This is enough of a cost to select against needless suppression, without preventing hosts from adequately suppressing transposons. Sensitivity analysis of

this cost, which is shown in Fig. S11, demonstrates that our conclusions around how host suppression changes the fate of the host population is robust across a range of values for the cost of suppression.

Across all simulations, $u_0 = 0.005, v = 0.0005, H = 2, \chi = 0.9, \mu_u = 0.01, \sigma_u = 0.001, \tau_u = 0.05, \mu_s = 0.025, \sigma_s = 0.05, \tau_s = 0.25, h_{\mathrm{P}} = 100$, and $h_{\mathrm{S}} = 10$. For Figs. 1G and 1J of the main text, $K = 50000$; for Figs. 3C, 3D, 3E, 3G, 4C, and 4D, $K = 5000$; for Figs. 4E and 4F, $K = 10000$. For Fig. 1, we use the ectopic recombination fitness model (see section 3.1) with $\lambda\varepsilon\rho/100 = 0.00005$.

We also show in Fig. S12 model runs with host suppression of publicly replicating elements, as mentioned in the main text.

2 Invasion analysis

The models detailed above allow us to simulate the evolution of the transposon duplication rate under public versus private replication. However, they do not directly provide insight into why the outcome of transposon–host coevolution differs between these two scenarios. Therefore, in the following section, we will mathematically analyze the behaviour of the deterministic two-type model described above, in order to explain the difference in evolutionary outcomes under public versus private replication. We achieve this by performing an 'invasion analysis' that yields mathematical conditions for when natural selection favours an increase to the transposon duplication rate. We will use similar notation and terminology here as for the deterministic two-type model described above.

We approach this problem by focusing on a rare lineage of mutant transposons, interspersed among a much larger group of wild-type transposons, all circulating within a host population (Fig. S2A). To start with, we assume that the mutant lineage has the exact same duplication rate as the wild-type transposons do. We imagine that both the wild-type and mutant transposons have reached equilibrium in terms of how they are distributed across hosts.

Then, we assume that a "duplication event" occurs in which a single randomly-selected mutant transposon (the "parent") creates one extra copy (the "offspring") during the proliferation phase of the host life cycle (Fig. S2A). This extra copy—the offspring—is created over and above any other copies that may be made in the normal course of the proliferation phase. Under private replication, the offspring is necessarily a copy of the parent, but under public replication, the offspring is a copy of a randomly-selected transposon within the same host genome, including potentially the parent.

The duplication event realizes both a fitness benefit and a fitness cost for the parent. The benefit is that the offspring is, at least potentially, a copy of the parent transposon. The cost is that in creating the offspring, the parent "pollutes" its host's genome with an additional transposon, which reduces its own fitness. If the benefit exceeds the cost, this implies that natural selection would favour a small increase to the duplication rate. Accordingly, we can work out the conditions under which the duplication rate is selected to increase by mathematically analysing a duplication event as we have sketched it out above.

2.1 Details of the approach

It will be helpful to fill in a few details before proceeding. Costs and benefits are going to be measured in units of reproductive value [4], which in this case can be thought of as "transposon equivalents"—that is, a cost or benefit of 1 is equivalent to there being one fewer or one more mutant transposon present in the population, after enough generations have passed that the population is once again at equilibrium. (The 'at equilibrium' perspective is required because actually deleting or inserting a mutant transposon changes its host's fitness, which may have knock-on effects on other mutant transposons and, if applicable, on the inserted mutant transposon itself. We therefore need to wait a certain number of generations until the full effects of the insertion or deletion of a transposon have developed.)

This perspective makes the calculation of costs and benefits equivalent to the question: looking many generations into the future, are there more mutant transposons than there were immediately before the duplication event? The change in the number of mutant transposons

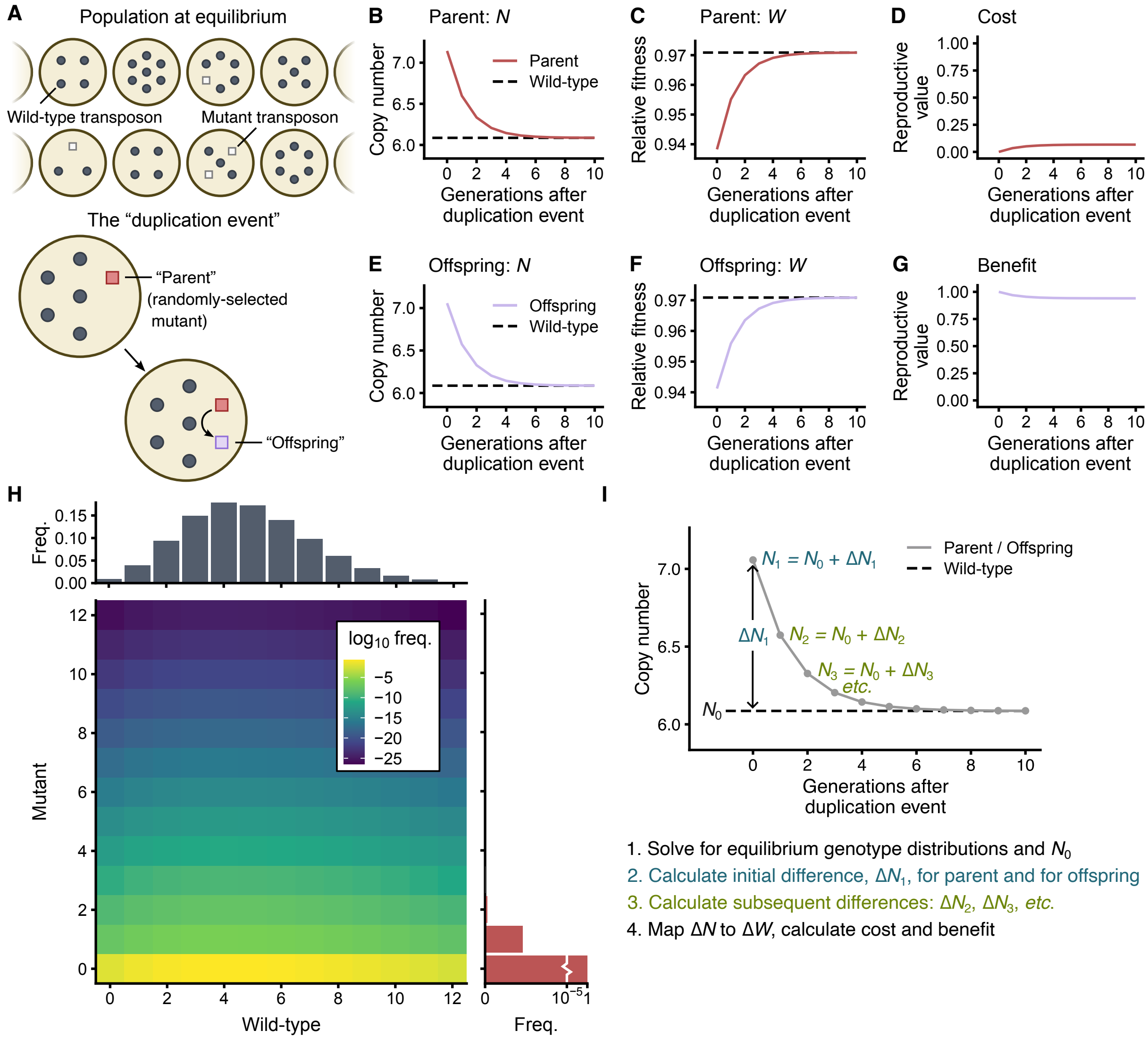

Figure S2: Schematic of the invasion analysis approach. (A) The invasion analysis takes as its starting point a population with a rare mutant transposon lineage co-circulating in a population at equilibrium with a much larger number of wild-type transposons. A “duplication event”, in which a randomly-selected mutant transposon (the “parent”) generates one additional transposon (the “offspring”) is analyzed to determine whether natural selection would favour a small increase to the duplication rate. (B) The duplication event increases the parent transposon’s experienced transposon number, and consequently (C) decreases its experienced host relative fitness; this incurs (D) a cost to the mutant lineage. (E) The duplication event creates the offspring transposon in a genome where the transposon number has just increased by one (E), and consequently (F) the offspring experiences a temporary reduction in experienced relative host fitness; this tempers (G) the benefit to the mutant lineage of the duplication event. (H) Illustrative frequency distribution of host genotypes when there is a rare mutant transposon lineage in an otherwise wild-type transposon-carrying population. The assumption that the host population in which the duplication event occurs contains only a vanishingly small proportion of hosts carrying mutant transposons simplifies the analysis. (I) The steps followed in the text to perform the invasion analysis.

is numerically equal to the benefit minus the cost of the duplication event, provided we measure costs and benefits in the correct units of reproductive value.

Focusing on the "parent" and "offspring" transposons involved in the duplication event, the cost of the duplication event is the expected loss in reproductive value caused by the offspring to the parent that generated it (and to any other mutants in the same genome, other than to the offspring itself), because of the reduction in host fitness associated with the burden of carrying one additional transposon. Equivalently, it is the expected number of mutant transposons that are removed from the population as a consequence of this reduction in host fitness. This cost is the same regardless of whether replication is public or private, since the appearance of one additional wild-type or mutant transposon has the same detrimental effect on host fitness for the parent.

Specifically, when the duplication event occurs, the number of transposons in the parent transposon's host increases by one. This means that the expected transposon number from the perspective of the parent (the parent's "experienced transposon number", N) increases relative to the expected transposon number from the perspective of a random wild-type transposon (Fig. S2B). This increase in experienced transposon number for the parent translates into an immediate reduction in the parent's experienced relative host fitness, W (Fig. S2C). Over the next several generations, repeated recombination events gradually dissociate the parent from its offspring, which means that the effect on the parent's experienced relative fitness dissipates over time (Fig. S2B–C). However, this temporary reduction in relative fitness results in an increased risk that the parent will be eliminated by selection between hosts, and by integrating this increased risk over generations we can calculate the overall cost of the duplication event in terms of lost reproductive value to the mutant lineage (Fig. S2D).

By contrast, the benefit of the duplication event is the value of the offspring from the perspective of the mutant lineage. If there were no 'knock-on effects' of the duplication event, the benefit would just be 1 if the offspring was a copy of the mutant lineage, and 0 if the offspring was a copy of the wild-type lineage. However, knock-on effects complicate this calculation.

Specifically, when the duplication event occurs, the offspring is created in a genome in which the transposon number has just increased by one. Therefore, the offspring experiences a (usually) higher-than-average transposon number (Fig. S2E), which results in a (usually) lower-than-average experienced relative fitness (Fig. S2F). These effects also dissipate over time, but they temper the expected value of the offspring such that the overall benefit of the duplication event, when it is a mutant transposon that is copied, is not generally equal to 1 (Fig. S2G).

To summarize, our overall strategy for calculating costs and benefits is as follows. First, we need to solve for the joint distribution of mutant and wild-type transposons in a population at equilibrium. We can picture this joint distribution as a matrix where the marginal distributions (i.e. of wild-type and of mutant transposons) are also of interest (Fig. S2H). Then, we need to calculate the initial change in experienced transposon number for the parent and offspring transposons resulting from the duplication event; calculate how this initial change dissipates over multiple generations; and convert changes in experienced transposon number ΔN to changes in experienced relative fitness ΔW to calculate the overall cost and benefit of the duplication event. These steps are illustrated in Fig. S2I.

We will now work through each of these steps in turn. Readers who wish to skip

mathematical detail can proceed to section 2.8. For simplicity in this derivation, we shall ignore transposon excision by setting $v = 0$. A nonzero excision rate can be approximated by adopting a duplication rate $u' = u - v$.

2.2 Distribution of wild-type and mutant transposons at equilibrium

Recall that we are considering a rare lineage of mutant transposons circulating in a host population, interspersed among a much larger number of wild-type transposons. We assume that both transposon types have the same duplication rate, and their distribution among the host population has reached equilibrium. The first step of the analysis is to solve for this equilibrium joint distribution across the number of wild-type and mutant transposons carried by hosts in the population (Fig. S2H).

Suppose that, at equilibrium and at birth, the frequency of hosts carrying n wild-type transposons and m mutant transposons is $f_{n,m}$, where frequencies are normalized such that $\sum_{n,m} f_{n,m} = 1$. If the equilibrium distributions of wild-type and mutant transposons are approximately independent, $f_{n,m}$ can be decomposed into the product of the marginal distributions, which we shall write as $f_{n,m} \approx a_n b_m$. The equilibrium distributions of wild-type and mutant transposons are not independent, but as we demonstrate below, they are close enough to independent that this approximation is workable.

Analysing a model with a single transposon type, Charlesworth and Charlesworth [1] show that transposon copy number is approximately Poisson-distributed, because in a large population, there is negligible variation in transposon frequencies between different occupable sites—which makes transposon number follow a binomial distribution—and the number of occupable sites is large while the probability of any given site being occupied is small—which makes a Poisson approximation of the binomially-distributed transposon number valid under the Poisson limit theorem.

A further requirement for the Poisson limit theorem to hold is that events are independent, which in this context means that the presence or absence of a transposon at any given site within a host does not affect the probability of finding a transposon at any other site in the same host. In our model, broadly speaking, the processes of duplication and selection introduce dependence between transposons within hosts, while recombination strongly reduces such dependence by continually crossing in transposons from the broader population. Provided that duplication and selection are weak relative to recombination, the independence assumption approximately holds, and we will use the Poisson distribution $\mathbb{P}(n|\lambda)$, where $\lambda = \bar{n}$, for the marginal distribution of wild-type transposons a_n. To specify $\lambda = \bar{n}$, we can either estimate the equilibrium copy number using equation 6, or measure it from a given simulation of the one-type or two-type deterministic model.

For the marginal distribution of mutant transposons at equilibrium, b_m, the situation is entirely different because, when mutants are rare, recombination only separates mutant transposons from one another and does not also bring them together by crossing in. We therefore obtain b_m as follows. We assume that, in hosts carrying m mutant transposons, each transposon generates a Poisson-distributed number of copies at rate u. We assume selection is weak and ignore it. Then, each host generates a large number of gametes, where each

transposon carried by the host has a 50% chance of being represented in a given gamete, and because hosts carrying at least one mutant transposon are vanishingly rare by construction, the probability that a mutant-carrying host mates with another mutant-carrying host can be neglected. This yields a recurrence relation for the proportion of mutant-carrying hosts carrying m mutant transposons in the next generation of

$$b'_m = \eta \sum_{i=m}^{\infty} \sum_{j=1}^{i} b_j \mathbb{P}(i-j|ju)\mathbb{B}(m|i, \tfrac{1}{2}), \tag{16}$$

where η is a normalising constant. A numerical solution for the stationary distribution $b'_m = b_m$ can be found by iterating equation 16. We have not found an analytical solution for the stationary distribution b_m, but have found through numerical experiment that a suitable approximation for b_m is a "quasi-geometric" distribution

$$\mathbb{Q}(m|u) = \begin{cases} 1-\theta & \text{if } m = 0 \\ \theta(1-u)u^{m-1} & \text{if } m > 0 \end{cases}, \tag{17}$$

where $\theta \ll 1$ is the frequency of hosts carrying at least one mutant transposon. This approximation serves well particularly when u is small and selection is weak. The mean number of mutant transposons across the whole population is thus $\theta/(1-u)$, and the mean number of mutant transposons among those hosts carrying at least one transposon is $1/(1-u)$; these follow from the fact that the mean of a geometric distribution with probability mass function $\mathbb{G}(x|p) = p(1-p)^{x-1}$ is $1/p$.

To verify how well these approximations hold, we conducted a series of numerical simulations of the two-type deterministic model with a wild-type strain and a rare mutant strain, each with the same duplication rate, and the host fitness function $w(n) = (1-c)^{n^2/2}$, where c is referred to as the "unit cost". We tested a range of unit costs ($c \in \{0.0005, 0.005, 0.05\}$) and a suitable range of values for the duplication rate u under each cost. We find that the use of the Poisson distribution for a_n and the quasi-geometric distribution for b_m is generally accurate, except for atypically high values of the duplication rate u (Fig. S3 A–C) and that modelling the joint distribution over host genotypes $f_{n,m}$ as the product of the marginal distributions a_n and b_m is also generally accurate, except again for atypically high values of u (Fig. S3 D–E).

2.3 Experienced relative fitness and transposon number at equilibrium

We have now derived approximations for the equilibrium distribution at birth of wild-type and mutant transposons across a host population. Using this, we will now derive some "baseline" measurements of host relative fitness and transposon number from the perspective of a randomly-selected mutant transposon, in order to later consider how the duplication event incurs costs and benefits to the mutant lineage by changing these quantities.

After proliferation, the expected relative host fitness of a randomly-selected mutant transposon is

$$W_0 = \frac{1}{1+u}. \tag{18}$$

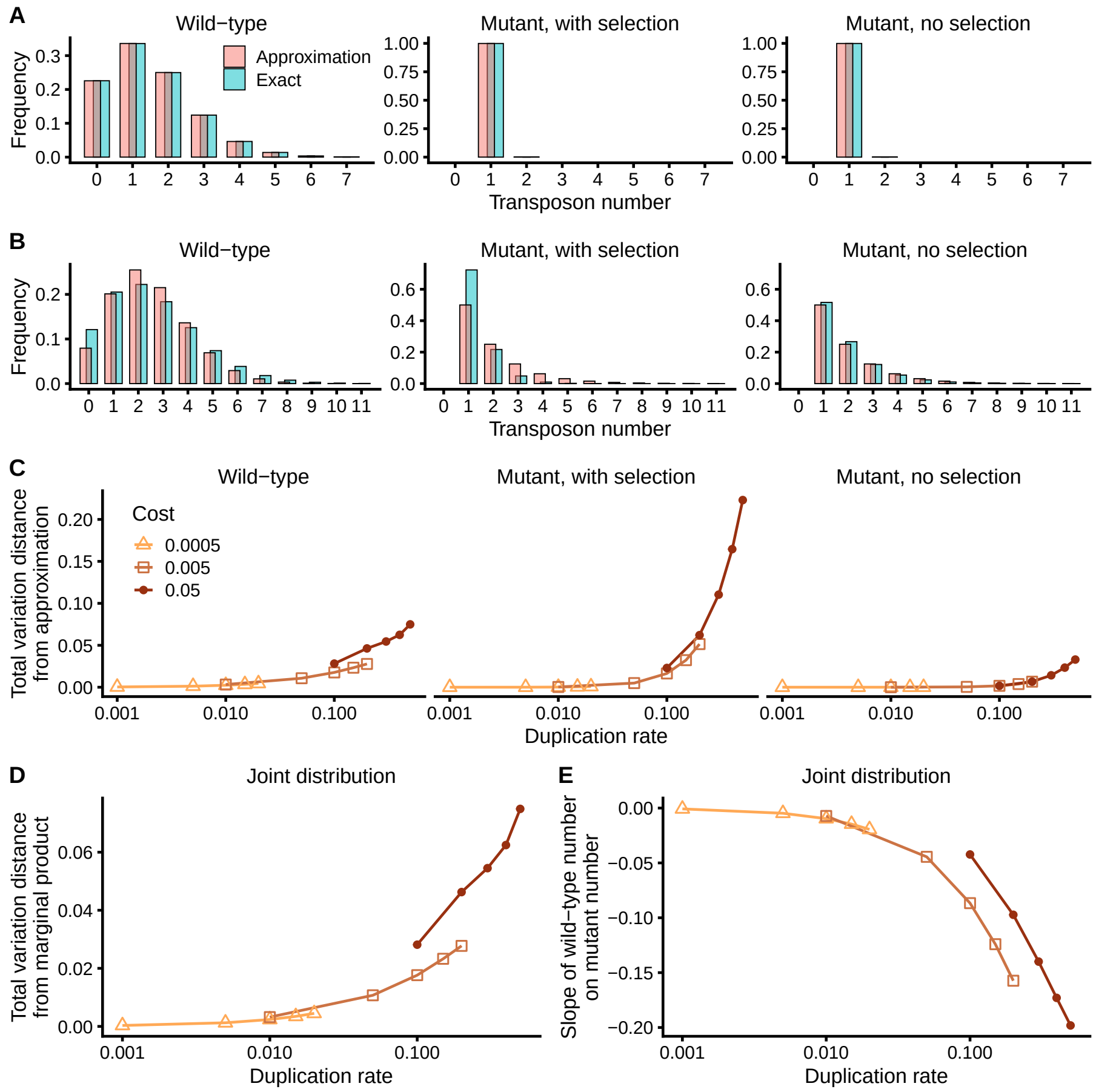

Figure S3: Assessment of approximations for the distribution of wild-type and mutant transposons in the invasion analysis. (A, B) Example comparisons of approximated and exact distributions for wild-type transposons (top row) and mutant transposons, excluding those hosts who do not carry any mutants (bottom row). For wild-type transposons, the approximation is a Poisson distribution, while for mutant transposons, the approximation is a "quasi-geometric" distribution (see section 2.2). Panel A shows a low-u scenario with weak selection ($u = 0.001$, $c = 0.0005$), while panel B shows a high-u scenario with strong selection ($u = 0.5$, $c = 0.05$); here c is the "unit cost" in the host fitness function $w(n) = (1-c)^{n^2/2}$. (C) Summary of assessments of approximations across all scenarios tested, for wild-type and mutant transposons with selection (from the two-type deterministic simulation) and for mutant transposons without selection (by iterating equation 16). The total variation distance, a measure of similarity between two distributions, is $\frac{1}{2}\sum_n |S(n) - A(n)|$, where $S(n)$ is the 'exact' distribution over n from simulations and $A(n)$ is the approximating distribution over n. Only scenarios where u is very high show substantial differences between the approximating and the true distributions. The reason for comparing the approximating quasi-geometric distribution with the mutant distribution in the absence of selection is to show that the reason why there is a discrepancy between the approximating and the exact distributions for mutant transposons, when the duplication rate is high, is the distorting effect of strong selection. (D, E) Assessment of independence of wild-type and mutant distributions. Panel D shows the total variation distance between the true joint distribution of wild-type and mutant transposons, $f_{n,m}$, and the product, $a_n b_m$, of their marginal distributions, $a_n = \sum_m f_{n,m}$ and $b_m = \sum_n f_{n,m}$. Panel E shows the slope of wild-type copy number on mutant copy number across all hosts, i.e. how much the number of wild-type transposons decreases, on average, for each mutant transposon present. By both metrics, the assumption of independence is justified except in scenarios where u is abnormally high.

To see why this has to be the case, note that at equilibrium, transposon number is neither increasing nor decreasing. Therefore, the growth in transposon number during the proliferation phase by a relative factor $1 + u$ has to be exactly cancelled out during the selection phase by the inverse of the same factor (given that the recombination phase does not affect transposon number).

Note that this same quantity can be calculated by explicitly averaging over the relative host fitness across all mutant transposons in the population:

$$W_0 = \frac{\sum_{n=0}^{\infty} \sum_{m=1}^{\infty} f_{n,m} \sum_{i=0}^{\infty} \mathbb{P}(i|nu) \sum_{j=0}^{\infty} \mathbb{P}(j|mu)(m+j)\ w(n+m+i+j)/\bar{w}}{\sum_{n=0}^{\infty} \sum_{m=1}^{\infty} f_{n,m} \sum_{i=0}^{\infty} \mathbb{P}(i|nu) \sum_{j=0}^{\infty} \mathbb{P}(j|mu)(m+j)}\,. \tag{19}$$

Above, $f_{n,m}\,\mathbb{P}(i|nu)\,\mathbb{P}(j|mu)(m+j)$ is a quantity proportional to the probability that, after proliferation, a randomly-selected mutant transposon resides in a host with n wild-type transposons that have just duplicated i times and m mutant transposons that have just duplicated j times, and $w(n+m+i+j)/\bar{w}$ is the relative fitness of that transposon's host, such that overall, the expression above calculates the expectation of the host's relative fitness across all mutant transposons in the population.

Similarly, after proliferation, the expected number of transposons carried in the host genome of a randomly-selected mutant transposon is

$$N_0 = \frac{\sum_{n=0}^{\infty} \sum_{m=1}^{\infty} f_{n,m} \sum_{i=0}^{\infty} \mathbb{P}(i|nu) \sum_{j=0}^{\infty} \mathbb{P}(j|mu)(m+j)\ (n+m+i+j)}{\sum_{n=0}^{\infty} \sum_{m=1}^{\infty} f_{n,m} \sum_{i=0}^{\infty} \mathbb{P}(i|nu) \sum_{j=0}^{\infty} \mathbb{P}(j|mu)(m+j)}\,. \tag{20}$$

Above, $f_{n,m}\,\mathbb{P}(i|nu)\,\mathbb{P}(j|mu)(m+j)$ is a quantity proportional to the probability that, after proliferation, a randomly-selected mutant transposon resides in a host with n wild-type transposons that have just duplicated i times and m mutant transposons that have just duplicated j times, and $n+m+i+j$ is the total number of transposons carried by that host, such that overall, the expression above calculates the expectation of the host's transposon number across all mutant transposons in the population.

In general, we will refer to the expectation of a quantity over a randomly-selected transposon as the "experienced" version of that quantity, i.e. N is referred to as the experienced transposon number and W is referred to as the experienced relative fitness. N differs from $\bar{n}$ in that N is the average transposon number over transposons, while $\bar{n}$ is the average transposon number over hosts, and W differs from $\bar{w}$ in that W is the average *relative* fitness over transposons, while $\bar{w}$ is the average *absolute* fitness over hosts.

Returning to equation 20, we can eliminate the inner two infinite sums by taking advantage of the fact that $\sum_x f(x)x^n$ for a discrete probability distribution with PMF $f(x)$ is just the nth (raw) moment of that distribution; for the Poisson distribution $\mathbb{P}(x|\lambda)$, the first moment

is λ and the second moment is $\lambda(\lambda+1)$, so we have

$$\begin{aligned} N_0 &= \frac{\sum_{n=0}^{\infty}\sum_{m=1}^{\infty} f_{n,m} \sum_{i=0}^{\infty} \mathbb{P}(i|nu) \sum_{j=0}^{\infty} \mathbb{P}(j|mu)(m(n+m+i)+j(n+2m+i)+j^2)}{\sum_{n=0}^{\infty}\sum_{m=1}^{\infty} f_{n,m} \sum_{i=0}^{\infty} \mathbb{P}(i|nu) \sum_{j=0}^{\infty} \mathbb{P}(j|mu)(m+j)} \\ &= \frac{\sum_{n=0}^{\infty}\sum_{m=1}^{\infty} f_{n,m} \sum_{i=0}^{\infty} \mathbb{P}(i|nu)\,(m(n+m+i)+mu(n+2m+i)+mu(mu+1))}{\sum_{n=0}^{\infty}\sum_{m=1}^{\infty} f_{n,m} \sum_{i=0}^{\infty} \mathbb{P}(i|nu)\,(m+mu)} \\ &= \frac{\sum_{n=0}^{\infty}\sum_{m=1}^{\infty} f_{n,m}\,(m(n+m+nu)+mu(n+2m+nu)+mu(mu+1))}{\sum_{n=0}^{\infty}\sum_{m=1}^{\infty} f_{n,m}\,(m+mu)} \\ &= \frac{\sum_{n=0}^{\infty}\sum_{m=1}^{\infty} f_{n,m}\,(m(n(1+u)^2+u)+m^2(1+u)^2)}{\sum_{n=0}^{\infty}\sum_{m=1}^{\infty} f_{n,m}\,(m(1+u))}\,. \end{aligned}$$

Now we substitute our approximate, marginal-product distribution $a_n\mathbb{Q}(m|u)$ for $f_{n,m}$:

$$\begin{aligned} N_0 &\approx \frac{\sum_{n=0}^{\infty} a_n \sum_{m=1}^{\infty} \mathbb{Q}(m|u)\,(m(n(1+u)^2+u)+m^2(1+u)^2)}{\sum_{n=0}^{\infty} a_n \sum_{m=1}^{\infty} \mathbb{Q}(m|u)\,(m(1+u))} \qquad (21) \\ &= \frac{\sum_{n=0}^{\infty} a_n \left(\frac{1}{1-u}(n(1+u)^2+u)+\frac{1+u}{(1-u)^2}(1+u)^2\right)}{\sum_{n=0}^{\infty} a_n \left(\frac{1}{1-u}(1+u)\right)} \\ &= \frac{\frac{1}{1-u}(\bar{n}(1+u)^2+u)+\frac{1+u}{(1-u)^2}(1+u)^2}{\frac{1}{1-u}(1+u)}\,, \end{aligned}$$

where we have again used definitions of moments of probability distributions to eliminate the infinite sums, specifically $\sum_m \mathbb{Q}(m|u)m = \theta/(1-u)$, $\sum_m \mathbb{Q}(m|u)m^2 = \theta(1+u)/(1-u)^2$, and $\sum_n a_n n = \bar{n}$.

Finally, this yields

$$N_0 = \frac{\bar{n}(1-u^2)+(1+u)^2+u\frac{1-u}{1+u}}{1-u} \qquad (22)$$

as the "baseline" experienced transposon number for a randomly-selected mutant transposon after proliferation. This completes step 1 of the invasion analysis as shown in Fig. S2I.

2.4 Benefit: Initial difference ΔN_1 for the offspring transposon

We now focus on what happens during the duplication event, in which the randomly-selected mutant transposon (the parent) creates one extra copy (the offspring) during the proliferation phase (Fig. S2A).

The benefit of the duplication event is the reproductive value of the offspring from the perspective of the mutant lineage. Calculating this value requires knowing the experienced relative fitness of the offspring immediately after the duplication event, which we write as W_1^{Off}, as well as its experienced relative fitness in later generations, W_2^{Off}, W_3^{Off}, *etc.* Under private replication, we shall write these quantities as $W_{\mathrm{Pr},1}^{\mathrm{Off}}$, *etc.*, and under public replication, we shall write these quantities as $W_{\mathrm{Pu},1}^{\mathrm{Off}}$, *etc.*

Under private replication, the experienced relative fitness of the offspring immediately after the duplication event is

$$W_{\mathrm{Pr},1}^{\mathrm{Off}} = \frac{\sum_{n=0}^{\infty}\sum_{m=1}^{\infty} f_{n,m} \sum_{i=0}^{\infty} \mathbb{P}(i|nu) \sum_{j=0}^{\infty} \mathbb{P}(j|mu)m\; w(n+m+i+j+1)/\bar{w}}{\sum_{n=0}^{\infty}\sum_{m=1}^{\infty} f_{n,m} \sum_{i=0}^{\infty} \mathbb{P}(i|nu) \sum_{j=0}^{\infty} \mathbb{P}(j|mu)m}\,, \qquad (23)$$

where $f_{n,m}\,\mathbb{P}(i|nu)\,\mathbb{P}(j|mu)m$ is proportional to the probability that the offspring resides in a host with n wild-type transposons that have just duplicated i times and m mutant transposons that have just duplicated j times, and $w(n+m+i+j+1)/\bar{w}$ is the relative fitness of the offspring transposon's host. We can use this expression to calculate the difference in expected relative host fitness between the offspring transposon and a randomly-selected mutant as $\Delta W^{\text{Off}}_{\text{Pr},1} = W^{\text{Off}}_{\text{Pr},1} - W_0$.

Similarly, the expected number of transposons carried in the host genome of the extra copy, at the moment after the proliferation stage when the extra copy is created, is

$$N^{\text{Off}}_{\text{Pr},1} = \frac{\sum_{n=0}^{\infty}\sum_{m=1}^{\infty} f_{n,m} \sum_{i=0}^{\infty}\mathbb{P}(i|nu)\sum_{j=0}^{\infty}\mathbb{P}(j|mu)m\,(n+m+i+j+1)}{\sum_{n=0}^{\infty}\sum_{m=1}^{\infty} f_{n,m} \sum_{i=0}^{\infty}\mathbb{P}(i|nu)\sum_{j=0}^{\infty}\mathbb{P}(j|mu)m}\,. \tag{24}$$

Equation 23 for $W^{\text{Off}}_{\text{Pr},1}$ is difficult to calculate for all but the simplest forms of the fitness function $w(n)$, but equation 24 for $N^{\text{Off}}_{\text{Pr},1}$ is more tractable. If we calculate $\Delta N^{\text{Off}}_{\text{Pr},1} = N^{\text{Off}}_{\text{Pr},1} - N_0$, we can approximate $\Delta W^{\text{Off}}_{\text{Pr},1}$ by

$$\Delta W^{\text{Off}}_{\text{Pr},1} \approx \frac{\beta_{wn}}{\bar{w}} \Delta N^{\text{Off}}_{\text{Pr},1}\,. \tag{25}$$

We proceed with this as follows:

$$\begin{aligned}
N^{\text{Off}}_{\text{Pr},1} &= \frac{\sum_{n=0}^{\infty}\sum_{m=1}^{\infty} f_{n,m} \sum_{i=0}^{\infty}\mathbb{P}(i|nu)\sum_{j=0}^{\infty}\mathbb{P}(j|mu)m(n+m+i+j+1)}{\sum_{n=0}^{\infty}\sum_{m=1}^{\infty} f_{n,m} \sum_{i=0}^{\infty}\mathbb{P}(i|nu)\sum_{j=0}^{\infty}\mathbb{P}(j|mu)m} \\
&= \frac{\sum_{n=0}^{\infty}\sum_{m=1}^{\infty} f_{n,m} \sum_{i=0}^{\infty}\mathbb{P}(i|nu)m(n+m+i+mu+1)}{\sum_{n=0}^{\infty}\sum_{m=1}^{\infty} f_{n,m} \sum_{i=0}^{\infty}\mathbb{P}(i|nu)m} \\
&= \frac{\sum_{n=0}^{\infty}\sum_{m=1}^{\infty} f_{n,m}m(n+m+nu+mu+1)}{\sum_{n=0}^{\infty}\sum_{m=1}^{\infty} f_{n,m}m} \\
&\approx \frac{\sum_{n=0}^{\infty} a_n \sum_{m=1}^{\infty}\mathbb{Q}(m|u)m(n+m+nu+mu+1)}{\sum_{n=0}^{\infty} a_n \sum_{m=1}^{\infty}\mathbb{Q}(m|u)m} \\
&= \frac{\sum_{n=0}^{\infty} a_n \sum_{m=1}^{\infty}\mathbb{Q}(m|u)(m(n(1+u)+1)+m^2(1+u))}{\sum_{n=0}^{\infty} a_n \sum_{m=1}^{\infty}\mathbb{Q}(m|u)m} \\
&= \frac{\sum_{n=0}^{\infty} a_n(\frac{1}{1-u}(n(1+u)+1)+\frac{1+u}{(1-u)^2}(1+u))}{\sum_{n=0}^{\infty} a_n\frac{1}{1-u}} \\
&= \frac{\frac{1}{1-u}(\bar{n}(1+u)+1)+\frac{1+u}{(1-u)^2}(1+u)}{\frac{1}{1-u}} \\
&= \frac{\bar{n}(1-u^2)+2+u(1+u)}{1-u}\,,
\end{aligned} \tag{26}$$

using similar techniques as in the derivation of equation 22.

This allows us to calculate $\Delta N^{\text{Off}}_{\text{Pr},1}$ as

$$\begin{aligned}
\Delta N^{\text{Off}}_{\text{Pr},1} &= N^{\text{Off}}_{\text{Pr},1} - N_0 \\
&= \frac{\bar{n}(1-u^2)+2+u(1+u)}{1-u} - \frac{\bar{n}(1-u^2)+(1+u)^2+u\frac{1-u}{1+u}}{1-u} \\
&= \frac{1}{1+u}\,.
\end{aligned} \tag{27}$$

Under public replication, we will take a similar approach to calculate

$$N^{\mathrm{Off}}_{\mathrm{Pu},1} = \frac{\sum_{n=0}^{\infty}\sum_{m=1}^{\infty} f_{n,m}\sum_{i=0}^{\infty}\mathbb{P}(i|nu)\sum_{j=0}^{\infty}\mathbb{P}(j|mu)m\frac{m}{n+m}\ (n+m+i+j+1)}{\sum_{n=0}^{\infty}\sum_{m=1}^{\infty} f_{n,m}\sum_{i=0}^{\infty}\mathbb{P}(i|nu)\sum_{j=0}^{\infty}\mathbb{P}(j|mu)m\frac{m}{n+m}}\,. \tag{28}$$

Above, $f_{n,m}\,\mathbb{P}(i|nu)\,\mathbb{P}(j|mu)m\frac{m}{n+m}$ is proportional to the probability that the offspring both (i) resides in a host with n wild-type transposons that have just duplicated i times and m mutant transposons that have just duplicated j times and (ii) is a mutant; and $n+m+i+j+1$ is the number of transposons carried by the offspring's host.

We proceed as follows:

$$\begin{aligned}
N^{\mathrm{Off}}_{\mathrm{Pu},1} &= \frac{\sum_{n=0}^{\infty}\sum_{m=1}^{\infty} f_{n,m}\sum_{i=0}^{\infty}\mathbb{P}(i|nu)m\frac{m}{n+m}(n+m+i+mu+1)}{\sum_{n=0}^{\infty}\sum_{m=1}^{\infty} f_{n,m}\sum_{i=0}^{\infty}\mathbb{P}(i|nu)m\frac{m}{n+m}}\\
&= \frac{\sum_{n=0}^{\infty}\sum_{m=1}^{\infty} f_{n,m}m\frac{m}{n+m}(n+m+nu+mu+1)}{\sum_{n=0}^{\infty}\sum_{m=1}^{\infty} f_{n,m}m\frac{m}{n+m}}\\
&\approx \frac{\sum_{n=0}^{\infty} a_n\sum_{m=1}^{\infty}\mathbb{Q}(m|u)m\frac{m}{n+m}(n+m+nu+mu+1)}{\sum_{n=0}^{\infty} a_n\sum_{m=1}^{\infty}\mathbb{Q}(m|u)m\frac{m}{n+m}}\,.
\end{aligned}$$

The innermost infinite sum cannot be reduced down to a closed-form expression, so we take only the first two terms of the sum, with the remainder being of order u^2, and approximate the wild-type distribution a_n with a Poisson distribution $\mathbb{P}(n|\bar{n})$:

$$N^{\mathrm{Off}}_{\mathrm{Pu},1} \approx \frac{\sum_{n=0}^{\infty}\mathbb{P}(n|\bar{n})\left(\frac{1-u}{n+1}(n(1+u)+2+u)+\frac{4(1-u)u}{n+2}(n(1+u)+3+2u)+\mathcal{O}(u^2)\right)}{\sum_{n=0}^{\infty}\mathbb{P}(n|\bar{n})\left(\frac{1-u}{n+1}+\frac{4(1-u)u}{n+2}+\mathcal{O}(u^2)\right)}$$

We ignore the terms in u^2 and proceed:

$$\begin{aligned}
N^{\mathrm{Off}}_{\mathrm{Pu},1} &\approx \frac{\sum_{n=0}^{\infty}\frac{\bar{n}^n\exp(-\bar{n})}{n!}\left(\frac{4+10u-6u^2-8u^3+n(4+15u-7u^2-12u^3)+n^2(1+4u-u^2-4u^3)}{(n+1)(n+2)}\right)}{\sum_{n=0}^{\infty}\frac{\bar{n}^n\exp(-\bar{n})}{n!}\left(\frac{(1-u)(2+4u)+n(1-u)(1+4u)}{(n+1)(n+2)}\right)}\\
&= \frac{\sum_{n=0}^{\infty}\frac{\bar{n}^n\exp(-\bar{n})}{(n+2)!}\left(4+10u-6u^2-8u^3+n(4+15u-7u^2-12u^3)+n^2(1+4u-u^2-4u^3)\right)}{\sum_{n=0}^{\infty}\frac{\bar{n}^n\exp(-\bar{n})}{(n+2)!}\left((1-u)(2+4u)+n(1-u)(1+4u)\right)}\\
&= \frac{\bar{n}^{-2}\sum_{n=0}^{\infty}\frac{\bar{n}^{n+2}\exp(-\bar{n})}{(n+2)!}\left(4+10u-6u^2-8u^3+n(4+15u-7u^2-12u^3)+n^2(1+4u-u^2-4u^3)\right)}{\bar{n}^{-2}\sum_{n=0}^{\infty}\frac{\bar{n}^{n+2}\exp(-\bar{n})}{(n+2)!}\left((1-u)(2+4u)+n(1-u)(1+4u)\right)}\\
&= \frac{\sum_{n=2}^{\infty}\mathbb{P}(n|\bar{n})\left(4+10u-6u^2-8u^3+(n-2)(4+15u-7u^2-12u^3)+(n-2)^2(1+4u-u^2-4u^3)\right)}{\sum_{n=2}^{\infty}\mathbb{P}(n|\bar{n})\left((1-u)(2+4u)+(n-2)(1-u)(1+4u)\right)}\\
&= \frac{\exp(\bar{n})(\bar{n}(1+4u)(1+\bar{n}(1+u))-4u)-\bar{n}+4u}{\exp(\bar{n})(\bar{n}+4u(\bar{n}-1))-\bar{n}+4u}\,.
\end{aligned} \tag{29}$$

Now that we have an expression for $N^{\mathrm{Off}}_{\mathrm{Pu},1}$, we can calculate $\Delta N^{\mathrm{Off}}_{\mathrm{Pu},1} = N^{\mathrm{Off}}_{\mathrm{Pu},1} - N_0$. But because we ignored terms in u^2 in estimating $N^{\mathrm{Off}}_{\mathrm{Pu},1}$ above, we should repeat the calculation

for N_0, making the same approximation, for better accuracy. We take up the calculation of N_0 from equation 21:

$$
\begin{aligned}
N_{\text{Pu},0}^{\text{Off}} &\approx \frac{\sum_{n=0}^{\infty}\mathbb{P}(n|\bar{n})\sum_{m=1}^{2}\mathbb{Q}(m|u)(m^2(1+u)^2+m(n(1+u)^2+u))}{\sum_{n=0}^{\infty}\mathbb{P}(n|\bar{n})\sum_{m=1}^{2}\mathbb{Q}(m|u)(m(1+u))} \\
&= \frac{\sum_{n=0}^{\infty}\mathbb{P}(n|\bar{n})\left((1-u)((1+u)^2+n(1+u)^2+u)+(1-u)u(4(1+u)^2+2n(1+u)^2+2u)+\mathcal{O}(u^2)\right)}{\sum_{n=0}^{\infty}\mathbb{P}(n|\bar{n})\left((1-u)(1+u)+(1-u)u(2(1+u))+\mathcal{O}(u^2)\right)} \\
&\approx \frac{\sum_{n=0}^{\infty}\mathbb{P}(n|\bar{n})(1+n(1+u)^2(1+2u)+u(1+u)(7+4u))}{\sum_{n=0}^{\infty}\mathbb{P}(n|\bar{n})(1+3u+2u^2)} \\
&= \frac{1+\bar{n}(1+u)^2(1+2u)+u(1+u)(7+4u)}{(1+u)(1+2u)}\,.
\end{aligned}
\tag{30}
$$

Finally, we can calculate

$$
\begin{aligned}
\Delta N_{\text{Pu},1}^{\text{Off}} &= N_{\text{Pu},1}^{\text{Off}} - N_{\text{Pu},0}^{\text{Off}} \\
&= \frac{\exp(\bar{n})(\bar{n}(1+4u)(1+\bar{n}(1+u))-4u)-\bar{n}+4u}{\exp(\bar{n})(\bar{n}+4u(\bar{n}-1))-\bar{n}+4u} - \frac{1+\bar{n}(1+u)^2(1+2u)+u(1+u)(7+4u)}{(1+u)(1+2u)}\,.
\end{aligned}
\tag{31}
$$

This can be approximated with

$$
\Delta N_{\text{Pu},1}^{\text{Off}} \approx \frac{\bar{n}}{\exp(\bar{n})-1} \tag{32}
$$

in the limit of small u.

Under public replication, there is only a benefit associated with the duplication event if the offspring transposon is itself a mutant. The probability that the offspring is a mutant is equal to R, the relatedness of the parent to the randomly-selected transposon within its genome that it copies to produce the offspring. This is

$$
\begin{aligned}
R &= \frac{\sum_{n=0}^{\infty}\sum_{m=1}^{\infty} f_{n,m} m \,\frac{m}{n+m}}{\sum_{n=0}^{\infty}\sum_{m=1}^{\infty} f_{n,m} m} \\
&\approx \frac{\sum_{n=0}^{\infty}\mathbb{P}(n|\bar{n})\sum_{m=1}^{\infty}\mathbb{Q}(m|u)m\,\frac{m}{n+m}}{\sum_{n=0}^{\infty}\mathbb{P}(n|\bar{n})\sum_{m=1}^{\infty}\mathbb{Q}(m|u)m}\,,
\end{aligned}
\tag{33}
$$

where $f_{n,m}m \approx \mathbb{P}(n|\bar{n})\mathbb{Q}(m|u)m$ is proportional to the probability that the parent resides in a host with n wild-type transposons and m mutant transposons at birth and $\frac{m}{n+m}$ is the relatedness between the parent and a randomly-selected transposon within its genome.

Again, since the inner sum is difficult to work with, we use the first two terms, giving:

$$
\begin{aligned}
R &\approx \frac{\sum_{n=0}^{\infty} \mathbb{P}(n|\bar{n}) \left((1-u)\frac{1}{n+1} + (1-u)u\frac{4}{n+2} + \mathcal{O}(u^2)\right)}{\sum_{n=0}^{\infty} \mathbb{P}(n|\bar{n}) \left((1-u) + 2(1-u)u + \mathcal{O}(u^2)\right)} \\
&\approx \frac{\sum_{n=0}^{\infty} \mathbb{P}(n|\bar{n}) \left(\frac{(n+2)+4u(n+1)}{(n+1)(n+2)}\right)}{1+2u} \\
&= \frac{\bar{n}^{-2} \sum_{n=2}^{\infty} \mathbb{P}(n|\bar{n}) \left(n(1+4u) - 4u\right)}{1+2u} \\
&= \frac{1 - \exp(-\bar{n}) + 4u(\bar{n} - 1 + \exp(-\bar{n}))/\bar{n}}{\bar{n} + 2u\bar{n}}\,,
\end{aligned} \tag{34}
$$

which can be approximated with

$$
R \approx \frac{1 - \exp(-\bar{n})}{\bar{n}} \tag{35}
$$

in the limit of small u.

2.5 Cost: Initial difference ΔN_1 for the parent transposon

The cost of the duplication event, which is incurred by any mutant transposons in the host genome where the offspring transposon is made, results from the reduction in host fitness caused by the offspring. The cost is the same under private and public replication.

We begin by calculating the experienced transposon number for a randomly-selected mutant, other than the offspring, that shares the offspring's genome—i.e. either the parent or a duplicate of the parent which is not the offspring—at the moment after the offspring's creation, N_1^{Par}.

$$
N_1^{\mathrm{Par}} = \frac{\sum_{n=0}^{\infty} \sum_{m=1}^{\infty} f_{n,m} \sum_{i=0}^{\infty} \mathbb{P}(i|nu) \sum_{j=0}^{\infty} \mathbb{P}(j|mu)\; m\; (m+j)\; (n+m+i+j+1)}{\sum_{n=0}^{\infty} \sum_{m=1}^{\infty} f_{n,m} \sum_{i=0}^{\infty} \mathbb{P}(i|nu) \sum_{j=0}^{\infty} \mathbb{P}(j|mu)\; m\; (m+j)}\,. \tag{36}
$$

Above, $f_{n,m}\, \mathbb{P}(i|nu)\, \mathbb{P}(j|mu)\, m$ is proportional to the probability that the randomly-selected parent for the duplication event resides in a type n, m, i, j host; $(m+j)$ is the number of mutant transposons which reside in that genome and hence will suffer the cost of the duplication event; and $n + m + i + j + 1$ is the transposon number experienced by any of those mutants.

The calculation proceeds:

$$
\begin{aligned}
N_1^{\text{Par}} &= \frac{\sum_{n=0}^{\infty}\sum_{m=1}^{\infty} f_{n,m}\sum_{i=0}^{\infty}\mathbb{P}(i|nu)\sum_{j=0}^{\infty}\mathbb{P}(j|mu)\ (m^2(n{+}m{+}i{+}j{+}1)+mj(n{+}m{+}i{+}1)+mj^2)}{\sum_{n=0}^{\infty}\sum_{m=1}^{\infty} f_{n,m}\sum_{i=0}^{\infty}\mathbb{P}(i|nu)\sum_{j=0}^{\infty}\mathbb{P}(j|mu)\ (m^2+mj)} \\
&= \frac{\sum_{n=0}^{\infty}\sum_{m=1}^{\infty} f_{n,m}\sum_{i=0}^{\infty}\mathbb{P}(i|nu)\ (m^3(1+u)^2+m^2(1+u)(n+i+1)+m^2u)}{\sum_{n=0}^{\infty}\sum_{m=1}^{\infty} f_{n,m}\sum_{i=0}^{\infty}\mathbb{P}(i|nu)\ (m^2(1+u))} \\
&= \frac{\sum_{n=0}^{\infty}\sum_{m=1}^{\infty} f_{n,m}\ (m^3(1+u)^2+m^2(1+u)(n(1+u)+1)+m^2u)}{\sum_{n=0}^{\infty}\sum_{m=1}^{\infty} f_{n,m}\ (m^2(1+u))} \\
&\approx \frac{\sum_{n=0}^{\infty} a_n\sum_{m=1}^{\infty}\mathbb{Q}(m|u)(m^3(1+u)^2+m^2(1+u)(n(1+u)+1)+m^2u)}{\sum_{n=0}^{\infty} a_n\sum_{m=1}^{\infty}\mathbb{Q}(m|u)(m^2(1+u))} \\
&= \frac{\sum_{n=0}^{\infty} a_n\left(\frac{1+4u+u^2}{(1-u)^3}(1+u)^2+\frac{(1+u)^2}{(1-u)^2}(n(1+u)+1)+\frac{u(1+u)}{(1-u)^2}\right)}{\sum_{n=0}^{\infty} a_n\left(\frac{(1+u)^2}{(1-u)^2}\right)} \\
&= \frac{\sum_{n=0}^{\infty} a_n\left(\frac{1+4u+u^2}{1-u}+n(1+u)+1+\frac{u}{1+u}\right)}{\sum_{n=0}^{\infty} a_n} \\
&= \frac{\bar{n}(1-u^2)+(1+u)^2+1+u+u\frac{1-u}{1+u}}{1-u}\,.
\end{aligned}
\tag{37}
$$

This allows us to calculate ΔN_1^{Par} as

$$
\begin{aligned}
\Delta N_1^{\text{Par}} &= N_1^{\text{Par}} - N_0 \\
&= \frac{\bar{n}(1-u^2)+(1+u)^2+1+u+u\frac{1-u}{1+u}}{1-u} - \frac{\bar{n}(1-u^2)+(1+u)^2+u\frac{1-u}{1+u}}{1-u} \\
&= \frac{1+u}{1-u}\,.
\end{aligned}
\tag{38}
$$

Step 2 of the invasion analysis (Fig. S2I) is now complete.

2.6 Calculating subsequent differences

Having calculated initial differences in experienced transposon number for the parent (ΔN_1^{Par}) and offspring ($\Delta N_{\text{Pr},1}^{\text{Off}}$, $\Delta N_{\text{Pu},1}^{\text{Off}}$), we now need to calculate how these differences dissipate over generations. This is equivalent to asking: for any pair of transposons in the same genome, how many generations, on average, will it take to split them up? We derive this result as follows.

Consider a single "focal" transposon. In generation t, suppose that there is a small probability δ that the focal transposon is associated with another transposon, its "pair". Then the experienced transposon number, where we are considering only the focal transposon and, potentially, its pair, is

$$
N_t(\delta) = \frac{\sum_{s=0}^{1}\mathbb{B}(s|1,\delta)(1+s)\ (1+s)}{\sum_{s=0}^{1}\mathbb{B}(s|1,\delta)(1+s)}\,. \tag{39}
$$

This gives

$$
\begin{aligned}
N_t(\delta) &= \frac{\sum_{s=0}^{1} \mathbb{B}(s|1,\delta)(1+2s+s^2)}{\sum_{s=0}^{1} \mathbb{B}(s|1,\delta)(1+s)} \\
&= \frac{1+2\delta+\delta(1-\delta)+\delta^2}{1+\delta} \\
&= \frac{1+3\delta}{1+\delta}\,, \qquad (40)
\end{aligned}
$$

where we have used the moments of the binomial distribution $\sum_k \mathbb{B}(k|n,p)k = np$ and $\sum_k \mathbb{B}(k|n,p)k^2 = np(1-p)+n^2p^2$.

In generation $t+1$, we have gone through a selection phase, a recombination phase, and a proliferation phase, which makes the experienced transposon number, considering only the focal transposon and its potential pair,

$$
N_{t+1}(\delta) = \frac{\sum_{s=0}^{1} \mathbb{B}(s|1,\delta)\, e^{\beta s} \sum_{k=0}^{s+1} \mathbb{B}(k|s+1,\frac{1}{2}) \sum_{\ell=0}^{\infty} \mathbb{P}(\ell|ku)(k+\ell)\ (k+\ell)}{\sum_{s=0}^{1} \mathbb{B}(s|1,\delta)\, e^{\beta s} \sum_{k=0}^{s+1} \mathbb{B}(k|s+1,\frac{1}{2}) \sum_{\ell=0}^{\infty} \mathbb{P}(\ell|ku)(k+\ell)}\,, \qquad (41)
$$

where

$$
\beta = \beta_{wn}/\bar{w} \qquad (42)
$$

is the slope of host relative fitness on transposon number. This gives

$$
\begin{aligned}
N_{t+1}(\delta) &= \frac{\sum_{s=0}^{1} \mathbb{B}(s|1,\delta)\, e^{\beta s} \sum_{k=0}^{s+1} \mathbb{B}(k|s+1,\frac{1}{2}) \sum_{\ell=0}^{\infty} \mathbb{P}(\ell|ku)(k^2+2k\ell+\ell^2)}{\sum_{s=0}^{1} \mathbb{B}(s|1,\delta)\, e^{\beta s} \sum_{k=0}^{s+1} \mathbb{B}(k|s+1,\frac{1}{2}) \sum_{\ell=0}^{\infty} \mathbb{P}(\ell|ku)(k+\ell)} \\
&= \frac{\sum_{s=0}^{1} \mathbb{B}(s|1,\delta)\, e^{\beta s} \sum_{k=0}^{s+1} \mathbb{B}(k|s+1,\frac{1}{2})\,(k^2(1+2u+u^2)+ku)}{\sum_{s=0}^{1} \mathbb{B}(s|1,\delta)\, e^{\beta s} \sum_{k=0}^{s+1} \mathbb{B}(k|s+1,\frac{1}{2})\,(k(1+u))} \\
&= \frac{\sum_{s=0}^{1} \mathbb{B}(s|1,\delta)\, e^{\beta s} \left((\frac{s+1}{4} + \frac{(s+1)^2}{4})(1+u)^2 + \frac{s+1}{2}u \right)}{\sum_{s=0}^{1} \mathbb{B}(s|1,\delta)\, e^{\beta s} \left(\frac{s+1}{2}(1+u) \right)} \\
&= \frac{\sum_{s=0}^{1} \mathbb{B}(s|1,\delta)\, e^{\beta s}\,(s^2(1+u)^2 + s(3+8u+3u^2)+2+6u+2u^2)}{\sum_{s=0}^{1} \mathbb{B}(s|1,\delta)\, e^{\beta s}\,(s(2+2u)+2+2u)} \\
&= \frac{(1-\delta)(1+3u+u^2)+e^{\beta}\delta(3+8u+3u^2)}{(1-\delta)(1+u)+e^{\beta}\delta(2+2u)}\,. \qquad (43)
\end{aligned}
$$

In generation t, the increase in experienced transposon number that results from some small association δ is:

$$
\begin{aligned}
\Delta N_t(\delta) &= N_t(\delta) - N_t(0) \\
&= \frac{1+3\delta}{1+\delta} - 1 \\
&= \frac{2\delta}{1+\delta}\,. \qquad (44)
\end{aligned}
$$

And in generation $t+1$, the increase in experienced transposon number that remains after the small association δ from generation t is:

$$\begin{aligned}\Delta N_{t+1}(\delta) &= N_{t+1}(\delta) - N_{t+1}(0) \\ &= \frac{(1-\delta)(1+3u+u^2) + e^{\beta}\delta(3+8u+3u^2)}{(1-\delta)(1+u) + e^{\beta}\delta(2+2u)} - \frac{1+3u+u^2}{1+u} \\ &= \frac{e^{\beta}\delta(1+u)}{1+\delta(2e^{\beta}-1)}\,. \end{aligned} \tag{45}$$

Accordingly, the change in a small 'excess' in experienced transposon number from one generation to the next is given by

$$\begin{aligned}\lim_{\delta\to 0}\frac{\Delta N_{t+1}(\delta)}{\Delta N_t(\delta)} &= \lim_{\delta\to 0}\frac{(1+\delta)e^{\beta}\delta(1+u)}{2\delta(1+\delta(2e^{\beta}-1))} \\ &= \frac{e^{\beta}(1+u)}{2}\,. \end{aligned} \tag{46}$$

This is the probability that, over any single generation, a pair of associated transposons is 'kept together' given the combined effects of selection, transposon duplication, and recombination. Here, the factor e^{β} (a number typically slightly less than one) accounts for selection against genomes with more transposons eliminating the pair of transposons; the factor of $1/2$ comes from the 50% chance that recombination will split up the pair in any given generation; and the factor of $1+u$ comes from the probability that a new copy of either transposon is added to the genome, which increases the odds that the original pair (or their direct descendants) remain associated. This suggests that the average number of generations that a given pair of transposons remains associated is $2/(2-e^{\beta}(1+u))$.

Given an initial difference in experienced transposon number ΔN_1, we will calculate subsequent differences ΔN_2, ΔN_3, *etc.* using

$$\Delta N_{t>1} \approx \Delta N_1 \left(\frac{e^{\beta}(1+u)}{2}\right)^{t-1}\,. \tag{47}$$

This completes step 3 of the invasion analysis (Fig. S2I).

2.7 Accuracy of approximations

The closed-form approximations for $\Delta N^{\mathrm{Off}}_{\mathrm{Pr},1}$, $\Delta N^{\mathrm{Off}}_{\mathrm{Pu},1}$, and $\Delta N^{\mathrm{Par}}_{1}$ that we derive in equations 27, 31, and 38 are central to the invasion analysis. Therefore, it is important to check how much error we introduced in making these approximations.

In Fig. S4, for a range of parameter values, we plot the quantities N_0, $\Delta N^{\mathrm{Off}}_{\mathrm{Pr},1}$, $\Delta N^{\mathrm{Off}}_{\mathrm{Pu},1}$, and $\Delta N^{\mathrm{Par}}_{1}$, computed in three different ways. First, as computed by fully evaluating the sums across the full joint distribution $f_{n,m}$ of host genotypes, as measured from actual simulations of the two-type deterministic model. Second, we plot the same quantities as calculated over the marginalized distribution

$$f^{\mathrm{Marg}}_{n,m} = \left(\sum_j f_{n,j}\right)\left(\sum_i f_{i,m}\right)\,, \tag{48}$$

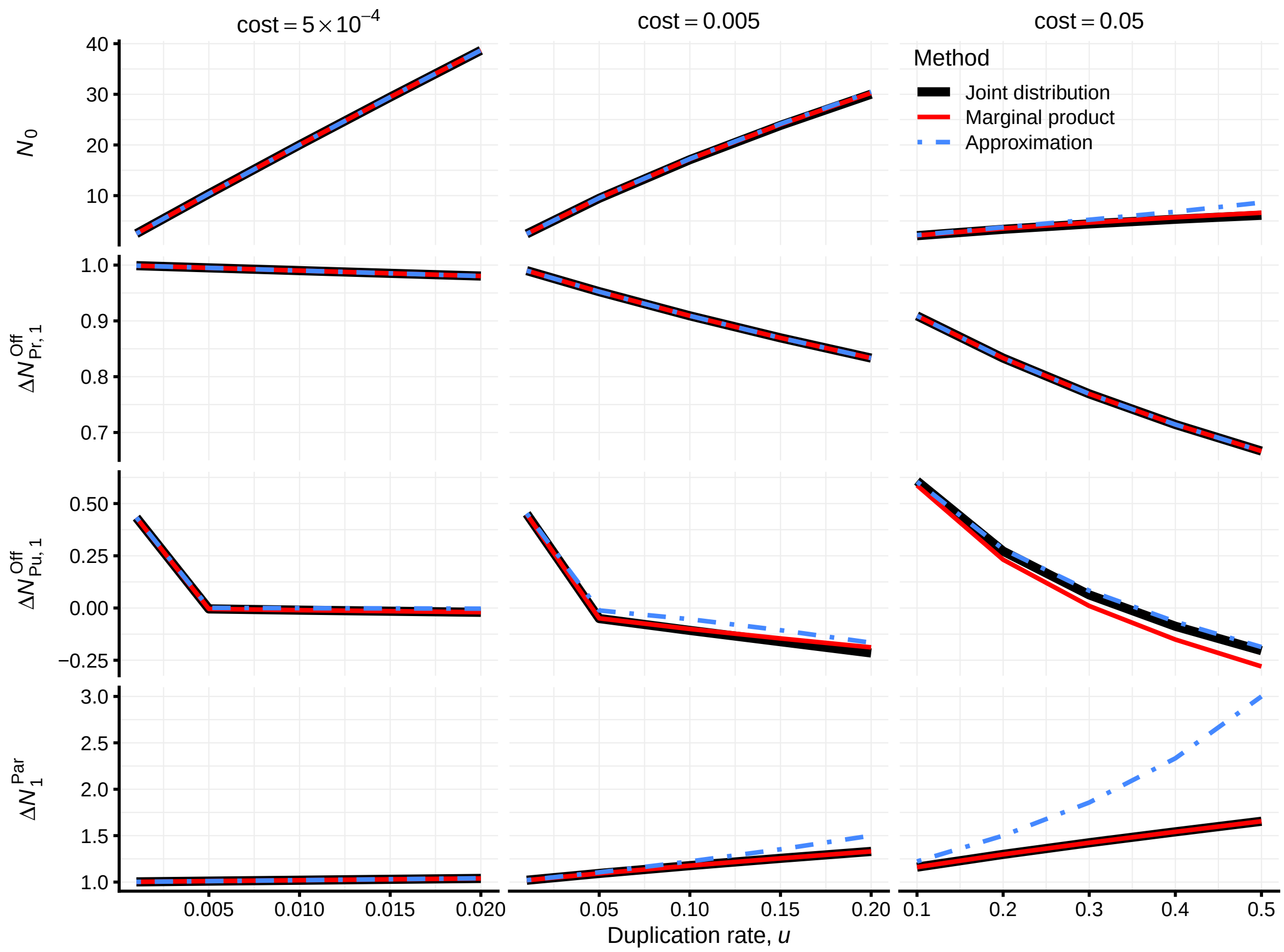

Figure S4: Assessing the impact of approximations made in deriving ΔN_1s for the invasion analysis. We show four key quantities for the invasion analysis (N_0, $\Delta N^{\text{Off}}_{\text{Pr},1}$, $\Delta N^{\text{Off}}_{\text{Pu},1}$, and ΔN^{Par}_1) and their correct values (thick black lines) based on numerically solving equations 20, 24, 28, and 36, respectively, using the full joint distribution $f_{n,m}$ of genotypes directly from deterministic simulations. This is contrasted with solutions obtained by approximating $f_{n,m}$ with the marginal product distribution $a_n b_m$ (thin red lines) to show that the error introduced by using a marginal product distribution is relatively small. Finally, we also show closed-form solutions (equations 22, 27, 31, 38) using the Poisson and quasigeometric approximations for the distribution of wild-type and mutant transposons, respectively, in the invasion analysis (dashed blue lines). The overall message is that the approximations which are brought together in section 2.8 are generally good, particularly when u is small; when u is large (around $u > 0.1$), the approximations for ΔN^{Par}_1, in particular, can be poor, and this is because of the use of the quasigeometric distribution as an approximation for the distribution of the number of mutant transposons across genomes. Results are plotted for $u \in \{0.001, 0.005, 0.01, 0.015, 0.02\}$ for cost $c = 5 \times 10^{-4}$; $u \in \{0.01, 0.05, 0.1, 0.15, 0.2\}$ for cost $c = 0.005$; and $u \in \{0.1, 0.2, 0.3, 0.4, 0.5\}$ for cost $c = 0.05$, using the fitness function $w(n) = (1-c)^{n^2/2}$.

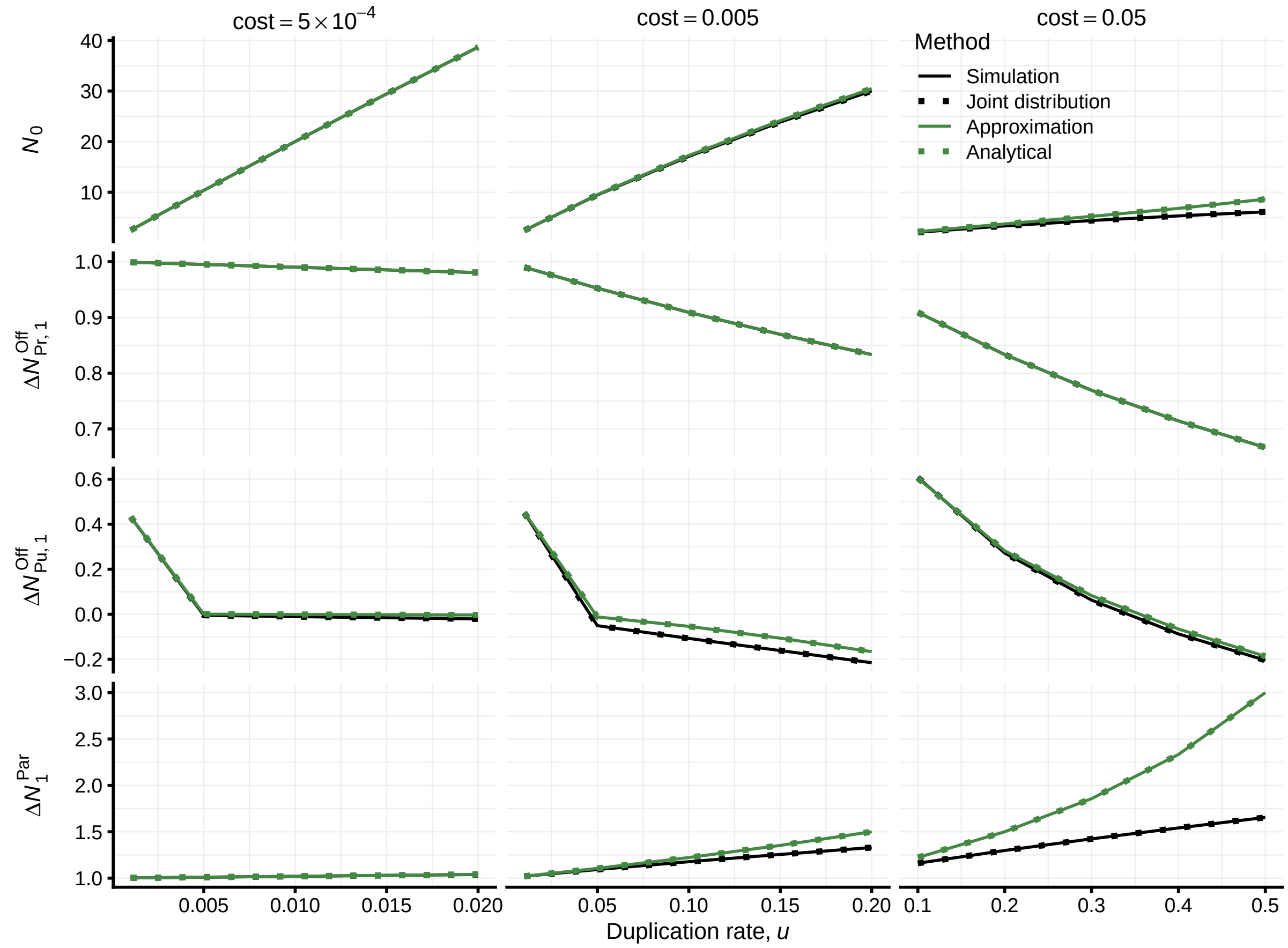

Figure S5: Assessing the accuracy of calculations in this paper. Above, the straight black lines and dotted black lines represent, respectively, the quantities N_0, $\Delta N^{\mathrm{Off}}_{\mathrm{Pr},1}$, $\Delta N^{\mathrm{Off}}_{\mathrm{Pu},1}$, and $\Delta N^{\mathrm{Par}}_{1}$ as directly measured within simulations of the two-type deterministic model and the same quantities evaluated using the sums written out in equations 20, 24, 28, and 36. They overlap, supporting that the statement of the sums in equations 20, 24, 28, and 36 is correct. The straight green lines and dotted green lines represent, respectively, the same four quantities evaluated again using the sums written out in equations 20, 24, 28, and 36, but substituting the $\mathbb{P}$ and $\mathbb{Q}$ distributions for $f_{n,m}$, and then the simpler closed-form approximations in equations 22, 27, 31, 38. They overlap as well, showing that the derivation of the closed-form approximations was successful. The divergence between the black and green lines reflects what is shown above in Fig. S4.

and finally, using the approximations for the four quantities in equations 22, 27, 31, and 38. This shows that the approximations are generally good, with the exception of the approximation for ΔN_1^{Par} when the duplication rate u is large (roughly, when $u > 0.1$).

The majority of the error shown in Fig. S4 is due to the mismatch between the true marginal distribution of mutant transposons and the quasigeometric distribution we use to approximate it, when u is large. Therefore, improving this approximation would probably increase the accuracy of our calculations substantially.

Looking back to Fig. S3C, the lack of accuracy in the quasigeometric approximation arises primarily because of the effect of selection. We have found that using $u(1-u)$ instead of u as the parameter of the quasigeometric distribution (i.e. $b_m \approx \mathbb{Q}(m|u(1-u))$ instead of $b_m \approx \mathbb{Q}(m|u)$ appears to lead to better approximations. However, it is not clear whether this improvement depends upon the exact form of the fitness function $w(n)$, so we have not pursued this any further.

Finally, Fig. S5 simply verifies that the quantities N_0, $\Delta N_{\text{Pr},1}^{\text{Off}}$, $\Delta N_{\text{Pu},1}^{\text{Off}}$, and ΔN_1^{Par} have been calculated correctly.

2.8 Putting it all together

Natural selection favours a small increase to the transposon duplication rate if

$$rB > C\,, \tag{49}$$

where the relatedness r is equal to 1 under private replication and R (equations 33–35) under public replication, B is the benefit of an extra duplication and C is the cost of an extra duplication. We shall choose to express B as the additional number of mutant transposons generated because of the duplication event, and C as the number of mutant transposons lost because of the duplication event, where the gains and losses are measured after the proliferation stage of the model. The choice to focus on gains and losses measured after the proliferation stage in particular is arbitrary, but slightly simplifies calculations.

The benefit B is

$$B = \prod_{t=1}^{\infty} \frac{W_t^{\text{Off}}}{W_0}\,, \tag{50}$$

where W_t^{Off} is the experienced relative fitness of an offspring transposon during the tth generation of the duplication event (section 2.4) and W_0 (equations 18, 19) is the experienced fitness of a randomly-selected transposon after proliferation.

We can approximate W_t^{Off} with

$$W_t^{\text{Off}} \approx W_0 + \Delta N_t^{\text{Off}} \beta\,, \tag{51}$$

where ΔN_t^{Off} is $\Delta N_{\text{Pr},t}^{\text{Off}}$ (equations 27, 47) under private replication and $\Delta N_{\text{Pu},t}^{\text{Off}}$ (equations 31, 47) under public replication and β (equation 42) is the least-squares linear regression slope of host relative fitness on transposon number.

Filling in values from equations 18, 27, 31, and 47, this yields

$$B \approx \prod_{t=1}^{\infty} \left(1 + \beta \left(\frac{(1+u)e^{\beta}}{2}\right)^{t-1}\right) \tag{52}$$

under private replication and

$$B \approx \prod_{t=1}^{\infty} \left(1 + \beta(1+u) \left(\frac{\exp(\bar{n})(\bar{n}(1+4u)(1+\bar{n}(1+u)) - 4u) - \bar{n} + 4u}{\exp(\bar{n})(\bar{n} + 4u(\bar{n}-1)) - \bar{n} + 4u} - \frac{1 + \bar{n}(1+u)^2(1+2u) + u(1+u)(7+4u)}{(1+u)(1+2u)}\right) \left(\frac{(1+u)e^{\beta}}{2}\right)^{t-1}\right) \quad (53)$$

under public replication.

The cost C is

$$C = 1 - \prod_{t=1}^{\infty} \frac{W_t^{\mathrm{Par}}}{W_0}\,, \quad (54)$$

where W_t^{Par} is the experienced fitness of a parent transposon during the tth generation of the duplication event.

As above, we approximate W_t^{Par} with

$$W_t^{\mathrm{Par}} \approx W_0 + \Delta N_t^{\mathrm{Par}} \beta\,, \quad (55)$$

with $\Delta N_t^{\mathrm{Par}}$ as defined in equations 38 and 47.

This yields

$$C \approx 1 - \prod_{t=1}^{\infty} \left(1 + \beta \frac{(1+u)^2}{1-u} \left(\frac{(1+u)e^{\beta}}{2}\right)^{t-1}\right) \quad (56)$$

under both public and private replication.

While equations 52 and 53 for B, equation 56 for C, the appropriate relatedness term can be substituted into the inequality $rB > C$ to yield a condition for natural selection to favour a small increase in the duplication rate, the expressions resulting are rather complex. By taking only the leading terms in these definitions, we can use the much simpler approximations

$$\begin{aligned} r &= \begin{cases} 1 & \text{private replication} \\ 1/\bar{n} & \text{public replication} \end{cases} \\ B &= 1 \\ C &= 2u \end{aligned} \quad (57)$$

in place of the more complex expressions. Here, the approximations for r and B captures that the benefit of an additional duplication to the mutant lineage is one extra transposon, which has probability r of being a mutant; in practice B is not exactly equal to 1 because of transient selection effects, but this is a relatively small effect (Fig. S6). The approximation $C = 2u$ captures that the duplication event associates the mutant parent with an extra transposon for an average of around 2 generations, and the selective disadvantage of an additional transposon, β, is approximately equal to $-u$ at equilibrium, since (as emphasized by Charlesworth and Charlesworth [1]) at this point we have $\beta \approx \frac{\partial \ln w(\bar{n})}{\partial \bar{n}} \approx -(u - v)$.

These approximations allow us to express the conditions for natural selection to favour a small increase in the duplication rate as approximately

$$u < \frac{1}{2} \quad (58)$$

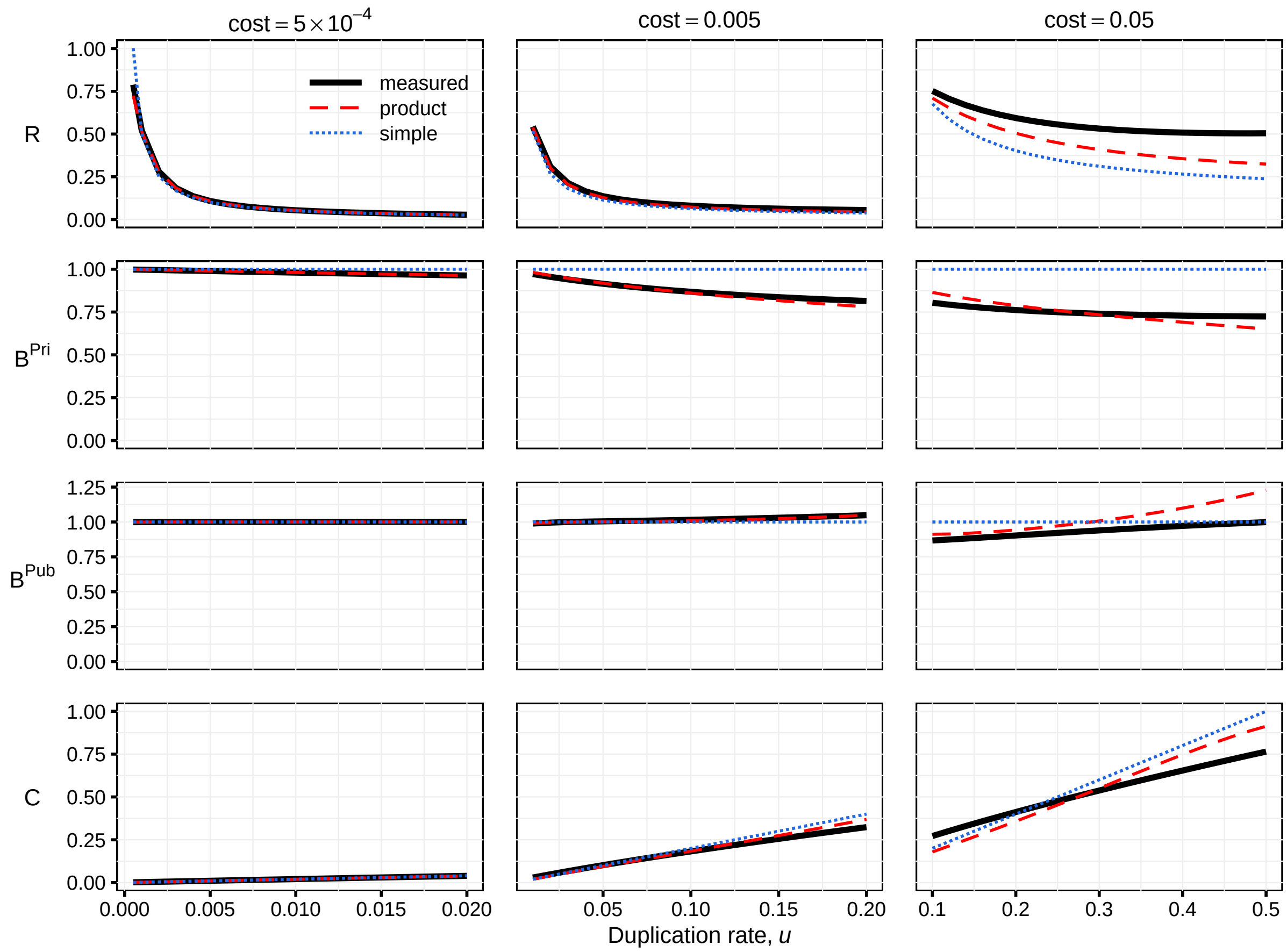

Figure S6: Comparison of quantities R, B (for both private and public replication) and C as measured directly in the simulations (solid black lines) as compared to (1) expressions in equations 34, 52, 53, and 56 ("product") versus (2) simplified expressions in equation 57 ("simple").

under private replication and

$$u < \frac{1}{2\bar{n}} \tag{59}$$

under public replication.

In Fig. S6, we compare the quantities r, B, and C measured in three different ways: directly from the simulations, using the approximations with infinite products shown above (i.e. equations 34, 52, 53, and 56), and using the "simple" closed-form versions in equation 57. This shows that, in general, the "infinite product" approximations are relatively close to the measured values, while the "simple" versions are also quite close.

3 Host fitness models

Natural selection against transposon activity—and, hence, the outcome of transposon–host coevolution—depends crucially on how transposons harm their hosts. However, the difficulty of measuring transposon-mediated effects on host fitness means that it is uncertain which mechanisms of harm are actually responsible for selection against transposon proliferation. Here, we investigate six hypotheses for selection against transposons, parameterizing a host fitness function for each using published data. In constructing these fitness functions, our goal is to arrive at plausible cost estimates. We concentrate on estimates from *Drosophila melanogaster*, because this is the best-studied model organism for transposon proliferation in animals.

Three of the selective forces invoked depend primarily on transposon copy number: the risk of ectopic recombination, insertions into genes or regulatory regions, and the energetic burden of synthesizing transposon DNA. The three remaining selective forces depend primarily on overall transposon activity, the product of copy number and average expression level: the energetic cost of transposon expression, the cellular cost of massive overexpression of identical proteins, and the production of double-strand breaks by DNA transposons.

3.1 Ectopic recombination

Because transposons are homologous sequences dispersed throughout the genome, two transposons of the same family at heterologous loci may undergo ectopic exchange. If this occurs during meiosis, crossing over between nonhomologous loci may occur, resulting in massive gene loss or duplication in gametes, rendering them inviable [5–7]. Ectopic recombination may also occur during mitosis, but since the frequency of mitotic recombination is much lower than that of meiotic recombination—for example, 10^4–10^5-fold lower in *Saccharomyces cerevisiae* [8]—we ignore this possibility here. Ectopic exchange between transposons has been directly observed in yeast, *D. melanogaster*, and humans [7]. Because the risk of ectopic recombination scales quadratically with the number of dispersed homologous sequences, this cost naturally increases faster than log-linearly with transposon number. Consequently, it has traditionally been considered to be the most important transposon-related cost to host fitness [7].

If each pair of dispersed transposons is equally likely to cause an ectopic recombination event, and host fitness scales with the proportion of viable gametes produced, we can use a fitness function

$$w = \left(1 - \frac{\lambda\varepsilon\rho}{100}\right)^{\frac{1}{2}n(n-1)-n_{\mathrm{g}}}, \tag{60}$$

where λ is the length of the transposon in Mb, ε is the efficiency of ectopic recombination relative to allelic recombination, ρ is the allelic rate of recombination in cM / Mb, the denominator 100 converts centiMorgans to Morgans, n is transposon copy number, and n_{g} is the number of transposon copies that are homozygous, making $\frac{1}{2}n(n-1) - n_{\mathrm{g}}$ the number of possible pairs of dispersed transposons. To summarize, the term $\frac{\lambda\varepsilon\rho}{100}$ is the probability that any given pair of transposons results in an ectopic recombination event, which we assume is fatal to the gamete. Therefore, the overall proportion of gametes that survive the risk of

ectopic recombination is $1-\frac{\lambda\varepsilon\rho}{100}$ raised to the power of the total number of pairs of transposons in the genome.

We take $\lambda = 0.005$Mb as a representative transposon length, as RNA transposons in *D. melanogaster* are generally 3–7 kb in length [9]. For the recombination rate between dispersed elements, studies in yeast suggest that ectopic recombination is approximately 15% as frequent as allelic recombination, although the rate can be elevated if the ectopic sequences occur very close together (within ∼300 kb) on homologous chromosomes [10]. The allelic recombination rate is approximately 1 cM/Mb in *Drosophila* [11, 12] and humans [13]. More generally, the median recombination rate in mammals [14] and plants [15] is approximately 1 cM/Mb. Overall, then, we adopt $\rho = 1$ cM/Mb and $\varepsilon = 0.15$ as an estimate of the rate and relative efficiency of ectopic recombination. Silencing of transposons may result in their adopting a heterochromatic state, and the rate of recombination in heterochromatin is generally lower than that in euchromatin. However, for the sake of simplicity, we ignore the possibility of suppression directly tempering the copy number cost of transposons.

3.2 Deleterious insertions

To model the insertional effects of transposons, at least three aspects need to be considered. First, the effect of individual insertions; second, the relative magnitude of heterozygous versus homozygous effects; and third, interactions between insertions. Taken together, this could be modelled as an effect for each homozygous insertion, a lesser effect for each heterozygous insertion, and synergistic effects for each possible pair of insertions (of three possible types: homozygous–homozygous, homozygous–heterozygous, and heterozygous–heterozygous). Together, this yields a fitness function of the form

$$w = (1-s)^{\frac{n_\mathrm{g}}{2}}(1-Hs)^{n_\mathrm{h}}(1-ts)^{\frac{1}{8}n_\mathrm{g}(n_\mathrm{g}-2)}(1-Hts)^{\frac{1}{2}n_\mathrm{g}n_\mathrm{h}}(1-H^2ts)^{\frac{1}{2}n_\mathrm{h}(n_\mathrm{h}-1)}\,. \qquad (61)$$

Above, n_g and n_h are the number of homozygous and heterozygous insertions, respectively; s is the average effect of a homozygous insertion; H is the relative effect of a heterozygous insertion compared to a homozygous insertion; and t is the relative epistatic effect for each pair of homozygous insertions. The five terms multiplied together on the right hand side of the above equation represent costs owing to each of the five kinds of effects (homozygous insertions, heterozygous insertions, and three types of interactions between pairs of insertions).

Effects of deleterious mutations are commonly expressed using an exponential function of the number of mutations and the number of mutations squared (e.g., of the form $w = \exp(-\alpha n - \beta n^2)$). For an expression of this form, when s is small, the above function is approximately equal to

$$w = \exp(-\alpha_\mathrm{g}n_\mathrm{g} - \alpha_\mathrm{h}n_\mathrm{h} - \beta_\mathrm{gg}n_\mathrm{g}^2 - \beta_\mathrm{gh}n_\mathrm{g}n_\mathrm{h} - \beta_\mathrm{hh}n_\mathrm{h}^2)\,, \qquad (62)$$

where $\alpha_\mathrm{g} = \frac{s}{2}(1-\frac{t}{2})$, $\alpha_\mathrm{h} = Hs(1-\frac{Ht}{2})$, $\beta_\mathrm{gg} = \frac{ts}{2}$, $\beta_\mathrm{gh} = \frac{Hts}{2}$ and $\beta_\mathrm{hh} = \frac{H^2ts}{2}$.

Several studies have estimated the fitness effects of homozygous or hemizygous transposon insertions. Eanes et al. [16], investigating *P* element insertions on the X chromosome in *D. melanogaster* males, estimated an average fitness cost of 1.1% per hemizygous insertion. Houle and Nuzhdin [17], studying *copia* insertions in *D. melanogaster* females, estimated an average offspring viability cost of 0.76% per homozygous insertion. Costs of a larger

magnitude were estimated by Mackay et al. [18], who studied the insertional effects of a modified *P* element, estimating an average viability cost of 12.2% per homozygous insertion. However, Houle and Nuzhdin [17] note that the applicability of the estimates made by Mackay et al. [18] may be limited because the modified *P* element used was four times longer than the natural element and may have differed in target-site specificity. They also note that mutational effects may have been overestimated due to the fact that aborted transpositions were undetectable [17].

To put these figures into a wider context, it is helpful to compare them to a study of mini-*Tn10* insertions in *E. coli* by Elena and Lenski [19], who estimated an average fitness cost of 2.73% per insertion. This is roughly consistent with the lower estimates for insertional effects above if an adjustment is made for the fact that only 48% of the *D. melanogaster* genome is genic, compared with nearly all of the *E. coli* genome [20]. Overall, therefore, an average insertional effect of $s \approx 1\%$ for a typical multicellular eukaryote seems appropriate for the purposes of our models.

Fitness effects in these experiments will not have been limited to insertional effects, but will also have included other effects of transposon insertions. These other costs will likely have been small relative to insertional effects. Ectopic recombination would not be a significant issue for *E. coli* and even if it may have occurred in the Eanes et al. *Drosophila* experiment [16]—recombination does not normally occur in *Drosophila* males, but it can be induced by *P* elements [21]—the maximum cost due to ectopic recombination of 10 homozygous insertions, for the 2,907-bp *P* element and a 1 cM/Mb rate of recombination, would yield a total fitness reduction of only 0.017%, 3 orders of magnitude below the measured effect for 10 insertions. Copy numbers were higher in the Houle and Nuzhdin study [17] (from 31–67 copies), which for the 5,146-bp copia element could have incurred up to a 1.6% total fitness cost due to ectopic recombination, or 0.047% per element over the range examined. Again, this is substantially lower than the estimated fitness effect per element. Estimates from Eanes et al. [16] and Elena and Lenski [19] may include the effects of double-strand breaks, as *P* and *Tn10* are DNA elements, but the Houle and Nuzhdin experiment [17] would not have, as *copia* is an RNA element. DNA synthesis costs could not have been significant in any of the studies (see below). Expression and/or misfolding costs may have played a role in the experiments of Eanes et al. [16] and of Houle and Nuzhdin [17]. However, their rough congruence with the data of Elena and Lenski [19] suggests overall that insertions are the main effect being measured here. By making this assumption, we are being conservative in that if other costs were at play, this would only reduce the costs caused by the insertions and thereby further reduce their ability to prevent the tragedy of the commons.

As for synergistic effects, the studies cited above do not support a statistically-significant synergistic effect of insertions. However, the study with the most data points was that by Elena and Lenski [19], which found a non-significant synergistic effect roughly equivalent to $t = 0.025$. We therefore include $t = 0.025$ to again be conservative and maximise the potential impacts of costs.

Finally, we are left with estimating the heterozygous effect of an insertion. Estimates for the average heterozygous effect of a mutation vary, but overall, $H = 0.1$ has been identified as a reasonable estimate for *D. melanogaster* [22]. The aforementioned Mackay et al. study [18] found an average heterozygous insertion effect of 5.5%, compared to the average homozygous insertion effect of 12.2%, which suggests an average heterozygous effect closer to $H = 0.45$.

However, caution over this latter estimate is warranted for the reasons of Houle and Nuzhdin cited above. In light of this, we adopt the parameters $s = 0.01$, $t = 0.025$, $H = 0.1$.

Although we adopt uniform parameters, insertional effects are likely to be highly variable between sites, depending on the target-site specificity of the transposon family as well as the ratio of coding to non-coding DNA in the genome. Because of this insertion-site dependency, insertions have naturally more variable effects than other costs; highly-deleterious insertions will be more strongly selected against than nearly-neutral insertions, resulting in an accumulation of transposons at sites with smaller fitness effects [7]. However, preliminary modelling with a two-site-class model of insertional effects (high cost and low cost) did not lead to significantly different qualitative outcomes (not shown). Therefore, we ignore this variation here for the sake of simplicity, only noting that increasing the variability among insertional effects will generally lead to a higher transposon number at equilibrium than a fixed insertional effect would suggest.

3.3 DNA synthesis

In most eukaryotes, a substantial fraction of the genome (20–80%) is transposon-derived. The fitness cost of duplicating this junk DNA comes down to the energetic cost of DNA synthesis relative to the cell's overall "energy budget". To quantify this cost, we used estimates for the bioenergetic cost of a gene from a study by Lynch and Marinov [23]. They estimated that the energy budget of a rapidly-growing cell is approximately $26.92V^{0.97} \times 10^9$ P, where the unit P represents ATP $\rightarrow$ ADP hydrolysis events, or 5.322×10^{13} P for a multicellular eukaryote with a cell volume of 2,500 μm^3 (the median of measured cell volumes; [23]). This implies a fitness cost, per ATP $\rightarrow$ ADP hydrolysis event, of 1.9×10^{-14} P^{-1}. The synthesis cost of DNA is 526 P per base [23], suggesting a fitness function of the form

$$w = 1 - Y\lambda n\,, \tag{63}$$

where λ is the length of the transposon in Mb and $Y \approx 1.0 \times 10^{-5}$ Mb^{-1} is the cost of synthesising 1 Mb of DNA (calculated as $1.9 \times 10^{-14}\ \text{P}^{-1} \times 526\ \text{P} \cdot \text{b}^{-1} \times 10^6\ \text{b}$). Note that, although collectively the cost of synthesising junk DNA can be large (for example, on the order of 1% for humans), the cost of synthesising any single transposon, with a typical length of 5 kb, is trivial.

3.4 Double-stranded breaks

The next possibility we consider applies to DNA transposons only. The cut-and-paste mechanism of DNA transposons leaves behind a double-strand break (DSB), which, if unrepaired, can imperil host fitness by leading to incorrect segregation of chromosomes during cell division. The magnitude of this risk is difficult to assess directly. A study by Rothkamm et al. [24] measured the survival of Chinese hamster ovary (CHO) cells following different levels (0–8 Gy) of ionizing radiation, which causes double-strand breaks. Under these conditions, 1 Gy of radiation induces approximately 30 DSBs [24]. The data of Rothkamm et al. [24] fit the function

$$w = \exp(-Bd - Cd^2)\,, \tag{64}$$

where d is the number of DSBs, $B = 2.32074 \times 10^{-3}$, and $C = 3.11734 \times 10^{-5}$ (Fig. S7A). Rothkamm et al. [24] administered the irradiation over a period of approximately 1 minute, which is much shorter than the generation time of CHO cells, ∼1 day. The majority of DSBs were repaired after 1 day. Based on this information, we make the assumption that this "acute" cost of DSBs can be interpreted as the number of DSBs per cell cycle which cause death. However, this is likely to significantly overestimate the strength of selection against double-strand breaks if cells can tolerate double-strand breaks spread over a longer period of time better than they can all at once. The number of breaks, d, is equal to $j\bar{u}n$, where $j \geq 1$ is the number of "cut-and-paste" transpositions per successful copy, and $\bar{u}n$ is the total activity of transposons. This value will vary depending on whether transposition occurs randomly with respect to the cell cycle, or whether transposons are adapted to transpose more frequently during the phases of the cell cycle in which transposition is more likely to result in a successful copy (i.e. after the replication fork has passed through the transposon's locus of origin, but not its destination locus). Based on the likelihood that there will be many cases where transposition is unsuccessful by this mechanism, we adopt $j = 10$ as an estimate.

3.5 Transcription and translation

In order to do the work of transposing, transposons need to be transcribed and translated. At high copy numbers and expression levels, these costs may become significant for the cell. As above, we assume that each unit of hydrolysed ATP carries a fitness cost of 1.9×10^{-14}. Lynch and Marinov [23] estimate that the cost of transcription in a higher eukaryote is

$$s_{\mathrm{RNA}} = 46 L_{\mathrm{r,mat}} N_{\mathrm{r}} \cdot 1.9 \times 10^{-14}\,, \tag{65}$$

where $L_{\mathrm{r,mat}}$ is the length of the mature RNA transcript—that is, the length of the coding sequence in bases, plus approximately 100 bases for the poly-A tail—and N_{r} is the number of transcripts.

Similarly, Lynch and Marinov estimate that translation carries a cost

$$s_{\mathrm{p}} = 24 L_{\mathrm{p}} N_{\mathrm{p}} \cdot 1.9 \times 10^{-14}\,, \tag{66}$$

where L_{p} is the length of the protein in amino acid residues, and N_{p} is the protein copy number [23].

Functional LTR-type RNA transposons assemble into virus-like particles (VLPs), which require the production of multiple protein subunits. For example, the *Ty1* retrotransposon in yeast, which is highly similar to *copia* in *D. melanogaster*, assembles into a ∼14.1 MDa VLP [26] consisting largely of a multi-copy capsid protein surrounding a core containing the template and reverse transcriptase. For an average mass of 110 Da per amino acid, this suggests that the production of approximately 128,000 amino acids is required for assembly of a functional transposon. Taken together, this yields an approximate cost for a *Ty1*/*copia*-like element of $c = 6 \times 10^{-8}$ per assembled particle. The number of VLPs required, on average, for one successful duplication, in the absence of any host suppression acting to disrupt VLPs, is denoted by k. This value is difficult to estimate. In *Saccharomyces cerevisiae*, multiple clusters of ∼10 VLPs can be imaged in wild-type cells [27], even though duplication rates for transposons in this species are on the order of one every thousand generations or lower. We

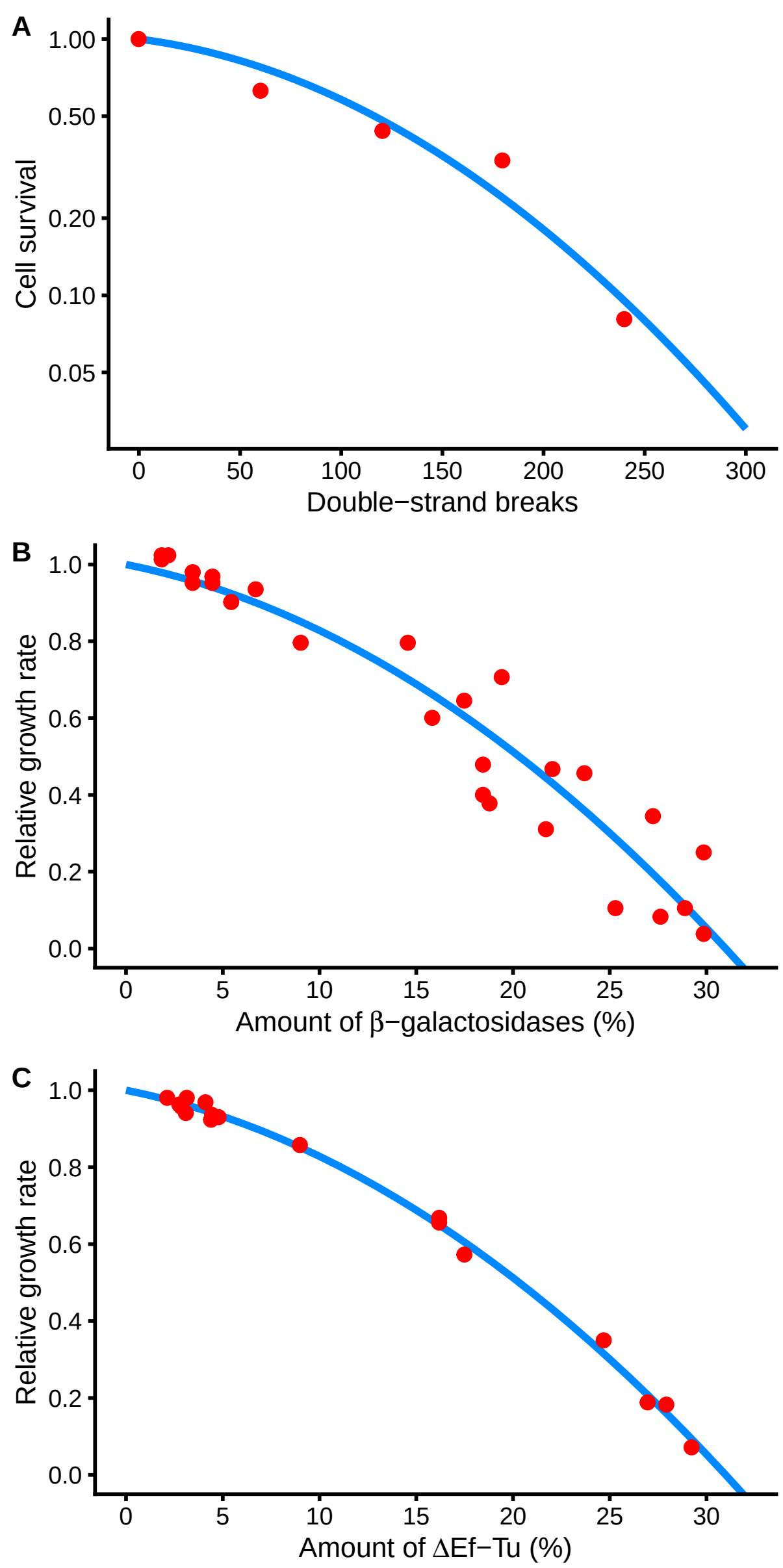

Figure S7: Data for unrepaired double-stranded breaks and protein aggregation fitness models. (A) Rothkamm et al. [24] on the impact of double-stranded breaks on cell fitness; (B, C) Dong et al. [25] on the impact of overexpression of proteins on cell fitness.

use $k = 10$ as our central estimate, but test values for k across several orders of magnitude in our simulations. The resulting fitness function is then

$$w = 1 - kc\bar{u}n\,, \tag{67}$$

where $\bar{u}n$ is the total activity of transposons.

For LINE-type RNA transposons, the number of amino acids required to produce a functional unit capable of retrotransposition is harder to assess. LINEs encode two proteins: ORF1p, a chaperone that binds along the length of the mRNA transcript, and ORF2p, which captures the poly-A tail of the mRNA transcript and performs reverse transcription [28]. The retrotransposition-capable ribonucleoprotein appears to consist of one mRNA transcript, one ORF2p, and several copies of the homotrimeric ORF1p. Exactly how many ORF1p are translated for each ORF2p is unclear, partly because ORF2p is expressed at such low levels that its cellular abundance has been difficult to measure [29].

Taylor et al. [28] attempted to quantify the ORF1p:ORF2p ratio in several different ways. One approach involved quantifying the relative cellular abundance of ORF1p versus ORF2p by using an artificial construct, *ORFeus*-Hs [30], which is based on the sequence for human *L1*, but optimized for higher expression via synonymous nucleotide changes. Expression of this construct produced approximately 1,000–10,000 ORF1p for every ORF2p. Taylor et al. further estimated that ORF1p production by *ORFeus*-Hs was ∼2.5-fold higher, and ORF2p production 20–40-fold higher, than by non-optimised human *L1* [28]. This would suggest an overall ratio of 8,000:1 to 160,000:1 for ORF1p:ORF2p, but that estimate may be unrepresentative since it relies on the properties of the synthetic *ORFeus*-Hs construct. Taylor et al. also made a more direct measurement, by intensity-based absolute quantification mass spectrometry, to estimate a relative stoichiometry of ∼20–30 ORF1p per ORF2p actually bound to (non-synthetic) *L1* mRNA in the cytoplasm [28]. However, the 20:1 to 30:1 estimate does not directly capture how much of each protein is normally actually produced, only what is bound to the mRNA. All told, the transcription and translation cost per functional *L1* ribonucleoprotein could be as low as $c = 9 \times 10^{-9}$ (an order of magnitude lower than for *Ty1*) or as high as 2.4×10^{-5} (three orders of magnitude higher than for *Ty1*). We stick with the better constrained estimates based on *Ty1*, but note that even the difference of a few orders of magnitude does not change our results qualitatively where transcription and translation costs are involved (Fig. 3, main text; Figs. S8, S9).

3.6 Protein aggregation

A further possibility is that overexpression of a given protein may trigger fitness consequences directly attributable to the aggregation of gratuitous protein. For example, Dong et al. [25] found that, in *E. coli*, overexpressing specific proteins to the extent that they constituted roughly 30% of overall protein production caused cell death associated with ribosomal degradation.

The data from Dong et al. [25] fit an equation for cell fitness (growth rate) of

$$g = 1 - x - Mx^2\,, \tag{68}$$

where $M = 7.1865$ and x is the fraction of overall protein production consumed by the overexpressed gene (Fig. S7B, C). We set the coefficient on x in the above function to 1 on

the supposition that this term represents the energetic cost of synthesizing a fraction x of the cell's proteins, while the term $-Mx^2$ represents the cost of aggregation and misfolding, which is proportional to the rate x^2 at which two proteins of the same type will interact in a cell. We then ignore the term x representing the energetic cost of synthesis, since we consider this cost in the "transcription and translation" fitness model above, and use the fitness function

$$w = 1 - Mx^2 \,. \tag{69}$$

In order to determine the fraction x of overall protein production for a given gene, we first need to know the total amount of protein produced in a typical cell. Lynch and Marinov [23] give the formula

$$N_{\mathrm{tot,p}} = (1.6 \times 10^6)V^{0.93} \tag{70}$$

for the total number of proteins in a given cell with volume V in $\mu\mathrm{m}^3$. For a cell with size 2,500 $\mu\mathrm{m}^3$, this gives $N_{\mathrm{tot,p}} \approx 2.3 \times 10^9$. If we assume that a typical eukaryotic protein comprises 400 amino acids [31], this gives a total amino acid production of 9.25×10^{11} amino acids per cell. Assuming, as above, 128,000 amino acids per fully-assembled transposon particle, this gives

$$x = 1.4 \times 10^{-7} k\bar{u}n \,, \tag{71}$$

where k is (as above) the number of transposon particles produced per successful duplication and $\bar{u}n$ is the total transposon activity.

For an alternative estimate, Tomala and Korona [32] report that overexpressing proteins to an average level of 1% of the total protein expression in yeast yields an average fitness cost of 2.3% for well-structured, cytosolic proteins (disordered or trans-membrane proteins carry an additional cost when overexpressed). This suggests an initially larger cost to aggregation and misfolding than the above formula would predict. However, we use the function derived from the data of Dong et al. [25] as it gives a fitness curve for multiple values of x rather than the fitness effect when $x = 1\%$ only.

4 Additional figures

This section contains additional results figures referred to in the main text.

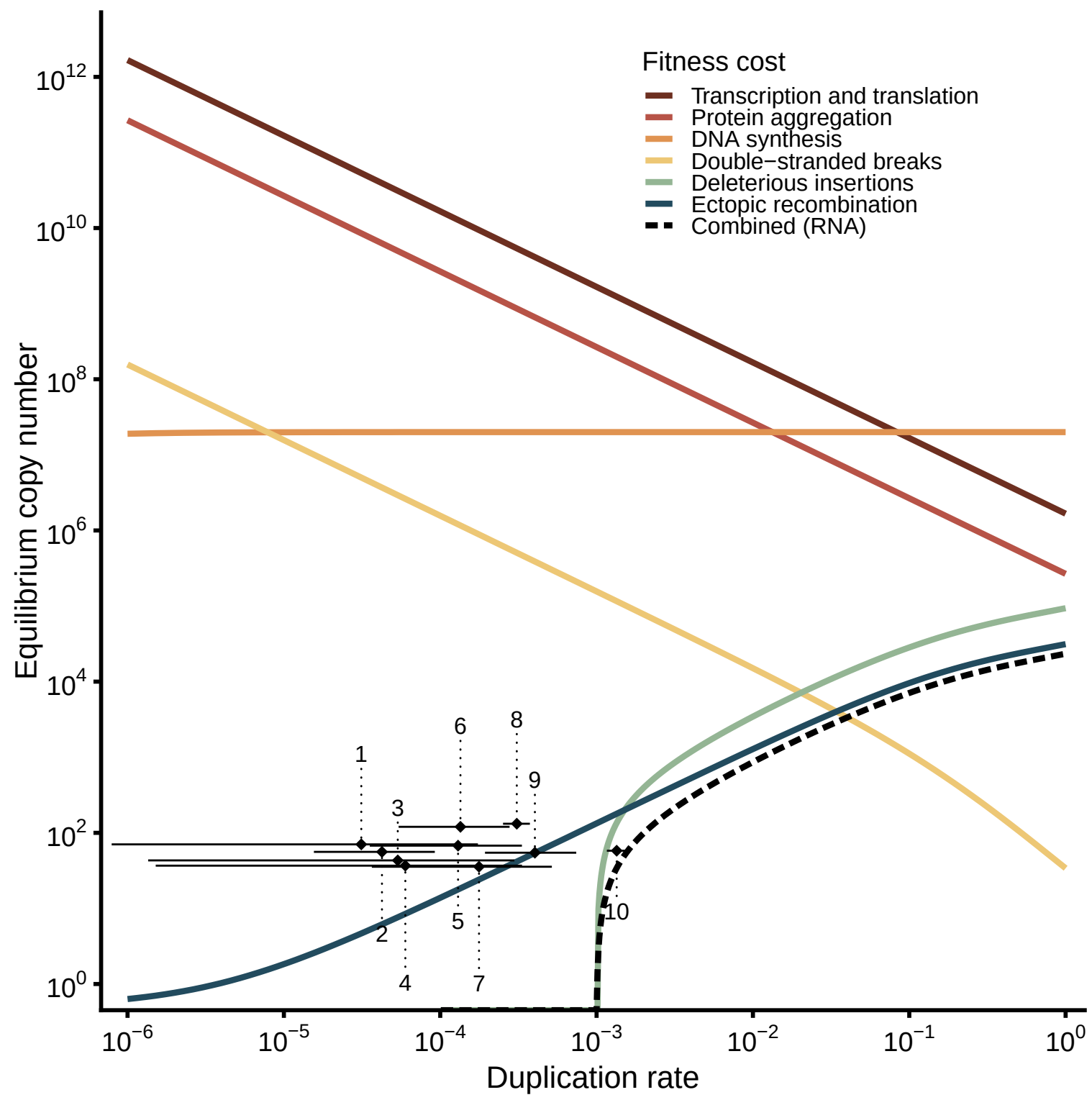

Figure S8: Strength of each identified fitness cost mechanism relative to empirical estimates for *Drosophila*. There are substantial differences in the relative strength of each cost mechanism, which we illustrate by plotting the expected equilibrium transposon number across a range of duplication rates for each cost. Numbered points with error bars show the estimated duplication rate and number of transposon copies found in the *Drosophila* genome from two studies: (1) jockey [33]; (2) *Doc* [34]; (3) *2244* [33]; (4) *opus* [33]; (5) *297* [33]; (6) *roo* [33]; (7) *mdg-1* [33]; (8) *roo* [34]; (9) *copia* [33]; (10) *copia* [34].

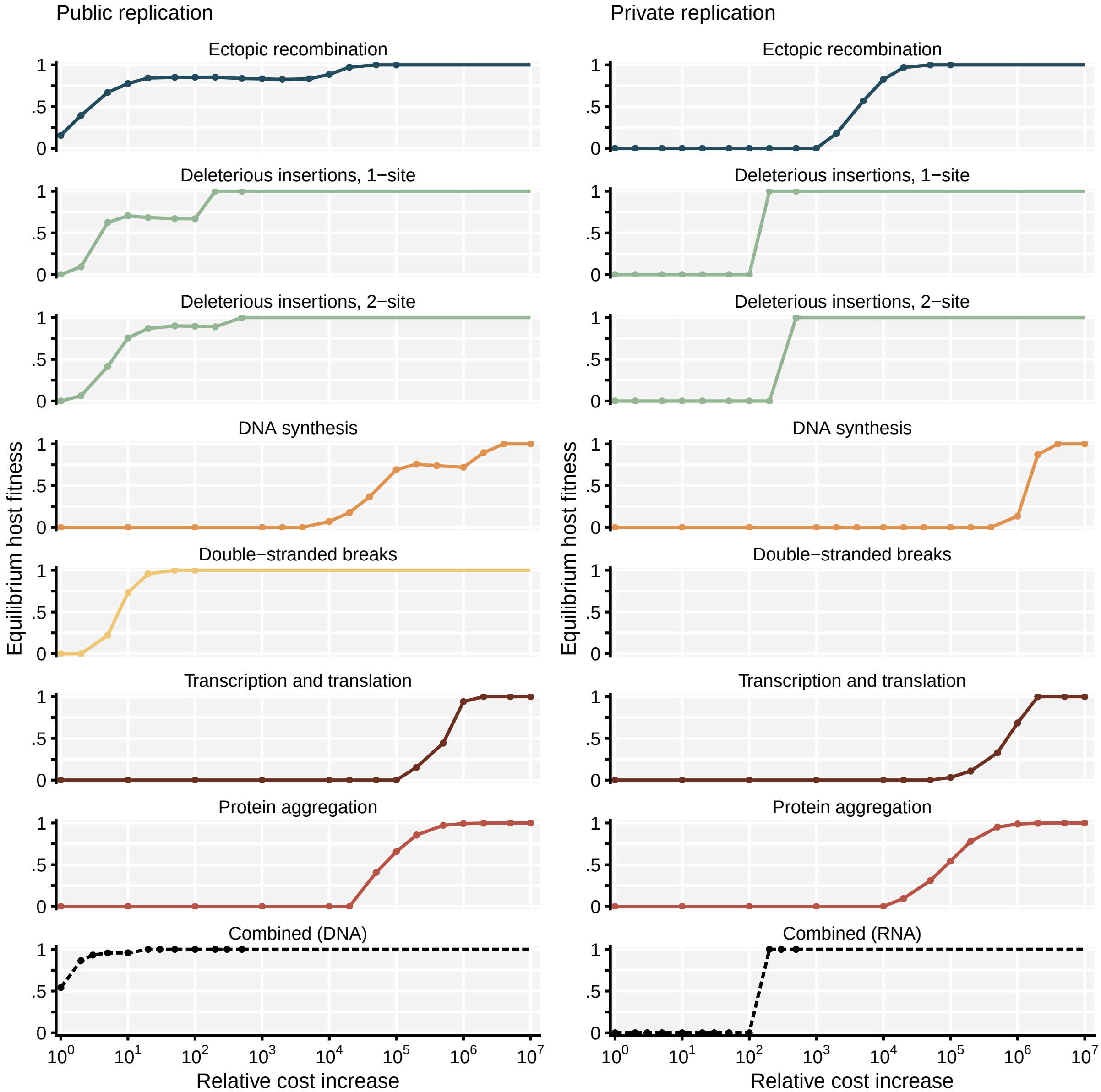

Figure S9: Comparison of equilibrium host fitness for each fitness cost under public replication versus private replication. Under public replication, costs are much more easily capable of keeping transposons in check than under private replication. In the former, there is a wide cost range for which the host population survives, while for the latter, there is only a relatively narrow region for each cost in which selection is strong enough to prevent extinction, but weak enough not to eliminate transposons entirely. Each panel shows the mean fitness across three runs of the stochastic model for each point; results were consistent across runs. We also show here an example of the deleterious insertions model where each potential transposon insertion site in the genome is either weakly selected (10 times weaker than in 1-site model) or strongly selected (same strength as in 1-site model). These two alternative modelling assumptions yield similar results. The “double-stranded breaks” model is not included for private replication as this cost does not apply to RNA transposons.

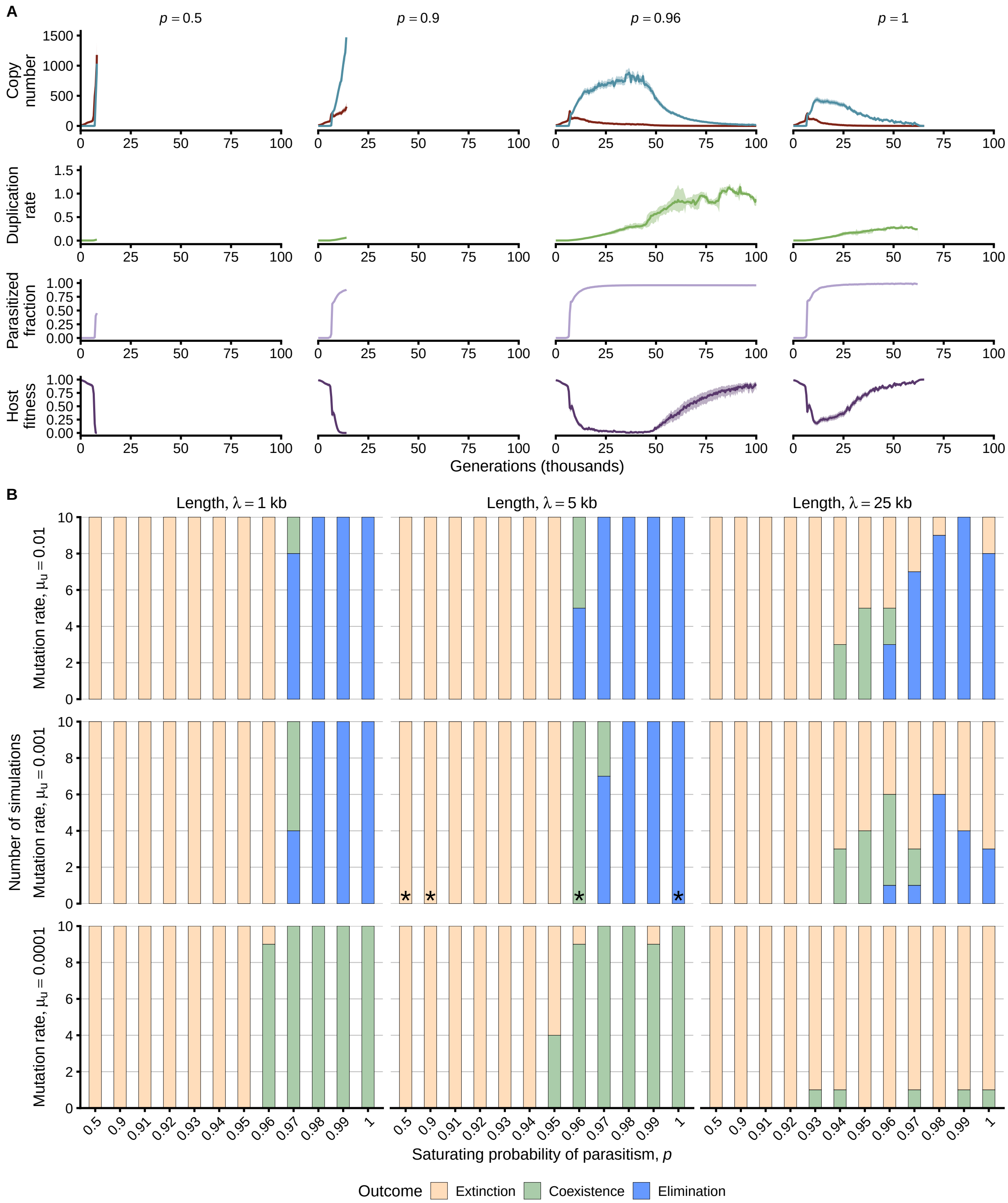

Figure S10: Sensitivity analysis for parasitic elements. (A) Example runs of the model with parasitism. Here, the probability of parasitic replication at saturation, p, is either 0.5, 0.9, 0.96, or 1 (columns); the length of the transposon $\lambda = 5$ kb; and the mutation rate for private and parasitic elements is $\mu_u = 0.001$ per element per generation. (B) Summary of 10 runs each across all parameter combinations of: parasitic replication p of 0.5, 0.9, 0.91, ..., 0.99, or 1.0; transposon length λ of 1, 5, or 25 kb; and mutation rate μ_u of 0.0001, 0.001, or 0.01. Asterisks represent the simulations shown in panel A. Recovery of the host population generally occurs for values of $p > 0.95$.

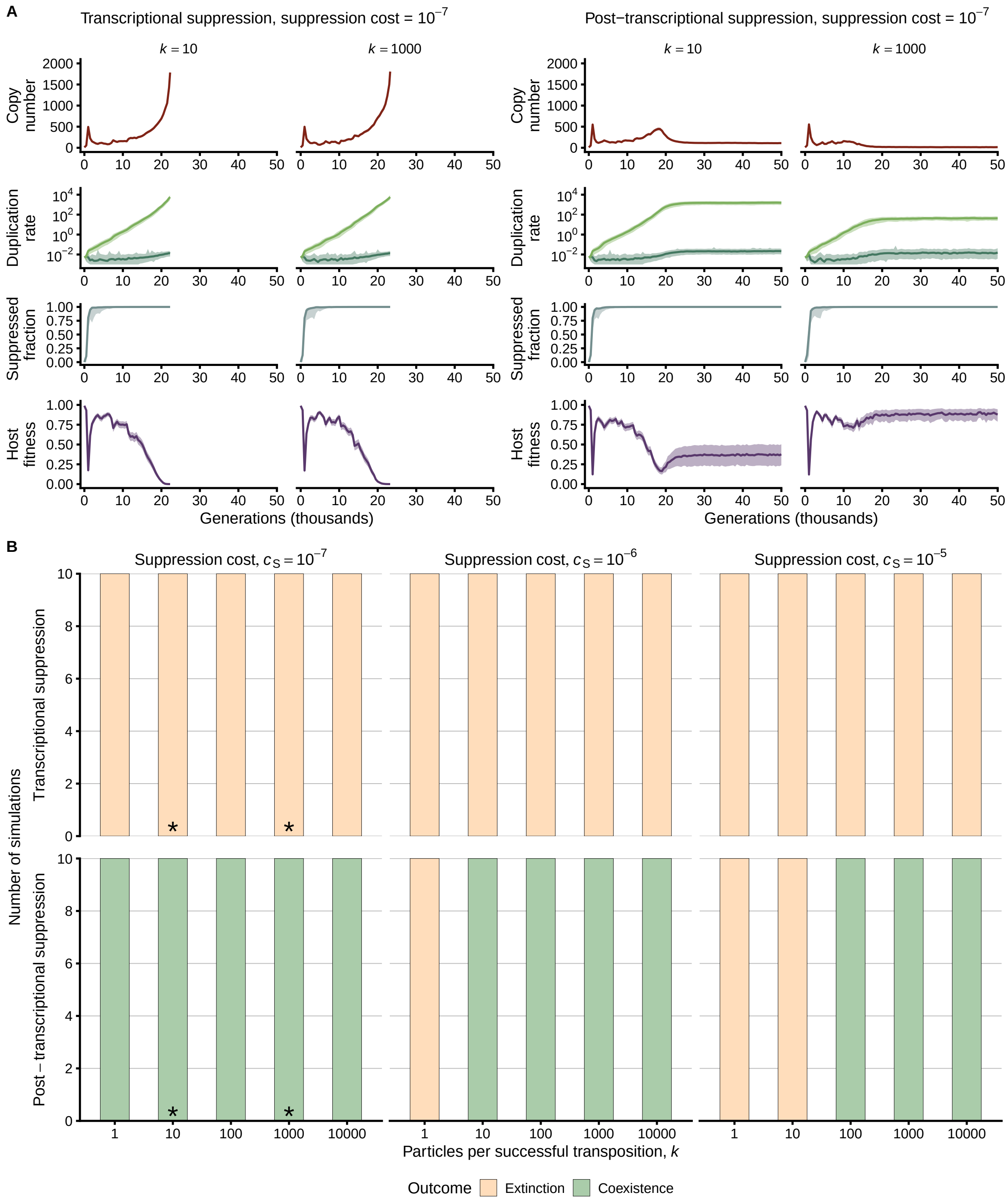

Figure S11: Sensitivity analysis for host transcriptional versus post-transcriptional suppression under private replication, for different values of the cost of suppression c_S and different values of k, the number of particles per successful transposition in the absence of suppression. (A) Example runs of the model for transcriptional versus post-transcriptional suppression, with $k = 10$ or 1000 and with $c_S = 10^{-7}$. (B) Summary of 10 runs each for transcriptional (top row) versus post-transcriptional suppression (bottom row); for suppression cost $c_S = 10^{-7}, 10^{-6}$, or 10^{-5}; and for k either 1, 10, 100, 1000, or 10,000. Asterisks represent the simulations shown in panel A. Under transcriptional suppression, all 150 runs resulted in host extinction (top row of panel B). Under post-transcriptional suppression, 120 out of 150 runs resulted in recovery of the host population, which requires a smaller value for c_S or a larger value for k.

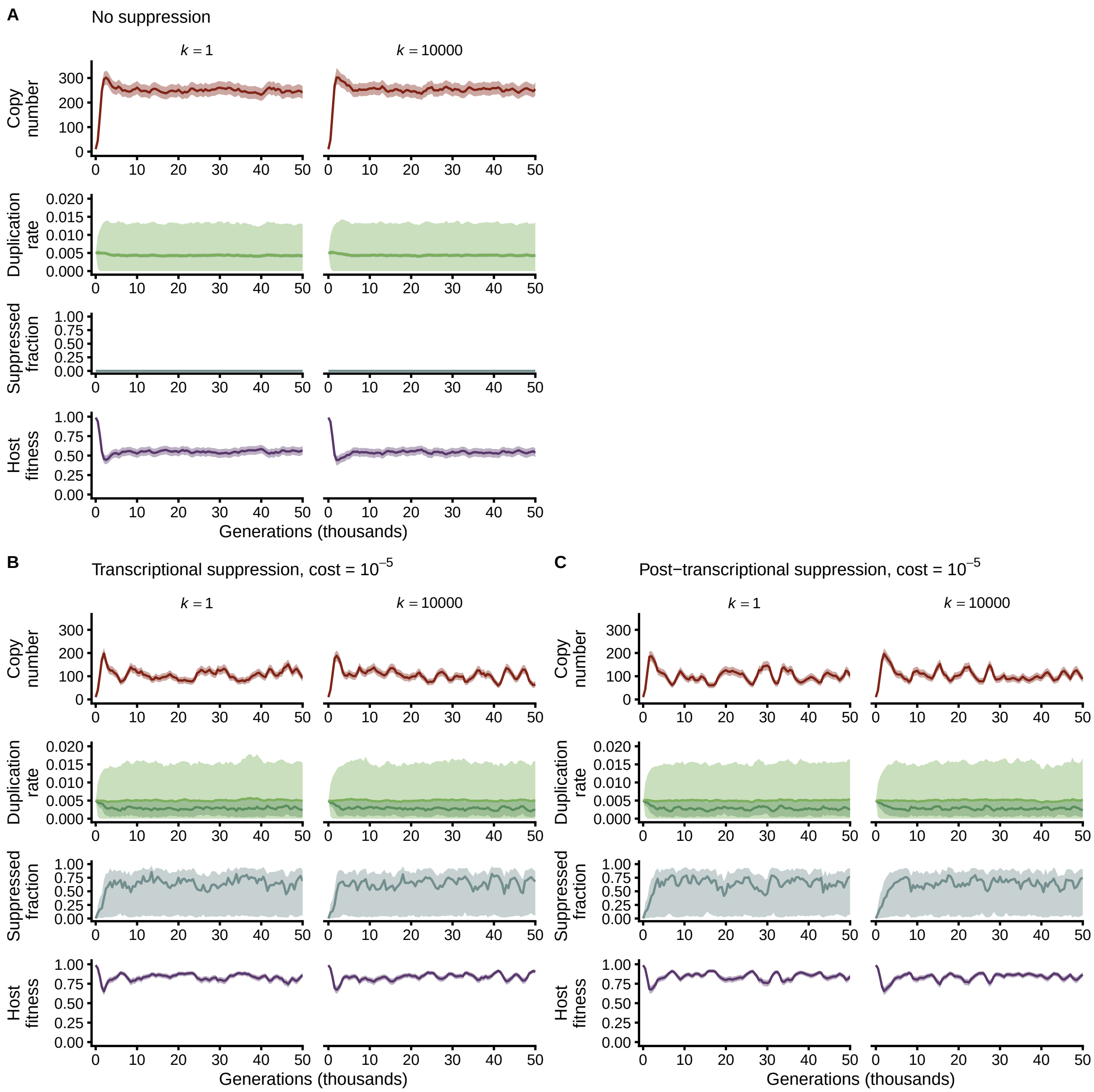

Figure S12: Host suppression under public replication, example runs under (A) no suppression, (B) transcriptional suppression, and (C) post-transcriptional suppression. Headings above each column show values of k, the number of particles per successful transposition in the absence of suppression. Host suppression reduces the effective duplication rate of publicly-replicating transposons, which reduces transposon copy number, partially recovering host fitness, and selects for transposons to moderately increase their activity (i.e. duplication rate in the absence of suppression) in response. Mean transposon activity in panels B and C is approximately 15% higher than in panel A. In the duplication rate plots, the lighter line shows transposon activity, while the darker line shows duplication rate (accounting for suppression). Similar results were found for $k = 10, 100, 1000$; each of the 15 parameter combinations was run 3 times with similar outcomes.

References

[1] B. Charlesworth and D. Charlesworth. “The population dynamics of transposable elements”. In: *Genet Res* 42 (1983), pp. 1–27.

[2] G.R. Price. “Selection and covariance”. In: *Nature* 227 (1970), pp. 520–521.

[3] S. Frank. “Natural selection. IV. The Price equation”. In: *J Evol Biol* 25 (2012), pp. 1002–1019.

[4] A. Grafen. “A theory of Fisher’s reproductive value”. In: *J Math Biol* 53 (2006), pp. 15–60.

[5] C. H. Langley et al. “On the role of unequal exchange in the containment of transposable element copy number”. In: *Genet Res* 52 (1988), pp. 223–235.

[6] Y. C. Lee and C. H. Langley. “Transposable elements in natural populations of *Drosophila melanogaster*”. In: *Philos Trans R Soc Lond B Biol Sci* 365 (2010), pp. 1219–1228.

[7] B. Charlesworth and D. Charlesworth. *Elements of Evolutionary Genetics*. Englewood, CO: Roberts & Company, 2010.

[8] P. S. Lee et al. “A fine-structure map of spontaneous mitotic crossovers in the yeast *Saccharomyces cerevisiae*”. In: *PLoS Genet* 5 (2009), e1000410.

[9] V. Merel et al. “Transposable elements in *Drosophila*”. In: *Mob DNA* 11 (2020), p. 23.

[10] A. S. H. Goldman and M. Lichten. “The efficiency of meiotic recombination between dispersed sequences in *Saccharomyces cerevisiae* depends upon their chromosomal location”. In: *Genetics* 144 (1996), pp. 43–55.

[11] N. D. Singh, P. F. Arndt, and D. A. Petrov. “Genomic heterogeneity of background substitutional patterns in *Drosophila melanogaster*”. In: *Genetics* 169 (2005), pp. 709–722.

[12] A. S. Fiston-Lavier et al. “*Drosophila melanogaster* recombination rate calculator”. In: *Gene* 463 (2010), pp. 18–20.

[13] G. A. T. McVean et al. “The fine-scale structure of recombination rate variation in the human genome”. In: *Science* 304 (2004), pp. 581–584.

[14] B. L. Dumont and B. A. Payseur. “Evolution of the genomic rate of recombination in mammals”. In: *Evolution* 62 (2008), pp. 276–294.

[15] J. P. Jaramillo-Correa, M. Verdú, and S. C. González-Martínez. “The contribution of recombination to heterozygosity differs among plant evolutionary lineages and life-forms”. In: *BMC Evol Biol* 10 (2010), p. 22.

[16] W. F. Eanes et al. “The fitness consequences of *P* element insertion in *Drosophila melanogaster*”. In: *Genet Res* 52 (1988), pp. 17–26.

[17] D. Houle and S. V. Nuzhdin. “Mutation accumulation and the effect of *copia* insertions in *Drosophila melanogaster*”. In: *Genet Res* 83 (2004), pp. 7–18.

[18] T. F. Mackay, R. F. Lyman, and M. S. Jackson. “Effects of *P* element insertions on quantitative traits in *Drosophila melanogaster*”. In: *Genetics* 130 (1992), pp. 315–332.

[19] S. F. Elena and R. E. Lenski. "Test of synergistic interactions among deleterious mutations in bacteria". In: *Nature* 390 (1997), pp. 395–398.

[20] R. P. Alexander et al. "Annotating non-coding regions of the genome". In: *Nat Rev Genet* 11 (2010), pp. 559–571.

[21] B. Chen et al. "Mapping of *Drosophila* mutations using site-specific male recombination". In: *Genetics* 149 (1998), pp. 157–163.

[22] A. García-Dorado and A. Caballero. "On the average coefficient of dominance of deleterious spontaneous mutations". In: *Genetics* 155 (2000), pp. 1991–2001.

[23] M. Lynch and G. K. Marinov. "The bioenergetic costs of a gene". In: *Proc Natl Acad Sci U S A* 112 (2015), pp. 15690–15695.

[24] K. Rothkamm et al. "Pathways of DNA double-strand break repair during the mammalian cell cycle". In: *Mol Cell Biol* 23 (2003), pp. 5706–5715.

[25] H. Dong, L. Nilsson, and C. G. Kurland. "Gratuitous overexpression of genes in *Escherichia coli* leads to growth inhibition and ribosome destruction". In: *J Bacteriol* 177 (1995), pp. 1497–1504.

[26] N. R. Burns et al. "Symmetry, flexibility and permeability in the structure of yeast retrotransposon virus-like particles". In: *EMBO J* 11 (1992), pp. 1155–1164.

[27] M. A. Checkley et al. "*P*-body components are required for *Ty1* retrotransposition during assembly of retrotransposition-competent virus-like particles". In: *Mol Cell Biol* 30 (2010), pp. 382–398.

[28] M. S. Taylor et al. "Affinity proteomics reveals human host factors implicated in discrete stages of *LINE*-1 retrotransposition". In: *Cell* 155 (2013), pp. 1034–1048.

[29] R. S. Alisch et al. "Unconventional translation of mammalian *LINE*-1 retrotransposons". In: *Genes Dev* 20 (2006), pp. 210–224.

[30] W. An et al. "Characterization of a synthetic human *LINE*-1 retrotransposon *ORFeus*-Hs". In: *Mob DNA* 2 (2011), p. 2.

[31] R. Milo. "What is the total number of protein molecules per cell volume? A call to rethink some published values". In: *Bioessays* 35 (2013), pp. 1050–5.

[32] K. Tomala and R. Korona. "Evaluating the fitness cost of protein expression in *Saccharomyces cerevisiae*". In: *Genome Biol Evol* 5 (2013), pp. 2051–2060.

[33] X. Maside, S. Assimacopoulos, and B. Charlesworth. "Rates of movement of transposable elements on the second chromosome of *Drosophila melanogaster*". In: *Genet Res* 75 (2000), pp. 275–284.

[34] S. V. Nuzhdin and T. F. Mackay. "Direct determination of retrotransposon transposition rates in *Drosophila melanogaster*". In: *Genet Res* 63 (1994), pp. 139–144.
