# Supplementary material for: Cooperation, competition and enforcement in transposon evolution
Source: Nat Commun. 2026 Aug 12;17:9689. doi: 10.1038/s41467-026-75312-1 (PMC13558583; doi:10.1038/s41467-026-75312-1)
Supplement: Supplementary file 2 [file 41467_2026_75312_MOESM2_ESM.pdf]

nature portfolio

Peer Review File

The tragedy of the transposons: cooperation, competition and enforcement

Corresponding Author: Dr Nicholas Davies

This file contains all reviewer reports in order by version, followed by all author rebuttals in order by version.

Version 0:

Reviewer comments:

Reviewer #1

(Remarks to the Author)
Review of "The tragedy of the transposons: cooperation, competition and enforcement" by Dr Davies and colleagues, NCOMMS-24-77230.

This manuscript explores a range of theoretical models dedicated to the evolution of transposable elements. More specifically, the authors aim at understanding the mechanisms that drive the functional evolution of TE sequences, and in particular what prevents competition among TEs to increase the transposition rate to unsustainable levels. Model exploration shows that the expectation varies substantially depending on how TEs share the replication machinery with related copies. In a "public replication" model, transposition machinery is a common resource, and the reward to increase this resource is limited. In contrast, for "private replication" TEs, the transposition machinery can only be used by the TE copy that produced it, promoting a never ending increase of the transposition rate, and eventually the extinction of the host species, unless transposition becomes regulated by the host.

Overall, the manuscript is interesting and thought provoking. Although similar models exist, dedicated to e.g. the evolution of pairs of autonomous/non-autonomous TEs, I think the authors' approach focusing on the interaction between intra-genomic selection (efficiency of selfishness) and cis-preference is original and informative about the evolutionary mechanisms underlying the dynamics of TE sequences.

On the down side, I found the ms quite difficult to review, because of the structure of the manuscript and of the mismatches between the main text and the long (9 pages) section named "Methods", as well as the 40 pages (sic) of supplementary methods. My interpretation is that the ms might have been forced into a format that harms its scientific content (pedagogical figure panels, simplistic equations, methods as supplement). For instance, I am not sure to understand how the figure panels were generated, nor how the "deterministic model" and the "invasion analysis" from the methods were exploited in the main text. As a consequence, the "results" section seems to focus on verbal arguments and mixes elements of context and discussion, using a small set of simulations to illustrate qualitative expectations, as if the manuscript was a "perspective/review" paper based on the supplementary methods, in which the rigorous scientific content is exposed.

More detailed comments follow.

** Major comments

1. The long "methods" section probably needs to be clarified and reorganized; its connection with the main text also needs to be made more obvious.

1.a. For instance, the "methods" start with the description of a "deterministic model", but I could not figure out where this model was used and what was its purpose. Note that I was not convinced by the terminology, since this "deterministic" model is a probabilistic model that explicilty handles the distribution of the random variables. In any case, the paper seems to be based on simulations, so the rationale for this deterministic version seems obscure.

1.b. The "host fitness function" section of the methods comes very late and it would make sense to have it before "Simulation details". In any case, these 6 selection models are extremely interesting, as they offer fitness functions of different shapes.

Given that the shape of the fitness function is essential for understanding TE dynamics, I was frustrated to see how marginal they were in the results (for instance, what is the effect of the fitness function on public transposition, etc).

1.c. I am uncertain about what the "Invasion analysis" section brings to the manuscript. It is developed in the "Methods" section while it clearly presents new results, and I am not sure to understand how to relate these results to the questions addressed in the main text. Is the rationale for this 5-page mathematical development solely to provide expressions for B and C (equations 19 and 20) that are even not reported in the main text because too "complex"? (line 180).

2. Methodological details are missing to interpret the simulations. In particular, figure panels report a single line + shaded area around, but there is no explanation not consistency across figures. Do the panels report single simulation runs + variation in the population, or multiple simulation runs + variation among replicates? Scenarios in panels 1G and 1I are clearly manipulated for instance, which is very different from other panels were populations evolved freely. I found extended data 1 a bit worrying, because it shows that single simulation runs can be strongly affected by stochasticity (results are very unconsistent for p=0.94, p=0.95, p=0.96 and p=0.97, for instance). This excludes the strategy of considering single simulation runs, at least for the "parasitism" case; the variable of interest is not the copy number, but rather the probability of TE elimination.

I also doubt that the raw time series from the simulations is always the best representation of the simulation results, especially because it makes the exploration of the parameter space very cumbersome (as in extended figures). For instance, in Ext fig 1, the variable to be reported should be the probability of extinction as a function of p (and possibily other parameters), for fig 3 it could be the time before extinction, etc. This would allow a substantial simplification of the panels, as well as the possibility to compare different factors (e.g. private vs public transposition in fig 3).

3. The modeling choice to consider private/public transposition as discrete binary strategies should be discussed more, and possibly turned into a quantitative parameter similar to parameter p for the parasitic replication. When considering for instance Zhang et al 2020, cited in the manuscript, the Ka/Ks ratio used as a measurement for cis-preference seems to span uniformly between 0.2 (close to functional proteins, and probably close to the "100% private case) and 1.0 (neutrality, "public"). This suggests that most TE families are somehow mid-way between public and private, while very few TE families behave as in the simulated model. In particular, the description of the "parasitic" model was not trivial in the "private" context, because it requires a breakage of the "private transposition" principle, but only towards non-autonomous copies. Considering that imperfect privacy is a prerequisite for the emergence of non-autononous TEs thus seems more natural, and would open new interesting issues, such as the level of privacy from which the ever-increasing transposition rate could start.

** Minor comments

4. In the abstract and later in the manuscript, the authors attribute the never ending increase of the transposition rate as a case of tragedy of the commons. Although competitive tragedy might somehow be related to tragedies of the common, I am not completely sure it is the same phenomenon. Paradoxically, I would tend to think that what happens to public transposition copies is closer to the tragedy of the commons, because public TEs have no incentive to participate to the pool of transposition machinery, which degrades their ability to code for a functional protein.

Later in the manuscript, the authors compare the threshold from which selection promotes higher duplication rates to Hamilton's rule, related to social behavior. This seems conceptually close to the attempts at comparing TE interactions to ecological interactions (Venner, S., Feschotte, C., & Biemont, C. (2009). Dynamics of transposable elements: towards a community ecology of the genome. Trends in Genetics, 25(7), 317-323. and other related papers). As far as I can tell, these comparisons are only vague analogies, which do not really catch the complexity of TE interactions. Perhaps it would be worth discussing the differences between these two similar analogies, and what the social behavior analogy brings to the ecological analogy?

5. Line 186 and other places in the manuscript: I was confused about the authors' use of "natural selection" in this context. If my undertsanding is correct, the authors wish to compute the equivalent of inclusive fitness for a TE copy, accounting for both Darwinian selection at the individual level (decrease in the transmission rate across generations) and intragenomic selection (increase in copy number due to replicative transposition). This probably makes sense, but I am not sure "natural selection" properly catches the idea. I am not sure a consensual definition of "natural selection" exists, but I would typically exclude non-phenotypic selection (e.g. selfish DNA) from natural selection.

6. Around line 324 and in other related sections, it should be noted that the "evolutionary race" is only possible when considering that transposition rates could increase forever. If the transposition rate was physically of biochemically bounded, the race could stop (although population extinction may still happen sooner or later because of the genetic load).

7. Line 421, the equation is not correct in the manuscript, because i and j should run from 0 to Inf (and not to n). Note that the equation is correct in the supplementary methods. Moreover, U1 and U2 are not defined here.

8. Line 457, is the mean number of chiasma per chromosome or for the whole genome?

(Remarks on code availability)

Reviewer #2

(Remarks to the Author)
The paper by Davies, Agren and Foster describes a theoretical work about the evolution of the transposition rate of transposons given a public or private mode of replication. It is a very interesting work pointing out that we expect different evolutionary outcomes for TE families that may replicate publicly and privately. Also the parallels to kin-selection (Hamiltons rule) and the tragedy of the commons are fascinating. Additionally the level of scholarly is impressive, where they for example point out the six different negative effects that TEs may have (I was solely aware of three). Finally I have to say that the manuscript is amazingly well written. Concepts are nicely explained, with illustrative figures and the style makes it easy to follow.
However it is a theoretical study and as such has the typical problem of any theoretical work, i.e. it is not clear how well the models and assumptions reflect the actual biology of the organisms and the TEs, and thus how relevant the work is to the field. Nevertheless I consider it an important contribution.

I have some major/minor comments

major
- The six different negative effects of TEs are interesting. However I think modelling the effects of all of them is excessive, and perhaps conveys the wrong impression that we understand these processes well enough so that we are able to accurately model them. I would argue that our knowledge about the biology of TEs is currently far too limited to attempt this and therefore a simple (or a few simple) and more general models of negative selection would have been entirely sufficient. For example we do not even understand the simplest model, i.e. the direct effect of the TE insertion. They assumed that all insertions have equal fitness effects, but this is likely not true. Some could be neutral, others slightly deleterious, a few deadly and even fewer positively selected. So there is an unknown distribution of fitness effects of TE insertions that will likely differ among the families. Moreover, they assumed that the fitness impact of TEs increases exponentially with TE copy numbers (n^2 in Fig2 caption). This is of course in agreement with the model of Charlesworth1984 which requires an exponential increase in fitness cost to obtain a stable TE equilibrium (without host defence). But it is entirely unclear if this assumption holds. Next, it is not clear what are the fitness effects of homozygous vs heterozygous insertions and how they epistatically interact. As another example, with ectopic recombination we have again so many unknowns. does the rate of ectopic recombination depend on the recombination rate, which varies through the genome (which is not considered in the manuscript) , what is the distribution of fitness effects of ectopic recombined chromosomes; does the rate of ectopic recombination depend on the length of the TEs (I do not think this was considered). Do we have more ectopic recombination for heterozygous than for homozygous insertions? How is the model affected if the rate of ectopic recombination is very heterogenous in the genome? And similarly long lists of unknowns can be made for all 6 negative effects of TEs. Therefore I think it is a bit excessive trying to model these many negative effects, and it conveys a wrong sense of accuracy.
Just to be clear I am not suggesting to make their model more complex but rather the opposite, to perhaps limit it to a very few ones that capture the main message.
- In a similar direction I think the conclusion that TGS cannot prevent extinction and PTGS is necessary to stop TEs, is a pure artifact of their assumptions. I do not see how an arms race is a necessary outcome of TGS. For example, in Drosophila, a TE insertion in a piRNA cluster leads to piRNAs that direct TGS and PTGS. Any mutation in the TE leads to mutated piRNAs, that attack the mutated TE. So I do not see how this can possibly lead to an armsrace between the TE and the host. Additionally I do not see how this adaptation to the mutated TE increases the cost of TGS (the same amount of piRNAs, the same amount of silenced loci etc). Also as far as I can tell they assume a fitness cost for TGS but not for PTGS, so no surprise this PTGS which does not incur any fitness cost seems necessary to stop TEs. Therefore this conclusion is just an outcome of their model assumptions, which are to my knowledge not supported by biology. Furthermore its a bit unintuitive to me. In my mind, TGS must be way less costly to the host than PTGS, since TGS prevents the transcription of TEs, and thus saves resources. While in PTGS, the TEs are transcribed and then degraded, a very wasteful processes that spends a lot of energy (which the authors mention in the manuscript).
- the finding that a host defence can prevent extinction of host populations was already previously shown, even with constant transposition rates. I think this should be acknowledged https://academic.oup.com/mbe/article/36/7/1457/5435964

minor
- Just as a possibility, I leave this to the discretion of the authors: I would move the description on which TE families are public (DNA transposons) and which are private (non-LTR and perhaps LTR) to the introduction. Initially, when reading the manuscript I assumed they make a straw-man argument, distinguishing between public/private with little biological examples for each category. Only in the results it became clear to me that some TE families are clearly public while others are very likely private, so the distinction between public/private TE families is an important one, supported by many biological examples.
- one testable prediction of their model could be that private TEs should have evolved a higher transposition rate than public ones? Or is this solely expected when TEs are kept in check just by negative selection (as in Fig2). Do we also expect this under models with a host defence scenario? As far as I can tell the effect of the host defence was only modeled for private TEs (as extinctions only occured for those). Perhaps the authors can add to the discussion a paragraph about testable hypothesis arising from their work?
- eg line 546, I think the dashed square is a LaTeX error?
- initially I assumed that public refers to interactions between different two TE families. But nevertheless, I was wondering could this happen, i.e. that some proteins produced by one family are then used by another one? Would this have any influence on their model predictions? Perhaps this can be a bit discussed. Or is this perhaps just a ‘special’ scenario of the

non-autonomous insertion scenario which they modeled - where the other family is basically like the non-autonomous parasite?
- 271 and further: references missing about non-autonomous TEs parasitizing the autonomous ones
- 310 'piRNAs silence TEs through DNA methylation'. This is not correct, e.g. Drosophila has no methylation, yet TGS silences TEs (by epigenetic histone modifications).

(Remarks on code availability)

Version 1:

Reviewer comments:

Reviewer #1

(Remarks to the Author)
Review of "The tragedy of the transposons: cooperation, competition and enforcement" by Dr Davies and colleagues, NCOMMS-24-77230A.

This is a revised version of ms NCOMMS-24-77230 I reviewed last year. I found the previous version interesting and original, but I had a series on concerns, which can be summarized as (i) the organisation of the manuscript and the relationships between the main text and the supplementary material was suboptimal, (ii) important methodological details were missing, and (iii) modeling public/private transposition as binary strategies may not match the subtle evolutionary complexity of the question. Reviewer #2 had different concerns, most being related to the approximations of the selection model.

The authors made a tremendous effort at trying to address both reviewers' remarks, both in their detailed response letter and in the revision.

Some parts of the manuscript have been deeply re-written, and the authors did their best to improve the independence between the main text and the supplement. This partly fixes my first concern, and I assume this is acceptable given that the problem was mostly due to the mismatch between the scientific content and the journal's constraints and editorial line.

My concern #2 has been completely addressed. The manuscript now clearly distinguishes the stochastic model and the deterministic model, and reports which panel was generated with which model.

My concern #3 was more challenging to handle, since it would have involved an additional parameter in the model, and thus changes in the simulation software etc. The authors decided to stay on the binary option for the model, and to add a discussion paragraph to state that some TE families could lay in-between. I assume this is acceptable. Note that I slighly disagree with the authors' argument about non-binary model not being "in the scope" of this work; I really think that this would have been totally in the scope, but the authors prefered to explore other model dimensions (e.g. different fitness functions) instead.

Otherwise, there were a couple of minor points for which the authors disagree with my suggestions, which is perfectly fine. Overall, I feel that I do not have any further suggestion, and no more reason to oppose publication.

(Remarks on code availability)

Reviewer #2

(Remarks to the Author)
I think the authors did a great job addressing my comments.
Although I was hoping for a different response with some comments (eg to make the model simpler and not even more complex) I see no reason to further delay publication of this important contribution.
I think this work is suitable for publication.

(Remarks on code availability)

Open Access This Peer Review File is licensed under a Creative Commons Attribution 4.0 International License, which permits use, sharing, adaptation, distribution and reproduction in any medium or format, as long as you give appropriate credit to the original author(s) and the source, provide a link to the Creative Commons license, and indicate if changes were made.
In cases where reviewers are anonymous, credit should be given to 'Anonymous Referee' and the source.
The images or other third party material in this Peer Review File are included in the article's Creative Commons license, unless indicated otherwise in a credit line to the material. If material is not included in the article's Creative Commons license and your intended use is not permitted by statutory regulation or exceeds the permitted use, you will need to obtain permission directly from the copyright holder.
To view a copy of this license, visit https://creativecommons.org/licenses/by/4.0/

REVIEWER COMMENTS

Reviewer #1 (Remarks to the Author):

Review of "The tragedy of the transposons: cooperation, competition and enforcement" by Dr Davies and colleagues, NCOMMS-24-77230.

This manuscript explores a range of theoretical models dedicated to the evolution of transposable elements. More specifically, the authors aim at understanding the mechanisms that drive the functional evolution of TE sequences, and in particular what prevents competition among TEs to increase the transposition rate to unsustainable levels. Model exploration shows that the expectation varies substantially depending on how TEs share the replication machinery with related copies. In a "public replication" model, transposition machinery is a common resource, and the reward to increase this resource is limited. In contrast, for "private replication" TEs, the transposition machinery can only be used by the TE copy that produced it, promoting a never ending increase of the transposition rate, and eventually the extinction of the host species, unless transposition becomes regulated by the host.

Overall, the manuscript is interesting and thought provoking. Although similar models exist, dedicated to e.g. the evolution of pairs of autonomous/non-autonomous TEs, I think the authors' approach focusing on the interaction between intra-genomic selection (efficiency of selfishness) and cis-preference is original and informative about the evolutionary mechanisms underlying the dynamics of TE sequences.

We appreciate this positive assessment from the reviewer.

On the down side, I found the ms quite difficult to review, because of the structure of the manuscript and of the mismatches between the main text and the long (9 pages) section named "Methods", as well as the 40 pages (sic) of supplementary methods. My interpretation is that the ms might have been forced into a format that harms its scientific content (pedagogical figure panels, simplistic equations, methods as supplement). For instance, I am not sure to understand how the figure panels were generated, nor how the "deterministic model" and the "invasion analysis" from the methods were exploited in the main text. As a consequence, the "results" section seems to focus on verbal arguments and mixes elements of context and discussion, using a small set of simulations to illustrate qualitative expectations, as if the manuscript was a "perspective/review" paper based on the supplementary methods, in which the rigorous scientific content is exposed.

We have now substantially clarified the links between the main text and the Methods section. This includes reorganising the methods section to better match the structure of the paper, more clearly specifying how the figure panels were generated, and stating where the deterministic model vs. stochastic model vs. invasion analysis were used throughout the main text. Please see the more detailed responses below for details.

More detailed comments follow.

** Major comments

1. The long "methods" section probably needs to be clarified and reorganized; its connection with the main text also needs to be made more obvious.

We have reorganised the Methods section to better match the presentation in the main text, and clarified its connections to the main text by explicitly referring the reader to the relevant sections of the Methods at the appropriate places.

Specifically, the methods section is now broken down into three numbered subsections:

1. Basic models

    1.1 Deterministic population-genetics model
    1.2 Stochastic individual-based model

2. Simulation details

    2.1 The tragedy of the transposons (relating to Fig. 1)
    2.2 Host fitness costs (relating to Fig. 3)
    2.3 Parasitic replication (relating to Fig. 4A-C)
    2.4 Host suppression (relating to Fig. 4D-F)

3. Invasion analysis (relating to Fig. 2)

    3.1 Overview
    3.2 Benefit of duplication event
    3.3 Cost of duplication event
    3.4 Derivation of condition

Section 1 relates to the basic model assumptions, and we explicitly refer to sections 1.1 and 1.2 when describing the deterministic model and the stochastic model at the beginning of the Results section ("Details of the two model implementations are given in the *Methods* 1.1–1.2").

Section 2 of the Methods relates to additional assumptions made for each set of simulations presented throughout the paper. This section now gives all the details for the simulations presented in the results section in the order they are presented in. We now clearly refer the reader to sections 2.1–2.4 of the Methods in the Results section (e.g. "[...] the result is a stable equilibrium in transposon abundance, as predicted by Charlesworth and Charlesworth[24] (Fig. 1E; ***Methods* 2.1**)", similar in other sections of the Results) and in the captions to Figures 1, 3, and 4 (e.g. "See *Methods* 2.1 for simulation details" in Fig. 1, similar in Figs. 3 & 4).

Section 3 of the Methods now contains the invasion analysis, and we refer the reader to this section in the Results section ("we conducted a mathematical invasion analysis of our deterministic model in which a rare mutant lineage emerges among an established

wild-type population of transposons (*Methods* 3.1–3.4)") as well as in the caption to Fig. 2 ("See *Methods* 3.1–3.4 for details").

1.a. For instance, the "methods" start with the description of a "deterministic model", but I could not figure out where this model was used and what was its purpose.

We see that it wasn't clear enough which model was being used where. We now specify where the deterministic model versus the stochastic model is being used throughout the text of the Results, and in all figure captions. To clarify the purpose of both models, we have also expanded on the explanation of the two model types in the first paragraph of the Results:

> "We use **two implementations of this model for simulations**. A deterministic, discrete population-genetics model is used for simulations involving up to two competing transposon types (i.e., wild-type and mutant), **and allows us to neglect genetic drift by assuming an infinite host population**. A stochastic individual-based model is used for simulations **in which we allow transposon mutation rates to evolve freely, and allows us to consider an arbitrary number of competing transposon types by simulating a finite host population.**"

Note that I was not convinced by the terminology, since this "deterministic" model is a probabilistic model that explicilty handles the distribution of the random variables.

We think this may be a misunderstanding by the reviewer, so we have clarified the text in this regard as quoted above. Our deterministic model calculates a single, nonrandom trajectory under the assumption of an infinite population. The mathematical equations for this model use probability mass functions, but not to capture the distribution of "all possible realisations" of a random trajectory, but to compute all the combinatorial assortments of transposon numbers among sexually reproducing hosts in an infinite population.

Conceptually, it is the same as a model that tracks genotype frequencies at a biallelic locus in an infinite population from one generation to the next. In that kind of model, the genotype frequencies would be notated e.g. f_{AA}, f_{Aa}, and f_{aa}. Our model is similar in concept, it just has to track more genotypes. For example, in the deterministic model with one transposon type, the genotype frequencies are f_0, f_1, f_2, ..., f_{n_max}. With a second transposon type, we have $f_{0,0}$, $f_{0,1}$, $f_{1,0}$, $f_{1,1}$, and so on. It is still correct to refer to this as a deterministic model. We have clarified the infinite-population assumption in the main text (quoted above).

In any case, the paper seems to be based on simulations, so the rationale for this deterministic version seems obscure.

Both the deterministic model and the stochastic model are used for simulations in the paper. To avoid confusion, we now specify where the deterministic model is being used and where the individual-based model is being used throughout the text of the Results, and in all figure captions. For example, we now have:

“Next, we introduce a mutant transposon into the ~~population~~ **deterministic model**”

“Therefore, we incorporated parasitism into our **individual-based** model”

Both model versions are needed for the paper. The stochastic individual-based model allows us to let the transposon duplication rate evolve freely, since each transposon in this model has its own specific duplication rate. However, this requires that we model a finite number of hosts, which means that the stochastic model exhibits genetic drift. By contrast, the deterministic model allows us to look at simulation results “cleanly” without genetic drift, by assuming an infinite population size; this model is also easier to analyse mathematically, which yields the invasion analysis.

We have clarified the reasons for using both model types in the paragraph quoted above. We also now clarify that the deterministic model furnishes the invasion analysis in the main text:

> “To explain these different outcomes, we conducted a mathematical invasion analysis **of our deterministic model** in which a rare mutant lineage emerges among an established wild-type population of transposons.”

1.b. The "host fitness function" section of the methods comes very late and it would make sense to have it before "Simulation details".

We have now moved this section earlier as suggested by the referee.

In any case, these 6 selection models are extremely interesting, as they offer fitness functions of different shapes. Given that the shape of the fitness function is essential for understanding TE dynamics, I was frustrated to see how marginal they were in the results (for instance, what is the effect of the fitness function on public transposition, etc).

In response to this point, we have expanded our discussion of the 6 fitness functions in the main text and added additional Extended Data figures (Extended Data Figs. 1 and 2). These explain differences among the 6 costs in terms of their relative strength (Extended Data Fig. 1), analyse the effect of the fitness function on public transposition (Extended Data Fig. 2), and give additional details on how host suppression interacts with activity costs to resolve the tragedy of the transposons.

Specifically, we:

(1) added text with additional details on the mathematical properties and usage of the fitness functions:

> For each function, the relative burden to the host of each additional transposon increases as more transposons accumulate (i.e., each function is log-concave with respect to transposon number). This was established by Charlesworth and Charlesworth[24] as a necessary condition for the transposon number to reach equilibrium, assuming a fixed duplication rate. We integrated these host fitness

functions into our individual-based model, allowing the transposon duplication rate to evolve freely. *[Results, p. 10]*

(2) discuss an additional figure (Extended Data Fig. 1) comparing the inherent strength of each fitness function, which has revealing implications for which costs are plausible:

> This is partly because many of these costs are inherently weak. Indeed, we find that four of the six costs proposed in the literature (costs iii-vi) are too weak to meaningfully inhibit transposon proliferation, at least when acting on their own (Extended Data Fig. 1). This has perhaps gone unnoticed because previous studies have only mentioned these as potential costs, without estimating the strength of selection that would be associated with them. The two remaining costs—ectopic recombination (cost i) and deleterious insertions (cost ii)—could potentially keep transposon number in check, if the duplication rate were fixed. *[Results, p. 10]*

(3) discuss an additional figure (Extended Data Fig. 2) showing the effect of the fitness function on public transposition, as requested by the reviewer:

> Compare this to publicly-replicating transposons, in which a realistic cost model for DNA transposons easily allows the survival of the host population (Extended Data Fig. 2). *[Results, p.11]*

(4) and link our findings on host post-transcriptional suppression to the distinction made earlier between copy-number costs and activity costs:

> Earlier, we argued that the fitness costs associated with transcription, translation, and protein aggregation (costs v and vi, Fig. 3b) were too weak to meaningfully restrict transposon proliferation when acting on their own (Extended Data Fig. 1). But in the context of a high level of post-transcriptional suppression, these transposon activity costs become substantially stronger, because the effect of post-transcriptional host suppression is to vastly increase the production rate of mRNA and protein required for transposition to be successful. *[Results, p. 13]*

1.c. I am uncertain about what the "Invasion analysis" section brings to the manuscript. It is developed in the "Methods" section while it clearly presents new results, and I am not sure to understand how to relate these results to the questions addressed in the main text. Is the rationale for this 5-page mathematical development solely to provide expressions for B and C (equations 19 and 20) that are even not reported in the main text because too "complex"? (line 180).

The full invasion analysis, which we cannot present in the main text, is found in the Supplementary Methods. This analysis is critically important for our paper and we believe that it represents a substantial theoretical advance. However, this comment from the reviewer shows that we were not presenting this material clearly enough. Originally, we presented simplified analytical results in the Results, an abridged version of the invasion analysis in the Methods, and the full invasion analysis in the Supplement.

We have removed the abridged version of the invasion analysis from the Methods. We still provide the simplified results of this analysis in the Results, but we now justify these by presenting an intuitive argument directly in the Results rather than by requiring the reader to progress through 5 pages of derivation in the Methods. In the Methods, we now explain where to find the full invasion analysis and explain why the intuitive argument presented in the Results is only an approximation. We hope that the material presented in the main text is now easier to grasp, while the reduced material in the "main text" Methods section makes it easier to understand why the results in the main text are only an approximation and why the full analysis is justified.

The Results in the main text now state:

Evolutionary logic

Why does the mode of transposon replication (public versus private) lead to such fundamentally divergent outcomes? To answer this question, we conducted an invasion analysis of our deterministic model. This method allows us to derive a mathematical condition to explain when an increase to the transposon duplication rate is favoured by natural selection (see *Methods* 2.2). A simplified version of the argument is provided here, while the full (and more rigorous) analysis is presented in *Supplementary Methods* 2.

Consider a population of hosts in which the average number of transposons per host genome is $\bar{n}$, and all transposons duplicate at rate u (for simplicity, we assume the loss probability v is 0). A transposon mutates to have a marginally higher duplication rate. Focus on an instance where, by virtue of its higher duplication rate, the mutant transposon (the "parent") generates one new transposon (the "offspring") that it would not have generated otherwise. If creating the offspring gives a net benefit to the parent, then we conclude that natural selection favours a small increase to the duplication rate. This can be recast in a social evolution framework using Hamilton's rule[58]. In this framing, natural selection favours an increase to the duplication rate when

$$rB > C \,, \tag{1}$$

where r is the genetic relatedness between parent and offspring transposons, B is the number of new offspring that result from the higher duplication rate, and C is the cost to the parent of the higher duplication rate.

Under public replication, we have $r = 1/\bar{n}$ because that is the probability that the parent duplicates itself and not a wild-type transposon sharing the same genome; under private replication, we have $r = 1$ because the parent necessarily duplicates itself. The benefit B is 1 under both public and private replication, because we are focusing on an instance where the parent creates one new transposon owing to its marginally higher duplication rate. And the cost C is the detriment to the parent transposon's fitness that it causes by "polluting" its host's genome with one additional transposon. If the host fitness function is $w(n)$, then the host's relative fitness can be written $W(n) = w(n)/\bar{w}$, where $\bar{w}$ is the average host fitness in the population. This means that a host pays a fitness penalty $\mathrm{d}W/\mathrm{d}n$ for every increase to its transposon number by one. At equilibrium, $\mathrm{d}W/\mathrm{d}n = -u$, because selection against the deleterious effects of

transposons must counterbalance their proliferation rate[24]. This penalty is paid by the parent transposon for every generation that it stays associated with the offspring, which is two generations on average in a sexually-reproducing host species, because meiosis has a 50% probability of dissociating parent and offspring each generation. Accordingly, $C = 2u$ under both public and private replication.

Substituting these values for *r*, *B*, and *C* into condition (1) shows that natural selection favours an increase to the duplication rate *u* under public replication when

$$u < \frac{1}{2\bar{n}}, \tag{2}$$

and favours a small increase to the duplication rate under private replication when

$$u < \frac{1}{2}. \tag{3}$$

This is an improvement upon the previous version of the manuscript because it now stands on its own as an intuitive argument.

In the Methods, we now state:

2.2 Evolutionary logic — We conducted an invasion analysis of our deterministic model to derive a mathematical condition that governs whether an increase to the transposon duplication rate is favoured or disfavoured by natural selection. (While some authors restrict the term "natural selection" to forces acting at the level of the individual organism, we consider natural selection more broadly as operating on any entities that display heritable variation in fitness[74]). The informal derivation of conditions (2) and (3) in the *Results* makes three key simplifying assumptions, namely that (i) variation in the transposon number *n* among genomes is negligible; (ii) the transposon duplication rate *u* is small; and (iii) natural selection at the host level acts slowly compared to the effect of meiosis in breaking up associations between transposons. We relax these assumptions in *Supplementary Methods* 2, where the full invasion analysis is presented. This more robust analysis recovers conditions (2) and (3) of the *Results* in the limit of large $\bar{n}$ (which decreases the relative variability between hosts in transposon number) and small *u* (which in turn implies relatively weak selection at the host level).

For the numerical simulations illustrating evolutionarily stable strategies for the transposon duplication rate (Fig. 2), we first used the deterministic model with one transposon type to map out a "fitness surface" by running the model to equilibrium across a grid of different values for the fitness cost *c* and duplication rate *u*; the equilibrium host fitness $\bar{w}$ furnishes the shaded background areas for Fig. 2. Then, across a grid of values for the fitness cost *c*, we repeatedly chose values of *u* and ran the deterministic model with two transposon types to determine whether a mutant transposon with duplication rate *u* + 0.0001 could invade. We used a binary search to find, for each value of *c*, the value of *u* at which a faster-duplicating mutant was no longer favoured to invade. This is the equilibrium level of *u* found through numerical simulation, which is plotted in Fig. 2 as a solid line. The dashed line is the equilibrium *u* identified by the full invasion analysis (*Supplementary Methods* 2), while the dotted line

> is the equilibrium *u* identified by the simplified analysis in the main text (conditions (2)–(3)).

The first paragraph above now states exactly how conditions (2) and (3) in the main text are an approximation, justifying the need for the full invasion analysis. The second paragraph is also new and gives new detail on how Fig. 2 was generated.

The full invasion analysis remains of substantial value because it reveals when the approximations in (2) and (3) can be expected to work and when they cannot, and because it is what justifies the simplified argument presented in the Results, which would otherwise just be a conjecture. The full invasion analysis may also help to make further theoretical innovations in future work.

2. Methodological details are missing to interpret the simulations. In particular, figure panels report a single line + shaded area around, but there is no explanation not consistency across figures. Do the panels report single simulation runs + variation in the population, or multiple simulation runs + variation among replicates?

We now report on each relevant figure that the line and shaded area represent the mean and 2.5% to 97.5% quantile across the population for a single simulation run. This is consistent across all figures with "Generations" on the x-axis.

Note that we do show that our results are consistent across multiple simulation runs.

Scenarios in panels 1G and 1I are clearly manipulated for instance, which is very different from other panels were populations evolved freely.

We now have clarified that these panels (we think the reviewer meant 1F and 1I) use the deterministic model, which can simulate two transposon types at a time. We also clarify in the Methods (2.1):

> Note that Fig. 1I uses the deterministic model, which only allows consideration of two transposon types at a time; we can show competition among five successive transposon types with different duplication rates using this model because we successively replaced the outcompeted transposon type with a new, more competitive type.

I found extended data 1 a bit worrying, because it shows that single simulation runs can be strongly affected by stochasticity (results are very unconsistent for p=0.94, p=0.95, p=0.96 and p=0.97, for instance). This excludes the strategy of considering single simulation runs, at least for the "parasitism" case; the variable of interest is not the copy number, but rather the probability of TE elimination.

(Note that what was previously Extended Data Fig. 1 is now Extended Data Fig. 3).

We have now ensured that each result of the manuscript that depends on the stochastic model is backed up by multiple runs, and that this is stated clearly. In particular, Fig. 3D and Extended Data Fig. 2 report the mean across three replicates, Extended Data Figs. 3 and 4 report results across 10 replicates for each parameter

combination, and Extended Data Fig. 5 reports similar outcomes across 3 replicates for each parameter combination.

I also doubt that the raw time series from the simulations is always the best representation of the simulation results, especially because it makes the exploration of the parameter space very cumbersome (as in extended figures). For instance, in Ext fig 1, the variable to be reported should be the probability of extinction as a function of p (and possibily other parameters), for fig 3 it could be the time before extinction, etc. This would allow a substantial simplification of the panels, as well as the possibility to compare different factors (e.g. private vs public transposition in fig 3).

We have revised the exploration of parasitism (now Extended Data Fig. 3) and of suppression (now Extended Data Fig. 4) to show ten replicates per parameter combination and these figures now show first a set of single example runs, then summaries of the evolutionary outcome across ten replicates per parameter combination.

For Fig. 3D, we originally showed the mean host fitness at equilibrium, with 0 representing extinction. The reviewer suggested that we change this to show mean time before extinction instead, but we don't think this is more meaningful than showing the equilibrium mean fitness, so we have kept Fig. 3D as it is. We have, however, added a figure showing the effect of different cost functions under private versus public transposition side-by-side (Extended Data Fig. 2), so now the comparison requested by the reviewer regarding Fig. 3 can easily be made.

3. The modeling choice to consider private/public transposition as discrete binary strategies should be discussed more, and possibly turned into a quantitative parameter similar to parameter p for the parasitic replication. When considering for instance Zhang et al 2020, cited in the manuscript, the Ka/Ks ratio used as a measurement for cis-preference seems to span uniformly between 0.2 (close to functional proteins, and probably close to the "100% private case) and 1.0 (neutrality, "public"). This suggests that most TE families are somehow mid-way between public and private, while very few TE families behave as in the simulated model. In particular, the description of the "parasitic" model was not trivial in the "private" context, because it requires a breakage of the "private transposition" principle, but only towards non-autonomous copies. Considering that imperfect privacy is a prerequisite for the emergence of non-autononous TEs thus seems more natural, and would open new interesting issues, such as the level of privacy from which the ever-increasing transposition rate could start.

We agree with the reviewer that these are interesting points, but exploring private versus public transposition as a continuous parameter is beyond the scope of this paper. We have treated private versus public replication as discrete strategies, and this is motivated not primarily by the Zhang et al paper, but by biomolecular details which point strongly towards public replication for DNA transposons and private replication for LINE transposons. We have added a paragraph to the Discussion to reinforce that LTRs in particular may exhibit intermediate strategies and that this is a promising area for future work:

We have treated public versus private replication as discrete strategies for transposon replication, which is motivated by the molecular biology of replication for DNA versus LINE transposons in particular. Evidence of private replication in LTR transposons is less clear-cut[27,53-56]. It is possible that LTR transposons may exhibit intermediate levels of "privacy" in replication, and future work could explore the resulting evolutionary dynamics.

** Minor comments

4. In the abstract and later in the manuscript, the authors attribute the never ending increase of the transposition rate as a case of tragedy of the commons. Although competitive tragedy might somehow be related to tragedies of the common, I am not completely sure it is the same phenomenon. Paradoxically, I would tend to think that what happens to public transposition copies is closer to the tragedy of the commons, because public TEs have no incentive to participate to the pool of transposition machinery, which degrades their ability to code for a functional protein.

The reviewer raises a useful point about the terminology, which allows us to clarify how we are using the term "tragedy of the commons". In our framework, the unbounded increase in duplication rate for private elements corresponds closely to the classical tragedy-of-the-commons structure, in that individual elements benefit by exploiting a shared genomic resource, and this element-level advantage generates a collectively deleterious outcome at the level of the genome, eventually compromising host viability if left unchecked. It is this unrestrained misalignment between individual- and collective-level interests that motivates our use of the term.

The situation with public transposable elements is related but conceptually distinct. Public elements rely on transposition machinery produced by other copies, thereby avoiding the costs associated with maintaining functional coding capacity themselves. This behaviour is more naturally described as cheating or free riding within a cooperative system. While such cheating exploits the contributions of others, it does not by itself constitute a tragedy of the commons, since it need not, on its own, lead to systematic degradation of the shared resource. These two phenomena can, of course, coexist within the same genomic system, and both reflect failures of collective organisation at different levels.

Later in the manuscript, the authors compare the threshold from which selection promotes higher duplication rates to Hamilton's rule, related to social behavior. This seems conceptually close to the attempts at comparing TE interactions to ecological interactions (Venner, S., Feschotte, C., & Biemont, C. (2009). Dynamics of transposable elements: towards a community ecology of the genome. Trends in Genetics, 25(7), 317-323. and other related papers). As far as I can tell, these comparisons are only vague analogies, which do not really catch the complexity of TE interactions. Perhaps it would be worth discussing the differences between these two similar analogies, and what the social behavior analogy brings to the ecological analogy?

The reviewer is right that our social evolution framing in terms of Hamilton's rule has parallels to earlier attempts to understand transposon biology using ideas from

community ecology. There are, however, some key differences between our paper and previous work.

In general, transposable elements have repeatedly been analysed through conceptual frameworks imported from other areas of biology and beyond, including host–parasite interactions (Nee and Maynard Smith 1990), political philosophy (Okasha 2012), multilevel selection theory (Gregory 2013), and epidemiology (Wagner 2009), These approaches differ, however, in what they are designed to explain. The community-ecology analogy of Venner et al (2009), for example, has primarily been used descriptively, to highlight patterns of coexistence, diversity, and distribution of elements (see e.g. Linquist et al. 2015).

In principle, community-ecological and the kind of social-evolution models we use in our analysis can be linked mathematically. It has long been known that the classical Lotka–Volterra equations at the heart of community ecology can be rewritten as frequency-dependent selection or replicator dynamics, which in turn provide the standard mathematical foundation of evolutionary game theory. These links, however, have not really been done for transposons, and, to our knowledge, the community ecology framework has generally not been used to quantify the evolutionary dynamics of transposons in a formal, non-verbal, way. By contrast, the connection to Hamilton's rule that we establish in our model is not a heuristic analogy, but a formal result extending from our in-depth mathematical analysis.

Gregory TR. 2013. Molecules and macroevolution: a Gouldian view of the genome. In GA Danieli, A Minelli, and T Pievani, eds. Stephen J. Gould: The Scientific Legacy, pp. 53–72. Springer.

Linquist S, Cottenie K, Elliott TA, Saylor B, Kremer SC, and TR Gregory. 2015. Applying ecological models to communities of genetic elements: the case of neutral theory. Molecular Ecology. 24: 3232–3242.

Nee S and J Maynard Smith. 1990. The evolutionary biology of molecular parasites. Parasitology. 100: 5–18.

Okasha S. 2012. Social justice, genomic justice and the veil of ignorance: Harsanyi meets Mendel. Economics and Philosophy. 28: 43–71.

Wagner A. 2009. Transposable elements as genomic diseases. Molecular Biosystems. 5: 32.

5. Line 186 and other places in the manuscript: I was confused about the authors' use of "natural selection" in this context. If my undertsanding is correct, the authors wish to compute the equivalent of inclusive fitness for a TE copy, accounting for both Darwinian selection at the individual level (decrease in the transmission rate across generations) and intragenomic selection (increase in copy number due to replicative transposition). This probably makes sense, but I am not sure "natural selection" properly catches the idea. I am not sure a consensual definition of "natural selection"

exists, but I would typically exclude non-phenotypic selection (e.g. selfish DNA) from natural selection.

We think there are good reasons not to restrict the term 'natural selection' to any particular biological level. While natural selection is often understood as acting on individual organisms, the logic can be defined in more general terms. Whenever a population of entities exhibits variation, when that variation is associated with systematic differences in reproductive success, and when those differences are heritable, the composition of the population will change over time, all else being equal. This idea is captured in Lewontin's classic formulation, according to which natural selection operates on any entities that display "heritable variation in fitness" (Lewontin 1970, now cited in the manuscript). Transposable elements meet these criteria, as do a wide range of biological entities including genes, organelles, cells, multicellular organisms, and, in some circumstances, groups and species.

To address this point in the manuscript, we have placed a brief note in the Methods section, with a reference at the first mention of "natural selection" in the Results section:

> Why does the mode of transposon replication (public versus private) lead to such fundamentally divergent outcomes? To answer this question, we conducted an invasion analysis of our deterministic model. This method allows us to derive a mathematical condition to explain when an increase to the transposon duplication rate is favoured by natural selection (see *Methods* 2.2).

Methods 2.2 starts with:

> *2.2 Evolutionary logic* — We conducted an invasion analysis of our deterministic model to derive a mathematical condition that governs whether an increase to the transposon duplication rate is favoured or disfavoured by natural selection. (Note that while some authors restrict the term "natural selection" to forces acting at the level of the individual organism, we understand natural selection more broadly as operating on any entities that display heritable variation in fitness[74]).

We considered placing this note in the main text, but it felt distracting.

6. Around line 324 and in other related sections, it should be noted that the "evolutionary race" is only possible when considering that transposition rates could increase forever. If the transposition rate was physically of biochemically bounded, the race could stop (although population extinction may still happen sooner or later because of the genetic load).

We had material in the Discussion already that was relevant to this point, and we now more clearly raise the objection made by the reviewer:

> An associated prediction of our model is that conflict between hosts and transposons will lead to a high level of transposon expression and a high level of suppression, resulting overall in low duplication rates. **Here, a potential**

criticism of the model is that it assumes that both host and parasite can continually escalate their arms race. Clearly, biochemical constraints will eventually prevent transposons from adopting arbitrarily-high duplication rates, and hosts from suppressing transposition with complete precision. Nonetheless, empirical findings support that transposons can be very highly transcribed.

7. Line 421, the equation is not correct in the manuscript, because i and j should run from 0 to Inf (and not to n). Note that the equation is correct in the supplementary methods. Moreover, U1 and U2 are not defined here.

We thank the reviewer for spotting these mistakes. This section of the Methods is no longer in the manuscript, so only the correct version in the supplement remains.

8. Line 457, is the mean number of chiasma per chromosome or for the whole genome?

It is the number of chiasmata per chromosome; we have fixed this omission.

We thank the reviewer for these constructive comments.

Reviewer #2 (Remarks to the Author):

The paper by Davies, Agren and Foster describes a theoretical work about the evolution of the transposition rate of transposons given a public or private mode of replication. It is a very interesting work pointing out that we expect different evolutionary outcomes for TE families that may replicate publicly and privately. Also the parallels to kin-selection (Hamiltons rule) and the tragedy of the commons are fascinating. Additionally the level of scholarly is impressive, where they for example point out the six different negative effects that TEs may have (I was solely aware of three). Finally I have to say that the manuscript is amazingly well written. Concepts are nicely explained, with illustrative figures and the style makes it easy to follow.

We appreciate this positive assessment by the reviewer.

However it is a theoretical study and as such has the typical problem of any theoretical work, i.e. it is not clear how well the models and assumptions reflect the actual biology of the organisms and the TEs, and thus how relevant the work is to the field. Nevertheless I consider it an important contribution.

I have some major/minor comments

major
- The six different negative effects of TEs are interesting. However I think modelling the effects of all of them is excessive, and perhaps conveys the wrong impression that we understand these processes well enough so that we are able to accurately model them. I would argue that our knowledge about the biology of TEs is currently far too limited to attempt this and therefore a simple (or a few simple) and more general models of negative selection would have been entirely sufficient.

We agree with the reviewer that our understanding of fitness costs is incomplete. But if we were to remove these estimates from our paper in favour of more general models of negative selection, we would fail to address the question of whether strong negative selection might avert the tragedy of the transposons in practice, not just in theory.

We think that there are several advantages to including these cost estimates:

(1) We collect these estimates in one place. The reviewer themselves noted that they were only aware of three of these potential costs.

(2) We can eliminate four of the six costs as being implausibly weak to explain equilibrium transposon numbers **when we assume the duplication rate is fixed**. Only two of the costs could possibly keep transposon numbers within observed ranges. This was not strongly apparent in the previous version of the manuscript. We now show this result in a new Extended Data Fig. 1 by comparing theoretical expectations to empirically observed copy numbers in Drosophila. This is explained in some new material in the Results:

We find that none of the empirically-supported cost functions we identified is strong enough to prevent population collapse (Fig. 3C). **This outcome is partly because many of these costs are inherently weak. Indeed, we find that four of the six costs proposed in the literature (costs iii-vi) are too weak to meaningfully inhibit transposon proliferation, at least when acting on their own (Extended Data Fig. 1). This result has likely gone unnoticed because previous studies have only identified these as potential costs, but did not estimate the strength of selection that would be associated with them. The two remaining costs—ectopic recombination (cost i) and deleterious insertions (cost ii)—could potentially keep transposon number in check, if the duplication rate were fixed.**

Extended Data Fig. 1 shows:

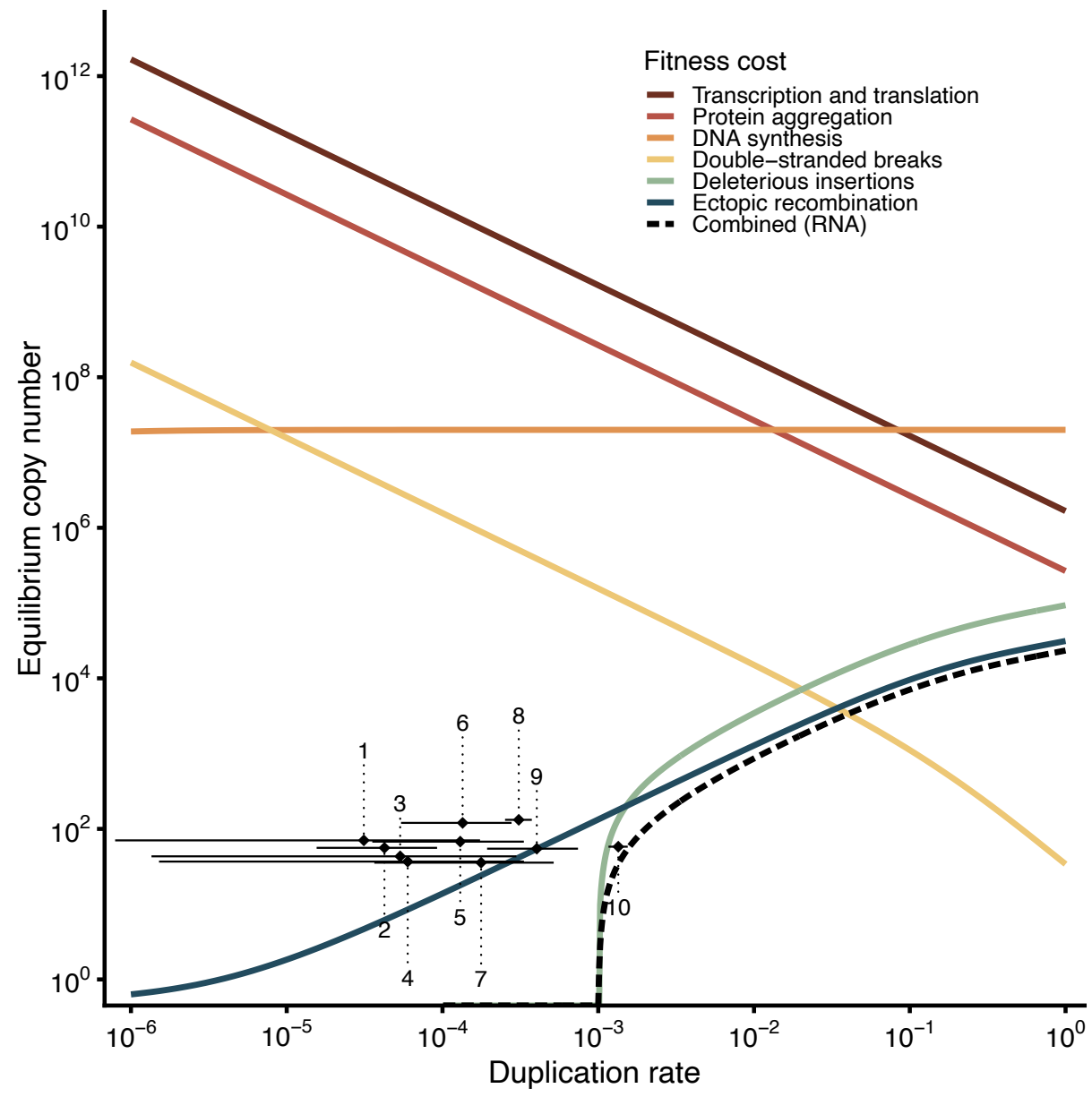

Extended Data Fig. 1. Strength of each identified fitness cost mechanism relative to empirical estimates for *Drosophila*. There are substantial differences in the relative strength of each cost mechanism, which we illustrate by plotting the expected equilibrium transposon number across a range of duplication rates for each cost. Numbered points with error bars show the estimated duplication rate and number of transposon copies found in the *Drosophila* genome from two studies: (1) *jockey*[78]; (2) *Doc*[79]; (3) *2244*[78]; (4) *opus*[78]; (5) *297*[78]; (6) *roo*[78]; (7) *mdg-1*[78]; (8) *roo*[79]; (9) *copia*[78]; (10) *copia*[79].

(3) We can argue that it is not plausible that any of these costs can prevent host extinction **when we assume the duplication rate can evolve**. We acknowledge that we may not have captured these costs perfectly, but that is why we test inflating these cost estimates by several orders of magnitude before concluding that they are not a plausible mechanism for preventing host collapse.

For example we do not even understand the simplest model, i.e. the direct effect of the TE insertion. They assumed that all insertions have equal fitness effects, but this is likely not true. Some could be neutral, others slightly deleterious, a few deadly and

even fewer positively selected. So there is an unknown distribution of fitness effects of TE insertions that will likely differ among the families.

While developing the model, we tested various different models which combined sites with neutral effects, moderate effects and severe effects on fitness. We found similar results to Charlesworth (1991: Transposable elements in natural populations with a mixture of selected and neutral insertion sites, *Genet. Res., Camb*. 57: 127–134), namely that when there are sites with weak or neutral effects, transposons just accumulate uncontrollably in those sites. The overall effect (also found by Charlesworth) is that variability in fitness effect by site tends to make the transposon number harder to control — so our simplified fitness function actually is more conservative than a more detailed function with respect to what we are trying to show in this section. We have added back in a model where fitness effects are either mild or severe (Extended Data Fig. 2) to show that this does not make much difference.

Moreover, they assumed that the fitness impact of TEs increases exponentially with TE copy numbers (n^2 in Fig2 caption). This is of course in agreement with the model of Charlesworth1984 which requires an exponential increase in fitness cost to obtain a stable TE equilibrium (without host defence). But it is entirely unclear if this assumption holds.

We do not think that host suppression changes the requirement that fitness costs are synergistic. But to make this assumption clearer, we have expanded upon this in the Results:

> For each of the six fitness costs, we constructed a dedicated host fitness function parameterized with data collected from the literature (*Methods* 2.3). We also constructed a "combined" fitness function incorporating all five costs relevant for RNA transposons (i.e., excluding the cost of double-stranded breaks), since only RNA transposons use private replication. **For each function, we assumed that the relative burden to the host of each additional transposon increases as more transposons accumulate (i.e., each function is log-concave with respect to transposon number). This relationship was established by Charlesworth and Charlesworth[24] as a necessary condition for the transposon number to reach equilibrium, assuming a fixed duplication rate.**

Next, it is not clear what are the fitness effects of homozygous vs heterozygous insertions and how they epistatically interact. As another example, with ectopic recombination we have again so many unknowns. does the rate of ectopic recombination depend on the recombination rate, which varies through the genome (which is not considered in the manuscript) , what is the distribution of fitness effects of ectopic recombined chromosomes;

We think the effect of the recombination rate varying by site would have a similar effect to deleterious insertion effects varying by site, which we have now added to the manuscript.

does the rate of ectopic recombination depend on the length of the TEs (I do not think this was considered).

This is not correct; our ectopic recombination fitness function explicitly includes a term for the length of the transposon.

Do we have more ectopic recombination for heterozygous than for homozygous insertions?

We also tested this during model development. We found that most TE insertions were heterozygous in large enough populations, so this did not have a large effect either way.

How is the model affected if the rate of ectopic recombination is very heterogenous in the genome? And similarly long lists of unknowns can be made for all 6 negative effects of TEs. Therefore I think it is a bit excessive trying to model these many negative effects, and it conveys a wrong sense of accuracy.
Just to be clear I am not suggesting to make their model more complex but rather the opposite, to perhaps limit it to a very few ones that capture the main message.

We appreciate that the reviewer is (correctly) pointing out that there are many unknowns. We have responded to this general point by clarifying why we have tried to ground our fitness functions in estimates from the literature, and by adding Extended Data Fig. 1 we have clarified that putting a rough magnitude on these various costs that have been proposed represents a genuine advance even if the details are uncertain.

- In a similar direction I think the conclusion that TGS cannot prevent extinction and PTGS is necessary to stop TEs, is a pure artifact of their assumptions. I do not see how an arms race is a necessary outcome of TGS. For example, in Drosophila, a TE insertion in a piRNA cluster leads to piRNAs that direct TGS and PTGS. Any mutation in the TE leads to mutated piRNAs, that attack the mutated TE. So I do not see how this can possibly lead to an armsrace between the TE and the host. Additionally I do not see how this adaptation to the mutated TE increases the cost of TGS (the same amount of piRNAs, the same amount of silenced loci etc).

We are assuming that if the transposon increases its expression level, this will result in more transcripts being produced. If the host's expenditure of energy for suppression stays the same, all else equal it will degrade the same *fraction* (not number) of the transposon's transcripts. If the host needs to degrade a larger proportion of the transposon's transcripts, it will have to spend more energy. This is in keeping with standard assumptions about mass action in chemical systems.

Also as far as I can tell they assume a fitness cost for TGS but not for PTGS, so no surprise this PTGS which does not incur any fitness cost seems necessary to stop TEs. Therefore this conclusion is just an outcome of their model assumptions, which are to my knowledge not supported by biology.

This is not correct; we assume that both suppression methods incur the same fitness cost to the host. As we already say in the Methods section: "We assign a fitness cost of 10^{-7} for each unit of suppression, which is enough to select against needless

suppression without preventing hosts from adequately suppressing transposons. This assumes that transcriptional and post-transcriptional suppression are equally costly, which may not be the case."

Furthermore its a bit unintuitive to me. In my mind, TGS must be way less costly to the host than PTGS, since TGS prevents the transcription of TEs, and thus saves resources. While in PTGS, the TEs are transcribed and then degraded, a very wasteful processes that spends a lot of energy (which the authors mention in the manuscript).

The reviewer's intuition is correct, and our model accounts for this. In our model, both suppression itself and any transcription/translation of TEs incur separate costs to the host.

- the finding that a host defence can prevent extinction of host populations was already previously shown, even with constant transposition rates. I think this should be acknowledged https://academic.oup.com/mbe/article/36/7/1457/5435964

We do already cite this study in our introduction. However, our focus is on what mechanisms can prevent host extinction in the light of an evolving duplication rate. The study mentioned by the reviewer assumes that the duplication rate is fixed, and so we don't think it is getting at quite the same problem.

minor
- Just as a possibility, I leave this to the discretion of the authors: I would move the description on which TE families are public (DNA transposons) and which are private (non-LTR and perhaps LTR) to the introduction. Initially, when reading the manuscript I assumed they make a straw-man argument, distinguishing between public/private with little biological examples for each category. Only in the results it became clear to me that some TE families are clearly public while others are very likely private, so the distinction between public/private TE families is an important one, supported by many biological examples.

We agree that this was originally clarified too late in the manuscript. We now state in the Abstract: "Publicly-replicating transposons **(such as DNA transposons)**, which cooperatively share their replication machinery, are predicted to be self-limiting. However, privately-replicating transposons **(such as LINEs)**, which do not replicate cooperatively, are under continual selection to increase their duplication rate even to the point of host extinction, a so-called tragedy of the commons."

- one testable prediction of their model could be that private TEs should have evolved a higher transposition rate than public ones? Or is this solely expected when TEs are kept in check just by negative selection (as in Fig2). Do we also expect this under models with a host defence scenario? As far as I can tell the effect of the host defence was only modeled for private TEs (as extinctions only occured for those).

This is not correct; our original submission already included simulations of host defence for public TEs, as Extended Data Fig. 4 (Host suppression under public replication). This is still present in this version of the manuscript as Extended Data Fig. 5.

Perhaps the authors can add to the discussion a paragraph about testable hypothesis arising from their work?

This is an excellent suggestion. We have added to the Discussion:

> It is possible that LTR transposons may exhibit intermediate levels of "privacy" in replication, and future work could explore the resulting evolutionary dynamics. **A prediction of our model is that hosts are selected to suppress privately-replicating transposons more highly than publicly-replicating transposons (Extended Data Figs. 4, 5). In this case, hosts may apply an intermediate level of suppression to LTR transposons.**

- eg line 546, I think the dashed square is a LaTeX error?

Yes, the dashed square is just a placeholder from Microsoft Word's equation editor. We will make sure these do not appear in the final proof.

- initially I assumed that public refers to interactions between different two TE families. But nevertheless, I was wondering could this happen, i.e. that some proteins produced by one family are then used by another one? Would this have any influence on their model predictions? Perhaps this can be a bit discussed. Or is this perhaps just a 'special' scenario of the non-autonomous insertion scenario which they modeled - where the other family is basically like the non-autonomous parasite?

We are not aware of any real-life examples of transposition machinery being shared across unrelated families. Instead, there are either nonautonomous elements within DNA families or parasitic elements within LINE-1 families; our model already includes both of these.

- 271 and further: references missing about non-autonomous TEs parasitizing the autonomous ones

We have added references to Burt & Trivers' 2006 book, and papers by Bennett et al. 2008 and Ahl et al. 2015 to this section.

- 310 'piRNAs silence TEs through DNA methylation'. This is not correct, e.g. Drosophila has no methylation, yet TGS silences TEs (by epigenetic histone modifications).

We have corrected this mistake as follows: "For example, the Piwi-piRNA pathway in animals[16-19] performs sequence-specific silencing of transposons through both **transcriptional suppression (e.g. by DNA methylation or histone modification) and post-transcriptional suppression (by degradation of mRNA).**"

We thank the reviewer for these constructive comments.
